# Tislelizumab plus zanubrutinib for Richter transformation: the phase 2 RT1 trial

**DOI:** 10.1038/s41591-023-02722-9

**Published:** 2023-12-09

**Authors:** Othman Al-Sawaf, Rudy Ligtvoet, Sandra Robrecht, Janina Stumpf, Anna-Maria Fink, Eugen Tausch, Christof Schneider, Sebastian Boettcher, Martin Mikusko, Matthias Ritgen, Johannes Schetelig, Julia von Tresckow, Ursula Vehling-Kaiser, Tobias Gaska, Clemens Martin Wendtner, Bjoern Chapuy, Kirsten Fischer, Karl-Anton Kreuzer, Stephan Stilgenbauer, Philipp Staber, Carsten Niemann, Michael Hallek, Barbara Eichhorst

**Affiliations:** 1 Department I of Internal Medicine and German CLL Study Group; Center for Integrated Oncology Aachen Bonn Cologne Duesseldorf (CIO ABCD), University of Cologne, Faculty of Medicine and University Hospital of Cologne, Cologne, Germany; 2 Francis Crick Institute London, London, UK; 3 Cancer Institute, University College London, London, UK; 4 Department III of Internal Medicine, University Hospital Ulm, Ulm, Germany; 5 Department III of Internal Medicine, University Hospital Rostock, Rostock, Germany; 6 Department of Haematology and Oncology, Otto-von-Guericke University Magdeburg, Magdeburg, Germany; 7 Department II of Internal Medicine, Campus Kiel, University of Schleswig-Holstein, Kiel, Germany; 8 Department I of Internal Medicine, University Hospital Carl Gustav Carus, Dresden, Germany; 9 Clinic for Hematology and Stem Cell Transplantation, West German Cancer Center, University Hospital Essen, University of Duisburg-Essen, Essen, Germany; 10 MVZ Dr Vehling-Kaiser, Landshut, Germany; 11 Department of Hematology and Oncology, Brüderkrankenhaus St. Josef, Paderborn, Germany; 12 Department of Medicine III, University Hospital, LMU Munich, Munich, Germany; 13 Department of Hematology and Medical Oncology, Georg-August University Göttingen, Göttingen, Germany; 14 Department of Hematology, Oncology, and Cancer Immunology, Charité -University Medical Center Berlin, Campus Benjamin Franklin, Berlin, Germany; 15 Department of Internal Medicine I, Division of Hematology and Hemostaseology, Medical University of Vienna, Vienna, Austria; 16 Department of Hematology, Rigshospitalet, Copenhagen, Denmark

**Keywords:** B-cell lymphoma, Cancer therapy, Chronic lymphocytic leukaemia, Cancer immunotherapy

## Abstract

In patients with chronic lymphocytic leukemia, Richter transformation (RT) reflects the development of an aggressive lymphoma that is associated with poor response to chemotherapy and short survival. We initiated an international, investigator-initiated, prospective, open-label phase 2 study in which patients with RT received a combination of the PD-1 inhibitor tislelizumab plus the BTK inhibitor zanubrutinib for 12 cycles. Patients responding to treatment underwent maintenance treatment with both agents. The primary end point was overall response rate after six cycles. Of 59 enrolled patients, 48 patients received at least two cycles of treatment and comprised the analysis population according to the study protocol. The median observation time was 13.9 months, the median age was 67 (range 45–82) years. Ten patients (20.8%) had received previous RT-directed therapy. In total, 28 out of 48 patients responded to induction therapy with an overall response rate of 58.3% (95% confidence interval (CI) 43.2–72.4), including 9 (18.8%) complete reponse and 19 (39.6%) partial response, meeting the study’s primary end point by rejecting the predefined null hypothesis of 40% (*P* = 0.008). Secondary end points included duration of response, progression-free survival and overall survival. The median duration of response was not reached, the median progression-free survival was 10.0 months (95% CI 3.8–16.3). Median overall survival was not reached with a 12-month overall survival rate of 74.7% (95% CI 58.4–91.0). The most common adverse events were infections (18.0%), gastrointestinal disorders (13.0%) and hematological toxicities (11.4%). These data suggest that combined checkpoint and BTK inhibition by tislelizumab plus zanubrutinib is an effective and well-tolerated treatment strategy for patients with RT. ClinicalTrials.gov Identifier: NCT04271956.

## Abstract (web summary)

In a large single-arm phase 2 trial, the anti-PD-1 inhibitor tislelizumab combined with the next-generation BTK inhibitor zanubrutinib had an overall response rate of 58.3% and was well tolerated in patients with Richter’s transformation.

## Main

Chronic lymphocytic leukemia (CLL) is classified as an indolent B cell non-Hodgkin lymphoma according to the World Health Organization classification and is the most common type of leukemia in adults^1^. RT (also known as Richter’s syndrome) describes the development of an aggressive lymphoma developing in patients with CLL, most commonly a diffuse large B cell lymphoma (DLBCL) or Hodgkin’s lymphoma (HL)^2,3^. The incidence rates of RT among patients with CLL range from 2 to 10%^4^. RT can occur at any time during the course of CLL, though occurrence in treatment-naive CLL is less frequent than in pretreated CLL. Patients with RT have a dismal prognosis with chemoimmunotherapy such as R-CHOP, with overall response rates (ORR) of <40% and median overall survival of 6–8 months^4,5^. Targeted therapies have the potential to improve outcomes, but few prospective studies have been run in this entity so far. Previous reports suggested efficacy of monotherapy with checkpoint inhibitors, BTK inhibitors, BCL-2 inhibitors and PI3K inhibitors^6–10^, but sample sizes were limited and potentially underpowered for conclusive results.

Programmed cell death protein 1 (PD-1) is an important immune-checkpoint receptor that is predominantly expressed on activated T cells and transmits inhibitory signals into T cells after ligation with PD-L1 or PD-L2 on malignant cells and the tumor microenvironment^11,12^. Immunotherapy via blockade of PD-1 or PD-L1 has demonstrated high efficacy and has become an established component for the therapy of multiple cancers^13,14^. In the context of RT, PD-1 expression has been reported as a common feature^15^ and several preclinical models have suggested a susceptibility of RT to checkpoint inhibition^16,17^.

Though checkpoint inhibitors are promising candidates for treatment of RT, single-agent treatment with PD-1 inhibitors did not prevent progression of CLL^18^, suggesting that a combinational approach might be needed to target both the aggressive and indolent components of RT.

BTK inhibitors have become a cornerstone of CLL therapy, as B cell receptor (BCR) signaling is a key dependency of CLL cells that is required to sustain prosurvival signals from the microenvironment^19^. Several BTK inhibitors are available for the treatment of CLL and have demonstrated good long-term efficacy^20^. In the context of RT, preclinical models have demonstrated BCR signaling dependency that suggests sensitivity to BCR inhibitors^21^. This was further substantiated in translational studies that outlined the role of BCR signaling in RT samples^22,23^; however, monotherapy of RT with BTK inhibitors such as acalabrutinib or pirtobrutinib monotherapy offered only short-term disease control, supporting the need for combination strategies^9,24^.

Based on preclinical and translational data that suggested immune-checkpoint inhibition and BTK inhibition as possible vulnerabilities of RT, we hypothesized that a combination of tislelizumab and zanubrutinib could be an effective strategy to induce remissions in patients with RT, who are treatment-naive or have received up to one previous line of RT-directed therapy. Tislelizumab is a humanized, immunoglobulin G4-variant monoclonal antibody against PD-1 that has been explored in solid malignancies and has demonstrated low rates of immune-related adverse events and good efficacy. Tislelizumab has previously demonstrated efficacy in a variety of solid malignancies, including in the first-line treatment of advanced non-small cell lung cancer and esophageal cancer^25–28^. Zanubrutinib is a next-generation, covalent BTK inhibitor that has demonstrated limited off-targeted effects and thereby less toxicity and higher efficacy than the first-in class BTK inhibitor ibrutinib in patients with relapsed or refractory CLL^29,30^.

Here, we present data of the international, investigator-initiated phase 2 RT1 trial, in which the PD-1 inhibitor tislelizumab was combined with the next-generation BTK inhibitor zanubrutinib to treat patients with RT, with the objective to compare the ORR after six cycles with the prespecified benchmark of 40%.

## Results

### Trial design and patients

**Fig. 1:**
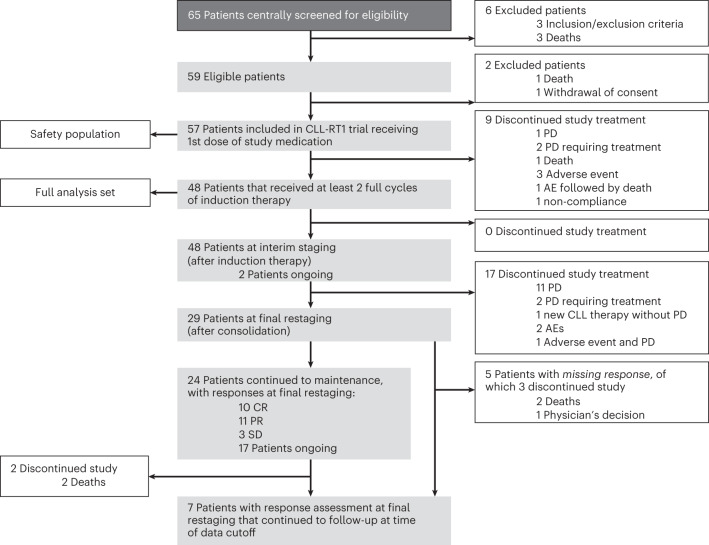
CONSORT diagram of RT1. PD, progressive disease; AE, adverse event.

Between 11 February 2020 and 2 January 2023, 65 patients were screened, of which 59 were enrolled. Of those, two did not receive study medication owing to death (one patient) and withdrawal of consent (one patient) and nine discontinued study treatment within the first two cycles owing to primary progressive disease (three patients), death (one patient), adverse events (four patients) and non-compliance (one patient). According to the study protocol, 48 patients who received at least two cycles of study treatment, including at least one administered dose in cycle three, comprised the analysis population (Fig. 1). The primary end point was ORR at interim staging and the secondary end points included ORR after consolidation therapy, duration of response (DOR), progression-free survival (PFS), overall survival, time to next treatment (TTNT) and safety. Post hoc analyses included ORR and time-to-event parameters in the intention-to-treat (ITT) population, ORR and PFS according to previous RT-directed therapy and according to previous BTK inhibitor exposure.

The median age was 67 (range 45–82) years, 29 (60.4%) patients were male and 19 (39.6%) were female (Table 1). Twenty-two (45.8%) patients had Eastern Cooperative Oncology Group (ECOG) performance status of 1 or higher and the median cumulative illness rating scale (CIRS) score was 4 (range 0–17). The median LDH at enrollment was 335 U l^−1^. Sixteen (34.8%) patients had del(17p)/*TP53*mut, whereas 29 (70.7%) patients had unmutated IGHV. Overall, 25 (64.1%) patients had high or very high-risk CLL according to the chronic lymphocytic leukemia international prognostic index (CLL-IPI), 11 (28.2%) had intermediate risk and 3 (7.7%) had low risk. Complex karyotype (≥3 aberrations) was detected in 16 (42.1%) patients. A total of 46 (95.8%) patients had DLBCL-RT and 2 (4.2%) patients had HL-RT. In those 26 patients (54.2%) in whom clonal relatedness was evaluated by immunoglobulin heavy chain rearrangement analysis, all cases were reported as clonally related to the CLL (22 (45.8%) were unknown). The DLBCL subtype was evaluated in 15 patients with RT; 14 had a non-germinal center B cell (GCB) type and 1 had a GCB type. The median Ki-67 index was 70% (interquartile range (IQR) 50–80%). Overall, 36 (75.0%) patients had received previous CLL-directed therapy, including chemoimmunotherapy (CIT; 25 patients) and targeted agents (32 patients) as well as previous allogeneic stem cell transplant (SCT) in 3 patients. Of those patients with previous targeted treatment, 24 had received previous BTK inhibitor treatment, 22 patients had previous treatment with BCL-2 inhibitors and 2 patients had received previous combined BTK + BCL-2 inhibitor therapy (Table 1). Twelve (25.0%) patients had treatment-naive CLL. Ten patients (20.8%) had received previous RT-directed therapy, including R-CHOP-like regimens and one case of previous ibrutinib treatment. A total of 38 (79.2%) patients had not received previous RT-directed therapy (Table 1).

**Table 1:** Baseline patient characteristics

Patient characteristics	
**All patients**	**48**
**Age (years)**	**48**
Median	67
IQR	60–74
Range	45–82
**Sex,** ***n*** **(%)**	**48**
Female	19 (39.6)
Male	29 (60.4)
**Time between CLL diagnosis and study registration (months)**	**48**
Median	79
IQR	49–136
**Time between RT diagnosis and study registration (months)**	**48**
Median	0.7
IQR	0.43–1.04
**Number of previous CLL-directed therapies**	**36**
Median	3
Range	1–6
**Patients with previous CLL-directed therapies,** ***n*** **(%)**	**36**
Chemo(immuno)therapy	25 (69.4)
SCT	3 (8.3)
BTK/BCL-2 inhibitors	32 (88.9)
BTK inhibitor	24 (66.7)
BCL-2 inhibitor	22 (61.1)
BTK + BCL-2 inhibitor	2 (5.6)
Other	9 (25.0)
**Binet stage,** ***n*** **(%)**	**48**
A	22 (45.8)
B	8 (16.7)
C	18 (37.5)
**Severe constitutional symptoms,** ***n*** **(%)**	**48**
No	28 (58.3)
Yes	20 (41.7)
**ECOG performance status,** ***n*** **(%)**	**48**
0	26 (54.2)
1	15 (31.3)
2	6 (12.5)
3	1 (2.1)
**CIRS total score**	**48**
Median	4
IQR	2–7
**CIRS total score,** ***n*** **(%)**	**48**
≤6	34 (70.8)
>6	14 (29.2)
**LDH (U** **l**^**−1**^**)**	**48**
Median	335
IQR	209–584
Patients with LDH > 250 U l^−1^, *n* (%)	31 (64.6)
**Cytogenetic subgroups hierarchical order (according to Döhner et al.**^46^**),** ***n*** **(%)**	**46**
Deletion 17p	10 (21.7)
Deletion 11q	4 (8.7)
Trisomy 12	5 (10.9)
No abnormalities	19 (41.3)
Deletion 13q	8 (17.4)
Missing	2 (4.2)
***TP53*** **mutation status,** ***n*** **(%)**	**45**
Unmutated	32 (71.1)
Mutated	13 (28.9)
Missing	*3 (6.3)*
***TP53*** **status,** ***n*** **(%)**	**46**
None	30 (65.2)
Deleted and/or mutated	16 (34.8)
Missing	2 (4.2)
**IGHV mutation status,** ***n*** **(%)**	**41**
Unmutated	29 (70.7)
Mutated	12 (29.3)
Missing	7 (14.6)
**Serum thymidine kinase (U** **l**^**−1**^**)**	**47**
Median	40.1
IQR	18.5–108.3
**Serum β**_**2**_**-microglobulin (mg** **l**^**−1**^**)**	**47**
Median	3.8
IQR	2.5–5.5
**Complex karyotype,** ***n*** **(%)**	**38**
<3 aberrations	22 (57.9)
≥3 aberrations	16 (42.1)
Missing	10 (20.8)
**CLL-IPI risk group,** ***n*** **(%)**	**39**
Low	3 (7.7)
Intermediate	11 (28.2)
High	10 (25.6)
Very high	15 (38.5)
Missing	9 (18.8)
**RT features,** ***n*** **(%)**	**48**
Previously untreated RT	38 (79.2)
Previously treated with RT-directed therapy	10 (20.8)
HL	2 (4.2)
DLBCL	46 (95.8)
Non-GCB	14 (29.2)
GCB	1 (2.1)
Unknown	33 (68.8)
Clonally unrelated	0 (0.0)
Clonally related	26 (54.2)
Unknown	22 (45.8)
**Ki-67 (%)**	**31**
Median	70
IQR	50–80

Cytogenetics, *TP53* and IGHV status and complex karyotype status were derived from peripheral blood and thus represent the CLL fraction.

**Table 2:** Adverse events

	Max CTC grade				57
Adverse event	1–2	3	4	5	Total
**Blood and lymphatic system disorders**	7 (12.3)	11 (19.3)	9 (15.8)	0 (0.0)	27 (47.4)
Anemia	3 (5.3)	8 (14.0)	0 (0.0)	0 (0.0)	11 (19.3)
Neutropenia	1 (1.8)	4 (7.0)	7 (12.3)	0 (0.0)	12 (21.1)
Thrombocytopenia	5 (8.8)	1 (1.8)	5 (8.8)	0 (0.0)	11 (19.3)
**Cardiac disorders**	4 (7.0)	1 (1.8)	0 (0.0)	0 (0.0)	5 (8.8)
**Ear and labyrinth disorders**	5 (8.8)	0 (0.0)	0 (0.0)	0 (0.0)	5 (8.8)
Vertigo	5 (8.8)	0 (0.0)	0 (0.0)	0 (0.0)	5 (8.8)
**Eye disorders**	7 (12.3)	0 (0.0)	0 (0.0)	0 (0.0)	7 (12.3)
**Gastrointestinal disorders**	26 (45.6)	6 (10.5)	0 (0.0)	0 (0.0)	32 (56.1)
Abdominal pain	5 (8.8)	0 (0.0)	0 (0.0)	0 (0.0)	5 (8.8)
Abdominal pain upper	5 (8.8)	0 (0.0)	0 (0.0)	0 (0.0)	5 (8.8)
Diarrhea	13 (22.8)	3 (5.3)	0 (0.0)	0 (0.0)	16 (28.1)
Nausea	10 (17.5)	0 (0.0)	0 (0.0)	0 (0.0)	10 (17.5)
**General disorders and administration site conditions**	28 (49.1)	2 (3.5)	0 (0.0)	0 (0.0)	30 (52.6)
Fatigue	5 (8.8)	0 (0.0)	0 (0.0)	0 (0.0)	5 (8.8)
Edema	5 (8.8)	0 (0.0)	0 (0.0)	0 (0.0)	5 (8.8)
Edema peripheral	10 (17.5)	0 (0.0)	0 (0.0)	0 (0.0)	10 (17.5)
Pyrexia	10 (17.5)	1 (1.8)	0 (0.0)	0 (0.0)	11 (19.3)
**Immune system disorders**	2 (3.5)	3 (5.3)	0 (0.0)	0 (0.0)	5 (8.8)
**Infections and infestations**	21 (36.8)	20 (35.1)	1 (1.8)	3 (5.3)	45 (78.9)
COVID-19	12 (21.1)	1 (1.8)	0 (0.0)	0 (0.0)	13 (22.8)
Infection	0 (0.0)	4 (7.0)	0 (0.0)	0 (0.0)	4 (7.0)
Nasopharyngitis	8 (14.0)	0 (0.0)	0 (0.0)	0 (0.0)	8 (14.0)
Oral herpes	4 (7.0)	0 (0.0)	0 (0.0)	0 (0.0)	4 (7.0)
Urinary tract infection	3 (5.3)	9 (15.8)	0 (0.0)	0 (0.0)	12 (21.1)
**Injury, poisoning and procedural complications**	9 (15.8)	1 (1.8)	0 (0.0)	0 (0.0)	10 (17.5)
Infusion related reaction	5 (8.8)	0 (0.0)	0 (0.0)	0 (0.0)	5 (8.8)
**Investigations**	13 (22.8)	3 (5.3)	3 (5.3)	0 (0.0)	19 (33.3)
Blood creatinine increased	6 (10.5)	0 (0.0)	0 (0.0)	0 (0.0)	6 (10.5)
**Metabolism and nutrition disorders**	7 (12.3)	10 (17.5)	0 (0.0)	0 (0.0)	17 (29.8)
Hypokalemia	7 (12.3)	2 (3.5)	0 (0.0)	0 (0.0)	9 (15.8)
**Musculoskeletal and connective tissue disorders**	17 (29.8)	4 (7.0)	0 (0.0)	0 (0.0)	21 (36.8)
Arthralgia	6 (10.5)	0 (0.0)	0 (0.0)	0 (0.0)	6 (10.5)
Back pain	5 (8.8)	1 (1.8)	0 (0.0)	0 (0.0)	6 (10.5)
**Nervous system disorders**	13 (22.8)	7 (12.3)	0 (0.0)	0 (0.0)	20 (35.1)
Dizziness	7 (12.3)	0 (0.0)	0 (0.0)	0 (0.0)	7 (12.3)
Headache	5 (8.8)	0 (0.0)	0 (0.0)	0 (0.0)	5 (8.8)
**Psychiatric disorders**	7 (12.3)	1 (1.8)	0 (0.0)	0 (0.0)	8 (14.0)
**Renal and urinary disorders**	6 (10.5)	7 (12.3)	0 (0.0)	0 (0.0)	13 (22.8)
**Reproductive system and breast disorders**	2 (3.5)	0 (0.0)	0 (0.0)	0 (0.0)	2 (3.5)
**Respiratory, thoracic and mediastinal disorders**	10 (17.5)	4 (7.0)	0 (0.0)	0 (0.0)	14 (24.6)
Cough	6 (10.5)	0 (0.0)	0 (0.0)	0 (0.0)	6 (10.5)
Epistaxis	4 (7.0)	1 (1.8)	0 (0.0)	0 (0.0)	5 (8.8)
**Skin and subcutaneous tissue disorders**	23 (40.4)	1 (1.8)	0 (0.0)	0 (0.0)	24 (42.1)
Eczema	4 (7.0)	0 (0.0)	0 (0.0)	0 (0.0)	4 (7.0)
Petechiae	6 (10.5)	0 (0.0)	0 (0.0)	0 (0.0)	6 (10.5)
Pruritus	6 (10.5)	0 (0.0)	0 (0.0)	0 (0.0)	6 (10.5)
Rash	10 (17.5)	1 (1.8)	0 (0.0)	0 (0.0)	11 (19.3)
**Surgical and medical procedures**	3 (5.3)	0 (0.0)	0 (0.0)	0 (0.0)	3 (5.3)
**Vascular disorders**	8 (14.0)	4 (7.0)	0 (0.0)	0 (0.0)	12 (21.1)
Hematoma	4 (7.0)	0 (0.0)	0 (0.0)	0 (0.0)	4 (7.0)
Hypotension	3 (5.3)	1 (1.8)	0 (0.0)	0 (0.0)	4 (7.0)

**Fig. 2:**
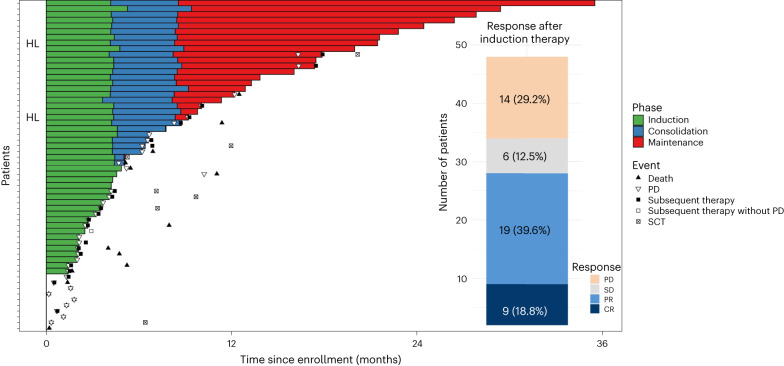
Response rates and duration of treatment. Swimmer plot depicts disease assessments and treatment phase and duration. Bar chart indicates response rates. CR, complete response; PR, partial response; SD, stable disease.

With a data cutoff of 2 May 2023, 19 patients were still under ongoing treatment (Fig. 2). Overall median observation time was 13.9 months (IQR 8.7–22.2) and median observation time for patients still alive was 12.0 months (IQR 8.4–22.1). The median number of treatment cycles of tislelizumab was 9 (IQR 4–23) and of zanubrutinib 11 (IQR 5–25).

### Efficacy end points

**Fig. 3:**
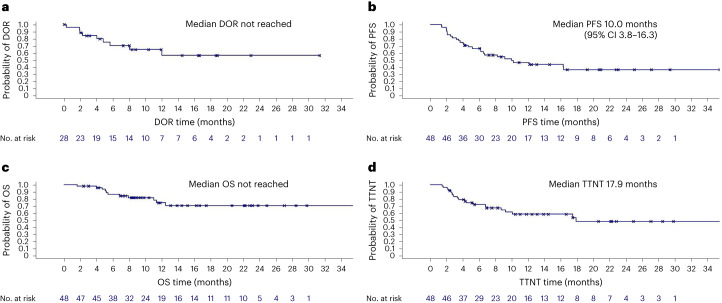
Kaplan–Meier analyses for secondary efficacy end points. **a**, DOR. **b**, PFS. **c**, Overall survival (OS). **d**, TTNT.

**Extended Data Fig. 1:**
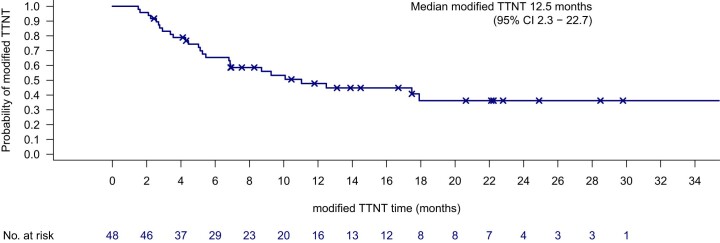
Modified time-to-next-treatment. Time-to-next-treatment defined as initiation of a next line of treatment or death, whichever occurred first.

Twenty-eight of 48 patients responded to induction therapy resulting in an ORR of 58.3% (95% CI 43.2–72.4), including 9 (18.8%) complete response and 19 (39.6%) partial response, meeting the study’s primary end point (*P* = 0.008) by rejecting the predefined null hypothesis of 40% (Fig. 1). Stable disease was reported in 6 (12.5%) patients and 14 (29.2%) patients had progressive disease. The ORR as assessed according to the refined Lugano criteria agreed with the ORR according to iwCLL criteria. The median DOR was not reached; the 6-month DOR rate was 70.6% (95% CI 51.0–90.2; Fig. 3a). The median PFS was 10 months (95% CI 3.8–16.3) with a 12-month rate of 46.9% (95% CI 29.4–64.5; Fig. 3b). The median overall survival was not reached (12-month overall survival rate 74.7%, 95% CI 58.4–91.0) (Fig. 3c). All deaths were associated with disease progression. The median TTNT, defined as time to initiation of a next line of treatment with censoring of deaths, was 17.9 months (12-month TTNT rate 58.5%, 95% CI 40.7–76.4)) and 12.5 months (12-month TTNT rate 50.2%, 95% CI 32.2–68.1) when defined as time to initiation of a next line of treatment or death, whatever occurred first (Fig. 3d and Extended Data Fig. 1). Three of 48 patients have not reached the end of consolidation after 12 cycles so far. The ORR in the remaining 45 patients was 46.7% (95% CI 31.7–62.1) with a complete response in 10 patients (22.2%), partial response in 11 patients (24.4%), stable disease in 3 patients (6.7%), progressive disease in 3 patients (6.7%) and missing data in 18 patients (40.0%, including 16 patients with discontinuation of therapy before reaching the end of consolidation).

Post-protocol treatment included chemoimmunotherapy in 21 cases (50.0%), BTK/BCL-2 inhibition in 7 (16.7%) cases and 8 (19.0%) allogeneic SCT (Extended Data Table 1). SCT was conducted as consolidation in two patients with partial response and as salvage treatment for five patients with stable disease or progressive disease (one missing response) (Fig. 2).

None of the assessed baseline clinical, serological or genomic features was significantly associated with response or non-response (Extended Data Table 2). In a univariate analysis, factors significantly associated with shorter PFS were the presence of severe constitutional symptoms, ECOG > 0, LDH, thymidine kinase and serum β_2_-microglobulin (>3.5 mg l^−1^). Shorter overall survival was associated with Binet C, age, severe constitutional symptoms, LDH, thymidine kinase and serum β_2_-microglobulin (>3.5 mg l^−1^). Shorter DOR was associated with presence of Binet C, LDH, *TP53* deletion and/or mutation, thymidine kinase and serum β_2_-microglobulin (Extended Data Tables 3–5).

**Extended Data Fig. 2:**
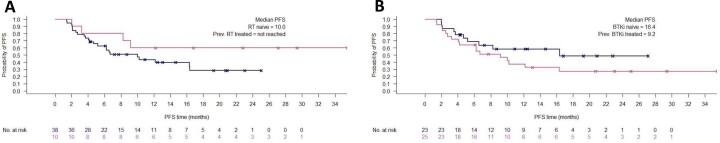
Survival analysis according to prior RT-directed treatment status and BTK inhibitor exposure. Progression-free survival (PFS) in patients with (red) and without (blue) prior RT-directed therapy (**A**) and patients with (red) and without (blue) prior BTK inhibitor exposure (**B**).

The ORR in patients without previous RT-directed therapy was 57.9% (95% CI 40.8–73.7) and 60.0% (95% CI 26.2–87.8) in patients with previous RT-directed therapy. Patients without previous RT-directed therapy had a 12-month PFS rate of 43.5% (95% CI 23.2–63.9) and patients with previous RT-directed therapy had a 12-month PFS rate of 60.0% (95% CI 19.9–100.0) (Extended Data Fig. 2a).

Patients without previous exposure to BTK inhibitors had an ORR of 69.6% (95% CI 47.1–86.8) and patients with previous BTK inhibitor therapy had an ORR of 48.0% (95% CI 27.8–68.7). The 12-month PFS rate in patients without previous BTK inhibitor therapy was 58.3% (95% CI 33.2–83.4) and 37.2% (95% CI 12.8–61.6) in patients with previous BTK inhibitor therapy (Extended Data Fig. 2b).

**Extended Data Fig. 3:**
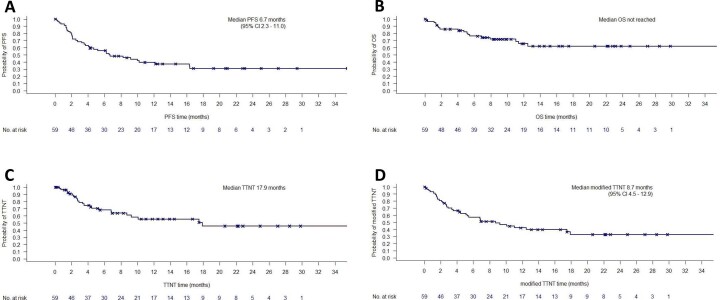
Post-hoc survival analyses of all study eligible patients. Analysis of all study eligible patients, including those 9 patients who did not complete two cycles of therapy due to primary progressive disease, adverse events, death or non-compliance. **a** Progression-free survival (PFS). **b** Overall survival (OS). **c** Time-to-next-treatment (TTNT). **d** Modified time-to-next-treatment (defined as next line of treatment or death, whichever occurred first).

A post hoc analysis of all 59 eligible patients, including those not receiving study treatment for at least two cycles (ITT population), demonstrated an ORR of 47.5% (95% CI 34.3–60.9); both patients with HL responded with a partial response. The median PFS of all eligible patients was 6.7 months (95% CI 2.3–11.0) with a 12-month rate of 39.5% (95% CI 23.8–55.3), median overall survival was not reached (12-month overall survival rate 65.7%, 95% CI 49.3–82.0) and median TTNT was 17.9 months (12-month TTNT rate 55.4%, 95% CI 38.0–72.7) (Extended Data Fig. 3 and Extended Data Table 6).

### Safety end points

For the safety analysis, all 57 included patients who had received at least one dose of any study medication were considered. A total of 56 (98.2%) patients experienced at least one grade ≥1 adverse event during the observation period. The most common adverse events of any grade occurring during the observation period were gastrointestinal disorders (56.1%), including diarrhea (28.1%) and nausea (17.5%), general disorders (52.6%), including pyrexia (19.3%), peripheral edema (17.5%), edema (8.8%) and fatigue (8.8%), blood and lymphatic system disorders (47.4%), including anemia (19.3%), neutropenia (21.1%) and thrombocytopenia (19.3%) and infections and infestations (78.9%), including COVID-19 (22.8%) and urinary tract infections (21.1%).

Cardiac toxicities, of interest in the context of BTK inhibitors, were uncommon, with one case each of angina pectoris (grade 3), cardiac failure (grade 2), cardiovascular disorder (grade 1), mitral valve insufficiency (grade 2) and sinus bradycardia (grade 1); no atrial fibrillation episodes were reported. Grade 1 to 3 hypertension was reported in three cases, of which two patients had a previous history of arterial hypertension. Hematoma was reported in five cases (grade 1 and 2) and one case of grade 3 cerebral hemorrhage occurred in a patient on prophylactic concomitating aspirin.

Potentially immune-related disorders, of interest in the context of checkpoint inhibitors, included two cases of thyroid disorders (hypothyroidism, grade 2), pyrexia (12 cases, grade 1–3) and increased liver values (five cases, one hyperbilirubinemia and four transaminitis, grade 1–4).

Overall, three grade 5 adverse events were reported in the safety population and all of them were related to fatal sepsis.

## Discussion

The improved understanding of the pathophysiology of CLL has led to the development of targeted agents that leverage distinct vulnerabilities and dependencies of malignant CLL cells. Targeted agents have thus demonstrated higher efficacy than chemotherapy in all risk groups of CLL^31,32^; however, the prevention and therapy RT remains one of the major clinical challenges in the management of CLL^2^. While recent studies have suggested multiple mechanisms contributing to transformation of CLL^33^, the standard of care for RT has largely remained unchanged for a few decades, as chemoimmunotherapies such as R-CHOP or DA-EPOCH have remained the most commonly used therapies outside of clinical studies, despite short responses, high toxicity and short overall survival of less than a year^4,34,35^.

Previously, several studies have explored the use of targeted agents in the context of RT. Covalent and non-covalent BTK inhibitors such as acalabrutinib and pirtobrutinib are very well tolerated in patients with RT; however, efficacy is limited owing to low ORRs with short durations^9,24^. Likewise, monotherapy with PD-1 inhibitors can induce responses that last very briefly when used as single agents^18,36^. Combination of targeted agents with R-CHOP and DA-EPOCH-R have also been clinically tested, with R-CHOP/DA-EPOCH-R plus venetoclax demonstrating high and durable responses, albeit with toxicity rates largely in line with previous reports on chemoimmunotherapy plus BCL-2 inhibitors in DLBCL^6,37^. Targeted combination therapies of RT have been explored with nivolumab plus ibrutinib in a monocentric study^38^ as well as a triple combination of atezolizumab, venetoclax and obinutuzumab in the MOLTO study^39^. These approaches have demonstrated encouraging efficacy with good tolerability.

To the best of our knowledge, the RT1 study is so far, one of the largest prospective phase 2 studies of a targeted treatment approach in RT. Patients with previously treated as well as untreated RT experienced response to combined checkpoint and BTK inhibition with tislelizumab and zanubrutinib, while experiencing little and manageable toxicity rates. The ORR of 58%, including a complete response rate of 19%, lasted for 6 months or more in over 70% of patients, with the median DOR not reached. While the 12-month PFS rate of 47% indicates that most patients eventually experience disease relapses, the 12-month overall survival rate of 75% is higher than historical reports on the expected overall survival of patients with RT^4,34,35^. Of note, most patients with disease progressions received subsequent chemoimmunotherapy with CHOP-like regimens and overall, eight patients underwent allogeneic SCT, indicating the general feasibility of these salvage strategies after PD-1 and BTK inhibition.

The regimen was generally very well tolerated, with a low number of immune-related adverse events, which have been previously observed with various checkpoint inhibitors^40^, as well as a low incidence of cardiovascular toxicities, as seen by the lack of atrial fibrillation events, previously associated with first-generation BTK inhibitors^41^.

A conceptually similar approach to the RT1 study was previously tested in a monocentric study using nivolumab plus ibrutinib; however, while the data were encouraging with response rates of 42%, the sample size was limited^38^. Moreover, owing to the relevant cardiovascular toxicity of ibrutinib, it is increasingly replaced by next-generation inhibitors such as acalabrutinib and zanubrutinib, which demonstrated less toxicity and also, in the case of zanubrutinib, higher efficacy^29^.

The data generated from this first analysis of the RT1 study have limitations. As this study is non-randomized, a direct comparison of the efficacy of tislelizumab plus zanubrutinib with the current standard of care of R-CHOP/EPOCH-R is not possible; however, the clinical outcomes observed in this study are consistently more favorable than what has been reported in retrospective analyses of RT^4,34,35^. The patient population enrolled in the RT1 study was relatively fit with half of the patients having an ECOG performance status of 0, albeit with a median CIRS score of 4; outside of clinical studies, the RT patient population is likely to be less fit owing to the aggressive nature of RT. The RT1 patient population did not include patients with non-response to a previous RT-directed therapy or more than one previous line of therapy. Therefore, the data cannot be directly extrapolated to patients with multiple previous treatments or with primary progressive RT.

While the study regimen is efficacious, the outcomes are still substantially poorer than what is commonly observed in non-transformed CLL^42–44^, demonstrating the need to further optimize the regimen. To interrogate determinants of response versus non-response to the study regimen, correlative studies are ongoing to delineate the genomic, transcriptomic and immune profiles, including measurement of PD-L1 expression, in patients treated in the RT1 study. Finally, to further increase the rate and DOR, the RT1 protocol is currently being amended to add the next-generation BCL-2 inhibitor sonrotoclax to tislelizumab plus zanubrutinib to increase efficacy by a triple-therapy approach.

In conclusion, combined checkpoint and BTK inhibition by tislelizumab plus zanubrutinib is an effective and well-tolerated treatment strategy for patients with RT. The response to therapy and overall survival rates at 1 year in the RT1 study are encouraging given the otherwise poor prognosis of RT.

## Methods

### Study design and participants

RT1 is an investigator-initiated, prospective, open-label, multicenter phase 2 study (NCT04271956) that enrolled patients from February 2020 to January 2023 at 12 investigative centers. Patients were recruited from ten sites in Germany (University Hospital of Cologne, University Hospital Kiel, University Hospital Essen, Otto-von-Guericke University Magdeburg, University Hospital Rostock, University Hospital Dresden, University Hospital Ulm, Munich Clinic Schwabing, Brüderkrankenhaus Paderborn and MVZ Dr Vehling-Kaiser Landshut), one site in Austria (Medical University of Vienna) and one site in Denmark (Rigshospitalet Copenhagen). Eligible patients aged ≥18 years had a diagnosis of CLL as defined by iwCLL criteria^3^ and a confirmed diagnosis of RT based on histopathological examination by an expert hematopathologist. Patients were allowed to have up to one previous line of RT-directed therapy. As further inclusion criteria adequate kidney (creatinine clearance ≥30 ml min^−1^) and liver function (total bilirubin ≤2×, AST/ALT ≤ 2.5× the institutional upper limit of normal value, unless directly attributable to the patient’s CLL/RT or to Gilbert’s syndrome) were required as well as negative serological testing for hepatitis B virus (patients positive for anti-HBc were included if PCR for hepatitis B virus DNA was negative and hepatitis B virus DNA PCR was performed every two months until 2 months after last dose of zanubrutinib), hepatitis C and HIV. Patients with an ECOG performance status of 0–2 or 3 (if due to underlying CLL/RT) were eligible. Eligible patients had a life expectancy equal to or greater than 3 months and were able to provide informed written consent. Exclusion criteria were primary progressive disease (non-response to previous RT-directed therapy, as it was initially not clear how fast the study regimen could induce remissions in patients with RT), patients with more than one previous line of RT therapy and allogeneic SCT within the last 100 days or signs of active graft-versus-host disease. Further exclusion criteria were confirmed progressive multifocal leukoencephalopathy, an uncontrolled autoimmune condition, malignancies other than CLL requiring system therapy, active infections requiring systemic treatment, organ system impairments with a CIRS score of 4 or higher, excluding eyes, ears, nose, throat or larynx organ system, requirement of treatment with strong CYP3A4 inhibitors or inducers, requirement of treatment with phenprocoumon or other vitamin K antagonists, use of other investigational agents, known hypersensitivity to tislelizumab or zanubrutinib, pregnant women and nursing mothers, live vaccination within 28 days previous to enrollment, legal incapacity, prisoners or institutionalized persons and persons in dependence to the sponsor or investigator.

This study was conducted in accordance with the Declaration of Helsinki and the International Conference on Harmonization guidelines for Good Clinical Practice. All patients provided written informed consent. The study protocol and relevant documents were approved by an independent institutional review board or ethics committee at each investigative center. The study was reviewed and approved by all responsible ethics committees (Ethics Committee of the Medical Faculty of the Christian Albrechts University in Kiel; Ethics Committee of the Medical Faculty of the University of Duisburg-Essen; Ethics Committee of the University of Cologne (Central Ethics Committee in Germany); OVGU Ethics Committee at the Medical Faculty; Ethics Committee of the Medical Faculty of the University of Rostock; Ethics Committee of the Medical Faculty of the TU Dresden; Ethics Committee of the University of Ulm; Ethics Committee of the Medical Faculty of the LMU Munich; Ethics committee of the Westfalen-Lippe Medical Association and the Medical Faculty of the Westphalian Wilhelms University of Münster; Ethics Committee of the Bavarian State Medical Association; Ethics Committee of the Medical University of Vienna; and the National Videnskabsetisk Komité, Copenhagen). No data safety monitoring board was implemented in the RT1 protocol. The study protocol and statistical analysis plan are provided in the Supplementary Information.

### Procedures

Each treatment cycle consisted of 21 days. Patients received tislelizumab intravenously at a fixed dose of 200 mg on day 1 of each cycle. Previous to the first infusion of tislelizumab, a pre-medication with an antihistamine and paracetamol was permitted, in addition to oral or intravenous glucocorticoids if considered indicated by the investigator. The first infusion was administered over 60 min and subsequent infusions over 30 min. Zanubrutinib was administered orally at a fixed dose of 160 mg twice daily from day 1 onwards. Dose modifications or interruptions were permitted for management of adverse events. Before the induction phase, a pre-phase therapy with steroids, vincristine (up to 2 mg intravenously) or cyclophosphamide (up to 200 mg^2^ for a maximum of 3 d) was permitted in cases with urgent need for treatment. The induction phase consisted of six treatment cycles, followed by a consolidation phase of six further cycles. Patients with response or stable disease after 12 cycles were allowed to proceed with maintenance treatment with tislelizumab plus zanubrutinib at the investigator’s discretion.

### Outcomes

Per protocol, the primary end point was the ORR at the interim staging after end of induction therapy (after six cycles), for patients who received at least two cycles of study treatment, including at least one administered dose in cycle three, who comprised the full analysis set (FAS; see below). Response was assessed according to the refined Lugano criteria based on positron emission tomography–computed tomography or, if not available, based on computed tomography scans^45^. Secondary end points included ORR after the end of induction therapy (after six cycles) according to iwCLL criteria and ORR after consolidation therapy (12 cycles), DOR (for patients responding to induction therapy and defined as the time from enrollment to first assessment of response until disease progression or death from any cause), PFS (defined as the time from enrollment until disease progression or death from any cause), overall survival (defined as the time from enrollment until death from any cause), TTNT (defined as the time from enrollment until initiation of next treatment for CLL/RT) and safety parameters, including type, frequency and severity of adverse events.

Post hoc exploratory analyses included the assessment of ORR after six cycles and time-to-event analyses for all enrolled patients of the ITT population, the assessment of a modified TTNT (defined as the time from enrollment until initiation of next treatment for CLL/RT or death from any cause), univariate analyses of potential prognostic factors for ORR after six cycles, overall survival, PFS, DOR and the assessment of ORR and PFS, comparing of the RT and BTK-naive patients to previously treated patients.

Adverse events were graded according to National Cancer Institute Common Terminology Criteria for Adverse Events v.5.0. An interim safety analysis was conducted by the principal investigator, coordinating physician, statistician and safety management team of the German CLL Study Group (GCLLSG), after the first six patients had been treated for three cycles. Recruitment was only continued if no safety concerns were raised by the interim safety review.

### Statistical analysis

The protocol defined two patient populations for the statistical analyses. For the safety analysis, all patients who received at least one dose of study treatment were considered as the safety population. For the efficacy analysis, all patients who received at least two cycles of induction therapy, including at least one administered dose in cycle three, were considered as FAS; the FAS was used for the analysis of all study end points, apart from safety. Given the experimental nature of the study regimen, this FAS definition was chosen to ensure reliable data acquisition on the actual efficacy of the regimen and to reduce interactions— for example, due to comorbidities or non-adherence to study treatment — which was anticipated to be a possible confounder given the heterogeneous clinical presentation of RT. To account for selection bias possibly introduced by this approach, a post hoc analysis was conducted in all patients enrolled in the study (ITT population).

The primary end point ORR at the end of induction therapy was used to determine the sample size of the study. The null hypothesis was ORR ≤ 0.40 with the alternative hypothesis of ORR > 0.40. The type I error was set to α = 2.5% and the type II error should not exceed β = 20% to achieve a power of at least (1 − β) = 80%. Based on these parameters, a one-sided one-sample binomial test with an overall significance level of 2.5% provided the sample size of *n* = 48, to achieve a statistical significance with a power of 80%. The 95% CIs for the primary end point and secondary or exploratory response end points were calculated according to the Clopper–Pearson method and the Kaplan–Meier method was used for the time-to-event analyses of the secondary or exploratory end points. Univariate analyses of potential prognostic factors were performed for ORR after six cycles using logistic regression modeling and for overall survival, PFS and DOR using Cox proportional hazards regression modeling, each without adjustment for multiple testing. Statistical analyses were performed with EAST v.5, SPSS v.28 and R v.4.2.1. This study is registered with ClinicalTrials.gov (NCT04271956).

### Reporting summary

Further information on research design is available in the Nature Portfolio Reporting Summary linked to this article.

## Online content

Any methods, additional references, Nature Portfolio reporting summaries, source data, extended data, supplementary information, acknowledgements, peer review information; details of author contributions and competing interests; and statements of data and code availability are available at 10.1038/s41591-023-02722-9.

### Supplementary information

Supplementary Information (1) CONSORT checklist, (2) study protocol and (3) statistical analysis plan.

Reporting Summary

## Figures and Tables

**Extended Data Table 1:**
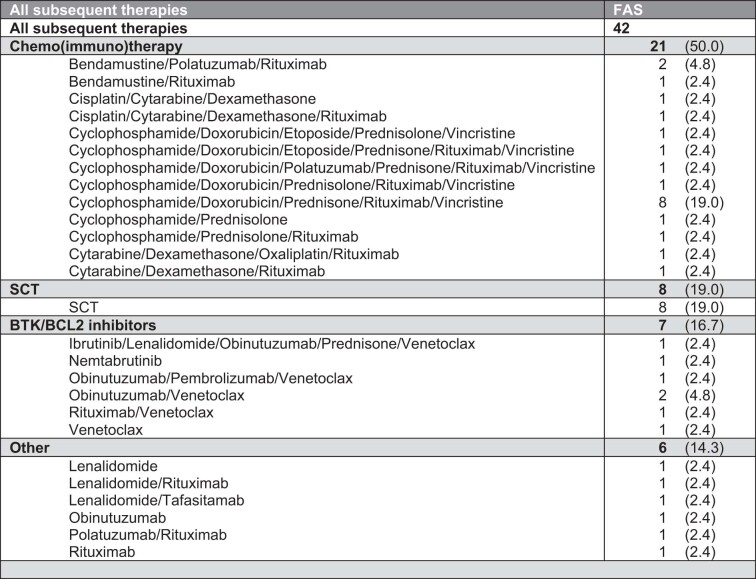
Post-protocol therapies (full analysis set)

NE, not evaluable.

**Extended Data Table 2:**
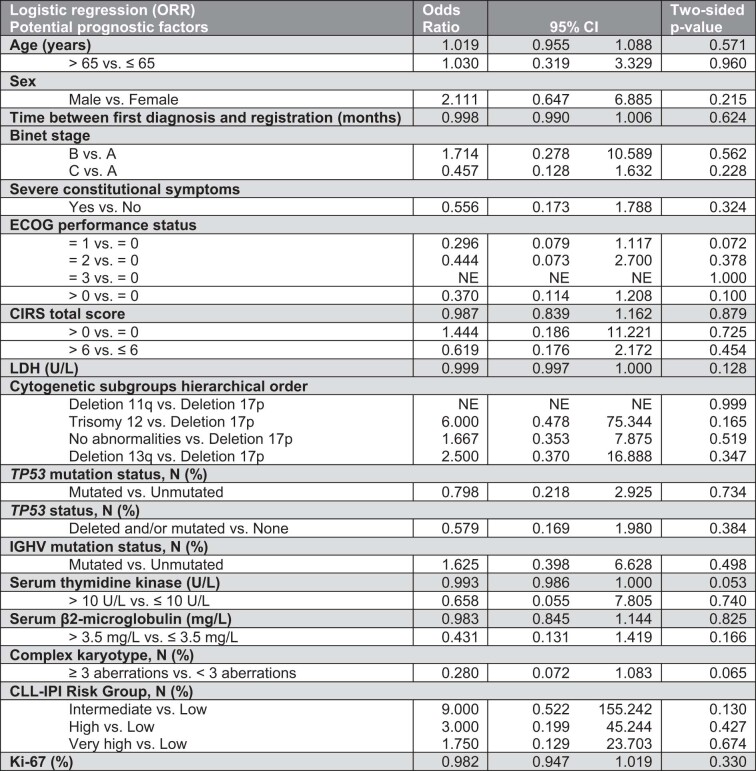
Univariate logistic regression analysis of baseline features for overall response rate after 6 cycles

Logistic regression analysis unadjusted for multiple testing.

**Extended Data Table 3:**
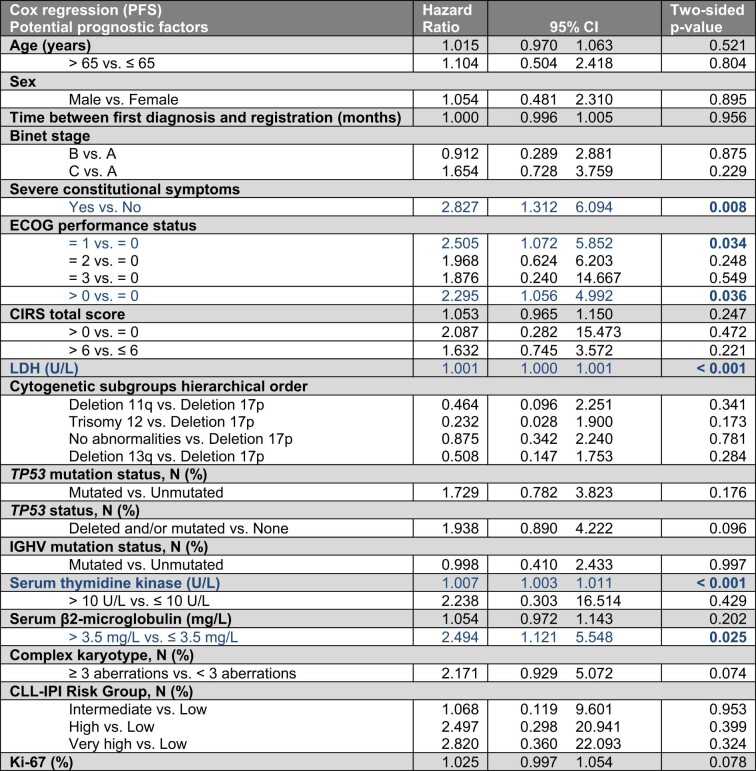
Univariate Cox regression analysis for progression-free survival

Univariate Cox regression analysis unadjusted for multiple comparisons. P-values < 0.05 are highlighted in bold.

**Extended Data Table 4:**
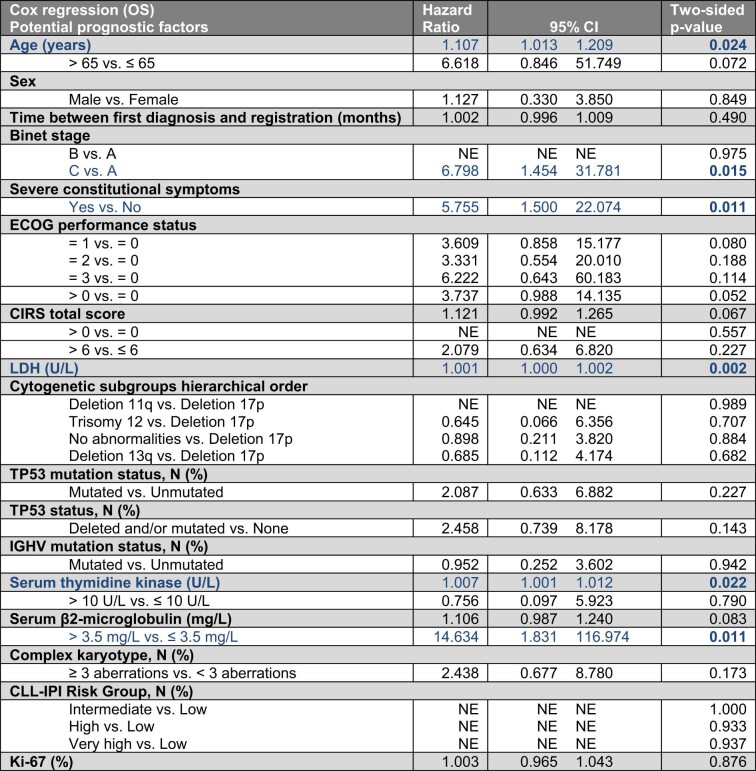
Univariate Cox regression analysis for overall survival

Univariate Cox regression analysis unadjusted for multiple comparisons. P-values < 0.05 are highlighted in bold.

**Extended Data Table 5:**
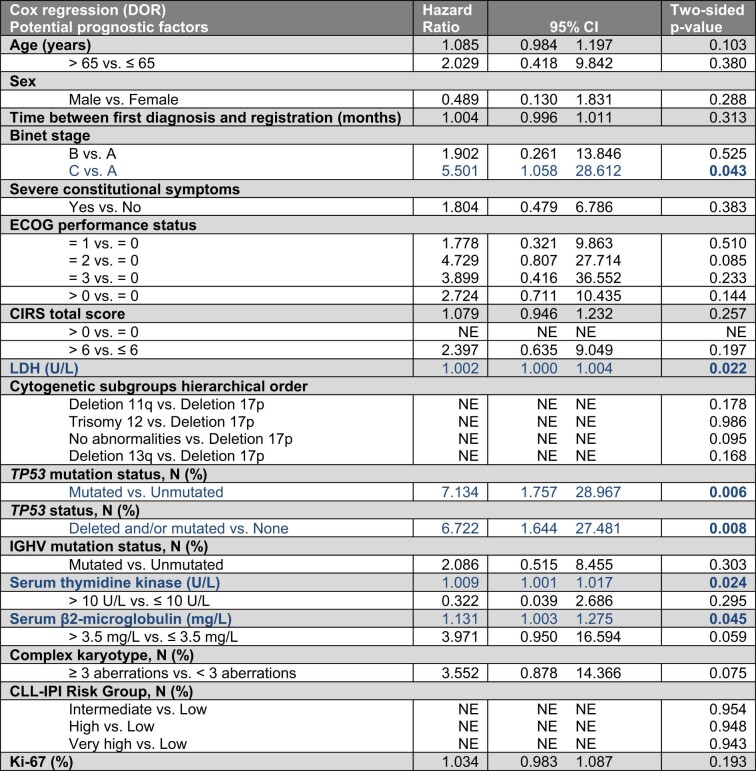
Univariate Cox regression analysis for duration of response

Univariate Cox regression analysis unadjusted for multiple comparisons. P-values < 0.05 are highlighted in bold.

**Extended Data Table 6:**
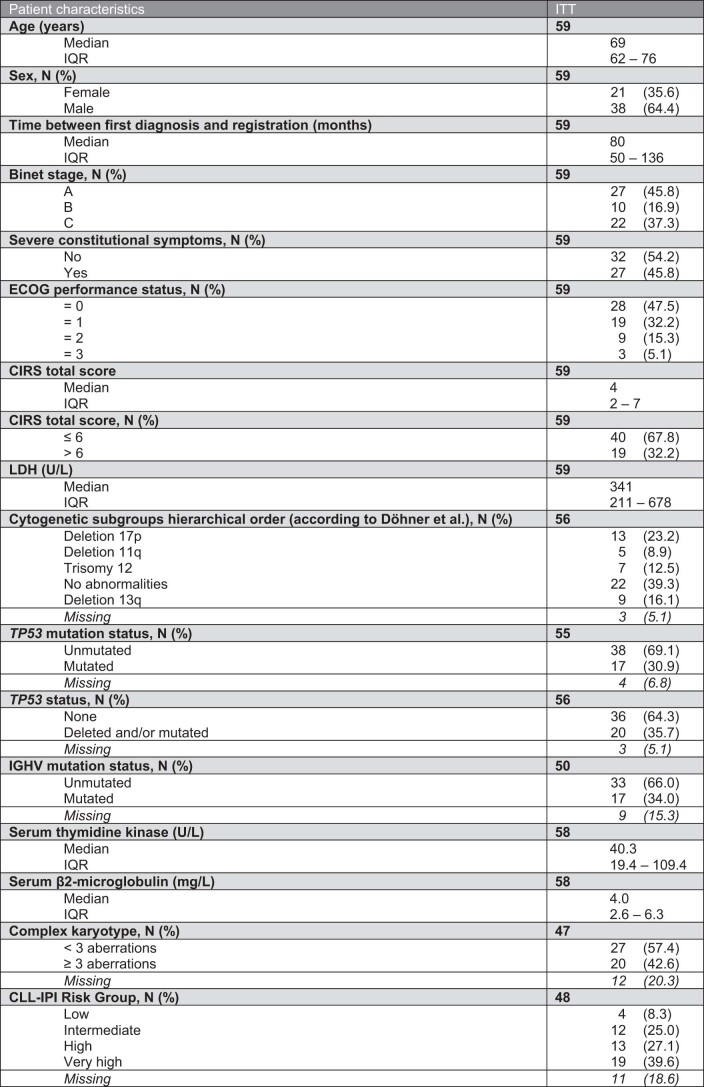
Patient characteristics (all study eligible patients)

## Data Availability

Access to individual patient-level data can be requested after publication via the corresponding authors (othman.al-sawaf@uk-koeln.de and barbara.eichhorst@uk-koeln.de), who will facilitate a central review by the GCLLSG within 6 months. The data will be released to such requesters with necessary agreements to enforce terms such as security, patient privacy and consent of specified data use, consistent with evolving, applicable data protection laws.
