# Supplementary material for: Tislelizumab plus zanubrutinib for Richter transformation: the phase 2 RT1 trial
Source: Nat Med. 2023 Dec 9;30(1):240–8. doi: 10.1038/s41591-023-02722-9 (PMC10803258; doi:10.1038/s41591-023-02722-9)
Supplement: Supplementary file 1 — (1) CONSORT checklist, (2) study protocol and (3) statistical analysis plan. [file 41591_2023_2722_MOESM1_ESM.pdf]

nature medicine

Article https://doi.org/10.1038/s41591-023-02722-9

Tislelizumab plus zanubrutinib for Richter transformation: the phase 2 RT1 trial

In the format provided by the authors and unedited

SUPPLEMENTARY MATERIAL

CONSORT checklist…….……..…….…………..……..……..……..…….……….1

RT1 study protocol…….……..……..……..……..……..……..……..…………...3

Statistical analysis plan…….………..………………..……..……..………108

CONSORT 2010 checklist of information to include when reporting a randomised trial*

Section/Topic	Item No	Checklist item	Reported on page No
**Title and abstract**			
	1a	Identification as a randomised trial in the title	Not applicable
	1b	Structured summary of trial design, methods, results, and conclusions (for specific guidance see CONSORT for abstracts)	1
**Introduction**			
Background and objectives	2a	Scientific background and explanation of rationale	2-3
	2b	Specific objectives or hypotheses	4
**Methods**			
Trial design	3a	Description of trial design (such as parallel, factorial) including allocation ratio	11
	3b	Important changes to methods after trial commencement (such as eligibility criteria), with reasons	Not applicable
Participants	4a	Eligibility criteria for participants	12
	4b	Settings and locations where the data were collected	11
Interventions	5	The interventions for each group with sufficient details to allow replication, including how and when they were actually administered	13
Outcomes	6a	Completely defined pre-specified primary and secondary outcome measures, including how and when they were assessed	14-15
	6b	Any changes to trial outcomes after the trial commenced, with reasons	Not applicable
Sample size	7a	How sample size was determined	15
	7b	When applicable, explanation of any interim analyses and stopping guidelines	Not applicable
Randomisation:			
Sequence generation	8a	Method used to generate the random allocation sequence	Not applicable
	8b	Type of randomisation; details of any restriction (such as blocking and block size)	Not applicable
Allocation concealment mechanism	9	Mechanism used to implement the random allocation sequence (such as sequentially numbered containers), describing any steps taken to conceal the sequence until interventions were assigned	Not applicable
Implementation	10	Who generated the random allocation sequence, who enrolled participants, and who assigned participants to interventions	Not applicable
Blinding	11a	If done, who was blinded after assignment to interventions (for example, participants, care providers, those	Not applicable

		assessing outcomes) and how	
	11b	If relevant, description of the similarity of interventions	Not applicable
Statistical methods	12a	Statistical methods used to compare groups for primary and secondary outcomes	15-16
	12b	Methods for additional analyses, such as subgroup analyses and adjusted analyses	16
**Results**			
Participant flow (a diagram is strongly recommended)	13a	For each group, the numbers of participants who were randomly assigned, received intended treatment, and were analysed for the primary outcome	4
	13b	For each group, losses and exclusions after randomisation, together with reasons	4
Recruitment	14a	Dates defining the periods of recruitment and follow-up	4
	14b	Why the trial ended or was stopped	Not applicable
Baseline data	15	A table showing baseline demographic and clinical characteristics for each group	19-21
Numbers analysed	16	For each group, number of participants (denominator) included in each analysis and whether the analysis was by original assigned groups	4-8
Outcomes and estimation	17a	For each primary and secondary outcome, results for each group, and the estimated effect size and its precision (such as 95% confidence interval)	4-7
	17b	For binary outcomes, presentation of both absolute and relative effect sizes is recommended	Not applicable
Ancillary analyses	18	Results of any other analyses performed, including subgroup analyses and adjusted analyses, distinguishing pre-specified from exploratory	7
Harms	19	All important harms or unintended effects in each group (for specific guidance see CONSORT for harms)	
**Discussion**			
Limitations	20	Trial limitations, addressing sources of potential bias, imprecision, and, if relevant, multiplicity of analyses	10-11
Generalisability	21	Generalisability (external validity, applicability) of the trial findings	10
Interpretation	22	Interpretation consistent with results, balancing benefits and harms, and considering other relevant evidence	8-10
**Other information**			
Registration	23	Registration number and name of trial registry	11
Protocol	24	Where the full trial protocol can be accessed, if available	
Funding	25	Sources of funding and other support (such as supply of drugs), role of funders	1

*We strongly recommend reading this statement in conjunction with the CONSORT 2010 Explanation and Elaboration for important clarifications on all the items. If relevant, we also recommend reading CONSORT extensions for cluster randomised trials, non-inferiority and equivalence trials, non-pharmacological treatments, herbal interventions, and pragmatic trials. Additional extensions are forthcoming: for those and for up to date references relevant to this checklist, see www.consort-statement.org.

Universität zu Köln

TRIAL PROTOCOL

A PROSPECTIVE, OPEN-LABEL, MULTICENTRE **PHASE-II-TRIAL** TO EVALUATE THE EFFICACY AND SAFETY OF **ZANUBRUTINIB (BGB-3111)**, A BTK INHIBITOR, PLUS **TISLELIZUMAB (BGB-A317)**, A PD-1 INHIBITOR, FOR TREATMENT OF PATIENTS WITH **RICHTER TRANSFORMATION**

(CLL-RT1-TRIAL OF THE GCLLSG)

SPONSOR:	UNIVERSITY OF COLOGNE<br>ALBERTUS-MAGNUS-PLATZ<br>50923 COLOGNE, GERMANY
GLOBAL PRINCIPAL INVESTIGATOR:	PROF DR. MED. BARBARA EICHHORST<br>COLOGNE UNIVERSITY HOSPITAL<br>KERPENER STR. 62<br>50937 COLOGNE, GERMANY
TRIAL PROTOCOL CODE:	**CLL-RT1**
EUDRACT-NUMBER:	2018-002492-17
SPONSOR-NUMBER:	UNI-KOELN-3590
PROTOCOL VERSION:	JANUARY 11, 2023, 2021; VERSION 4.0
PROTOCOL COMMITTEE:	O. Al-Sawaf, Cologne<br>S. Robrecht, Cologne<br>B. Eichhorst, Cologne<br>A.-M. Fink, Cologne<br>K. Fischer, Cologne<br>M. Hallek, Cologne<br>U. Jäger, Vienna<br>K.-A. Kreuzer, Cologne<br>C. Niemann, Kopenhagen<br>C. Pallasch, Cologne<br>S. Stilgenbauer, Ulm<br>J. Stumpf, Cologne<br>C.-M. Wendtner, Munich

The information in this trial protocol is strictly confidential. It is for the use of the sponsor, investigator, trial personnel, ethics committee, the authorities, and trial subjects only. This trial protocol may not be passed on to third parties without the express agreement of the sponsor or the Global Principal Investigator (GPI, “Leiter der klinischen Prüfung (LKP)”)

II. Summary of Changes

Section/ page	Summary of correction	Previous text	Revised text
**Protocol Amendment 3**			
III / Page 13<br><br>14.2/ Page 95<br><br>14.3. / Page 96	Clarification provided on definition of Full analysis set (FAS)		Several paragraphs were adapted due to clarification on the FAS: The FAS comprises of all enrolled patients who received at least one dose of study medication in the third induction cycle (efficacy population).
III / Page 15	Updated study timeline	Start of recruitment Q1/2020<br>Expected end of recruitment Q4/2022<br>End of study Q4/2023	Start of recruitment Q1/2020<br>End of recruitment Q1/2023<br>Expected End of study Q1/2024
3.2.1 / Page 32	Update of paragraph of benefit-Risk Assessment regarding Covid-19 pandemic		Adapted paragraph ("Benefit-Risk Assessment regarding Covid-19 pandemic") was added.
4.7 / Page 35	Update on project management members	Laura Miesen (laura.miesen@uk-koeln.de, 0221-478-42564), Dr. Emily Holmes (emily.holmes@uk-koeln.de, 0221-478-96118) Study Office of the German CLL Study Group, Department I of Internal Medicine, Cologne University Hospital, Kerpener Str. 62, 50937 Cologne, Germany.	Vanessa Beste (vanessa.beste@uk-koeln.de, 0221-478-96123), Dr. Laura Miesen (laura.miesen@uk-koeln.de, 0221-478-42564), Dr. Emily Holmes (emily.holmes@uk-koeln.de, 0221-478-96118) Study Office of the German CLL Study Group, Department I of Internal Medicine, Cologne University Hospital, Kerpener Str. 62, 50937 Cologne, Germany.
7.3.1 / Page 43	Updated study timeline	Start of recruitment, i.e. first patient/first visit (FPFV): Q1/2020<br>Expected end of recruitment, i.e. last patient/first visit (LPFV): Q4/2022<br>Expected end of study, i.e. last patient/last visit (LPLV): Q4/2023<br>Final study report: Q4/2024	Start of recruitment, i.e. first patient/first visit (FPFV): Q1/2020<br>End of recruitment, i.e. last patient/first visit (LPFV): Q1/2023<br>Expected end of study, i.e. last patient/last visit (LPLV): Q1/2024<br>Final study report: Q1/2025
8.8.3. / Page 73	Potential risk with Zanubrutinib	Old chapter contains warnings for bleeding and tumor lysis syndrome	Amended chapter contains new information according to SmPC as of December 2022 including bleeding events, infec-

			tions, atrial fibrillation, secondary malignancies and tumor lysis syndrome
10.1. / Page 81	Updated Table 3		Clarifying updates on reporting period and documentation are added to the table
10.2.1. / Page 82	Correction of discrepancy with Chapter 10.2.4.	Disease progression should not be recorded as an adverse event or serious adverse event term unless it results in death within the SAE reporting period.	Disease progression should not be recorded as an adverse event or serious adverse event term.
14.5.2.2 / Page 98	Update on paragraph regarding duration of response	In the first case it will be calculated for patients with CR or PR, in the second case for patients with CR, CRi or PR.	In the first case it will be calculated for patients with CR or PR as EOIT response according to the definition of the primary endpoint, in the second case for patients with CR, CRi or PR as EOIT response.
14.6. / Page 98/99	Update on paragraph on Safety analysis	Safety parameters include treatment exposure, adverse events (including adverse events of special interest) and death cases. Analysis of safety parameters will be performed on the SP unless stated otherwise.<br><br>First, all cases of AEs will be reported in a case analysis including the number of events classified as being serious and related to study drugs. Specifications of AEs leading to death or early treatment discontinuation will also be summarized.	Safety parameters include treatment exposure, adverse events (including adverse events of special interest and adverse events of particular interest) and death cases. Analysis of safety parameters will be performed on the SP unless stated otherwise.<br><br>First, all cases of AEs will be reported in a case analysis including the number of events classified as being serious and related to study drugs (according to the investigator). Specifications of AEs leading to death or early treatment discontinuation will also be summarized.
17. / Page 103	Deletion of Appendix	A. Central laboratory assessment<br>B. G8 geriatric assessment<br>C. Remote SDV Plan	A. Central laboratory assessment<br>B. G8 geriatric assessment

III. Synopsis

Sponsor:	University of Cologne<br>Albertus-Magnus-Platz, 50923 Cologne, Germany<br>Represented by:<br>Prof. Dr. med. Barbara Eichhorst (GPI, LKP)<br>Department I of Internal Medicine, Cologne University Hospital<br>Kerpener Strasse 62, 50937 Cologne, Germany
Global Principal Investigator and medical contact of the sponsor:	Prof. Dr. med. Barbara Eichhorst<br>Department I of Internal Medicine, Cologne University Hospital<br>Kerpener Strasse 62, 50937 Cologne, Germany
Coordinating Physician	Dr. med. Othman Al-Sawaf<br>Department I of Internal Medicine, Cologne University Hospital<br>Kerpener Strasse 62, 50937 Cologne, Germany
Title of the clinical trial:	A prospective, open-label, multicenter phase-II trial to evaluate the efficacy and safety of zanubrutinib (BGB-3111), a BTK inhibitor, plus tislelizumab (BGB-A317), a PD1 inhibitor, for treatment of patients with Richter Transformation (CLL-RT1-trial of the GCLLSG)
Indication:	Patients with previously untreated Richter Transformation or patients who responded to up to one prior line of RT therapy
Phase:	Phase-II clinical trial
Type of trial, trial design, methodology:	Prospective, multicenter, phase-II trial, single-arm, open-label
Number of patients:	Approximately 48 eligible patients
Trial objectives:	The primary objective of the study is to evaluate the efficacy of a combinational therapy with tislelizumab and zanubrutinib in CLL patients with Richter transformation to DLBCL.<br>The secondary objective is to evaluate the safety of combinational therapy with tislelizumab and zanubrutinib in CLL patients with Richter transformation to DLBCL.
Rationale:	Richter syndrome (RS) or Richter transformation (RT) describes the rapid development of a histologically confirmed aggressive lymphoma, in most cases a diffuse large B cell lymphoma (DLBCL), in patients with CLL. The incidence rates of RT among CLL patients range from 2 to 10% [1]. RT can occur at any time during the course of CLL. Risk factors for development of RT include intrinsic biological features like TP53 mutations or 17p

deletions as well as therapy-related factors as exposure to purine analogues like fludarabine [2]. However, up to one third of patients with RT are treatment naïve CLL patients [3].

RT patients have a very poor prognosis with a median OS of 6-8 months. There is no established standard of care for RT and most patients are treated comparably to de-novo DLBCL patients with chemoimmunotherapies like R-CHOP or R-DHAP. Given the poor prognosis, fit patients are considered for allogenic transplantation once they respond to therapy. However, as CLL is a disease of the elderly with a median age of 72 years, most patients with RT are not fit enough to undergo allogenic transplantation.

The advent of a variety of novel antibodies and targeted drugs allows for new therapeutic approaches to address the unmet clinically need for a better care for RT patients.

Zanubrutinib (BGB-3111) is an orally bioavailable selective, irreversible inhibitor of Bruton's tyrosine kinase (BTK) that is currently developed in a variety of B-cell malignancies, including CLL and DLBCL. BTK is a well-established target for CLL treatment, as its inhibition by currently licensed agents like ibrutinib disrupts the BCR-dependent survival and proliferation of CLL cells. Pleiotropic effects of ibrutinib lead to distinct toxicities, particularly bleeding events and arrhythmia. Zanubrutinib is suggested to be more selective than ibrutinib and have less off target effects on other kinases like EGFR, JAK3 or ITK. Preclinical as well as early clinical data indicate that zanubrutinib has less side effects and a more favorable pharmacokinetic and pharmacodynamic profile [4].

Tislelizumab (BGB-A317) is a humanized IgG4 variant monoclonal antibody with no Fc gamma receptor binding that targets the programmed cell death-1 (PD-1) receptor. Expression of PD-1 is a mechanism by which malignant cells evade the immune system response. By blocking the interaction between PD-1 and its ligands, T-cells are allowed to recognize and kill tumor cells. So far, tislelizumab has shown clinical activity in a variety of tumors and is currently being tested in solid as well as hematological malignancies. A recent phase Ib trial has shown a manageable toxicity profile of the combination of zanubrutinib and tislelizumab in different b-cell malignancies [5].

Given that high PD-1 expression has been observed in patients with lymphoid malignancies, checkpoint inhibitors are promising candidates for treatment of RT. Previous data have shown that effective eradication of DLBCL cells in the bone marrow of RT patients can be achieved with single-agent PD-1 inhibitors [6]. However, persistence of CLL infiltration was observed as well, which suggests that a combinational approach might be indicated for effective treatment.

Currently, two trials are testing combinational approaches with nivolumab, a PD-1 inhibitor, plus ibrutinib and early interim analyses showed good response rates in pre-treated patients with RT [7, 8]. Moreover, single agent BTK inhibition has shown activity in RT [9-11]. Taken together, preclinical as well as early clinical data provide a good rationale to investigate on a combination of PD-1 inhibition plus BTK inhibition in

previously untreated patients with RT.

This prospective phase-II-trial will investigate a combinational regime of the PD-1 inhibitor tislelizumab and the BTK inhibitor zanubrutinib. The treatment schedule consists of 6 cycles of induction therapy (21-day cycles) during which tislelizumab will be administered once at a fixed dose, followed by 6 additional cycles of tislelizumab consolidation therapy. Zanubrutinib will be given two times daily (BID) from day 1 of cycle 1. Patients who show response to therapy after 12 cycles of therapy will continue until disease progression or unacceptable toxicities.

Study end points:

Primary endpoint:

Overall response rate (ORR) after induction therapy (i.e. 6 cycles) according to the refined Lugano Classification (Cheson et al, 2016) [12].

- Complete response (CR)
- Partial response (PR)

Secondary endpoints:

- ORR after induction therapy (i.e. 6 cycles) according to IWCLL criteria (Hallek et al, 2018)
- ORR after consolidation therapy (i.e. 12 cycles)
- Duration of response
- Progression-free survival (PFS)
- Overall survival (OS)
- Time to next treatment (TTNT)
- Proportion of patients receiving SCT for consolidation
- Exploratory endpoints: Evaluation of relationship between various baseline markers, including PD-1/PD-L1 expression and mutational load, and clinical outcome parameters
- Safety parameters: type, frequency, severity of adverse events (AEs), and their relationship to study treatment

Criteria for evaluation:

Efficacy

- FDG-PET-CT for confirmation of CR after induction
- Computed tomography (CT) scans at screening and for each staging
- Bone marrow aspirate/biopsy at screening and for confirmation of CR
- Complete blood count (CBC)
- Peripheral blood samples for immunophenotyping for confirmation of CLL diagnosis, serum parameters (Beta-2-microglobuline and Serum-Thymidine-Kinase), genetic evaluation
- Assessment of constitutional symptoms
- Survival status
- Survey of start and type of next treatment for CLL

Safety:

- Clinical laboratory evaluations
- ECOG Performance Status
- Assessment of comorbidity burden with CIRS-Score
- Concomitant medications
- AEs by NCI CTCAE Version 5.0
- HBV-DNA PCR every two months in patients with positive anti-HBc (irrespective of HBsAg) at screening
- pregnancy test within 7 days before start of treatment for all women of childbearing potential

Target Population:

Patients must meet the following criteria:

Inclusion Criteria

1. Confirmed diagnosis of CLL according to iwCLL criteria (Hallek et al, 2018) [13]
2. Confirmed histopathological diagnosis of RT (diffuse large B-cell lymphoma or Hodgkin's lymphoma [Hodgkin's lymphoma only when not eligible for more intensive treatment])
3. Previously untreated RT or patients with objective response or non-tolerance to first-line RT treatment

4. Creatinine clearance ≥30ml/min calculated according to the modified formula of Cockcroft and Gault or directly measured with 24hr urine collection or an equivalent method.

5. Adequate liver function as indicated by a total bilirubin≤ 2 x, AST/ALT ≤ 2.5 x the institutional ULN value, unless directly attributable to the patient's CLL/RT or to Gilbert's Syndrome, in which case a max. total bilirubin ≤ 4 x and AST/ALT ≤ 5 x the institutional ULN value are required.[1]

6. Negative serological testing for hepatitis B (HBsAg negative and anti-HBc negative; patients positive for anti-HBc may be included if PCR for HBV DNA is negative and HBV-DNA PCR is performed every two months until 2 months after last dose of zanubrutinib), negative testing for hepatitis-C RNA and negative HIV test within 6 weeks prior to registration

7. Age at least 18 years

8. ECOG performance status 0-2, ECOG 3 is only permitted if related to CLL or RT (e.g. due to anaemia or severe constitutional symptoms)

9. Life expectancy ≥ 3 months

10. Ability and willingness to provide written informed consent and to adhere to the study visit schedule and other protocol requirements

Exclusion criteria

1. Patients who did not respond to previous line of RT therapy (i.e. primary progressive patients)[2]

2. Patients with more than one prior line of RT therapy

1 For patients who start study treatment with elevated liver enzymes due to CLL/RT or Gilbert's syndrome, toxicity and AE reporting will follow CTCAE grading once these values further increase. E.g. if a patient starts with a bilirubin value of 2.0 mg/dl, which rises to 3.0 mg/dl after one cycle, this should be reported as grade 2 bilirubinemia (see CTCAE v5)

2 In cases with urgent need for treatment, a prephase treatment with steroids, vincristine (up to 2 mg IV) or cyclophosphamide (up to 200 mg^2 daily for max 3 days) can be administered at the discretion of the treating physician prior to enrolment or start of study medication.

3. Allogenic stem cell transplantation within the last 100 days or signs of active GVHD after prior allogeneic stem cell transplantation within any time

4. Patients with confirmed PML

5. Uncontrolled autoimmune condition

6. Malignancies other than CLL currently requiring systemic therapies (unless the malignant disease is in a stable remission at the discretion of the treating physician)

7. Active infection currently requiring systemic treatment

8. Any comorbidity or organ system impairment rated with a CIRS (cumulative illness rating scale) score of 4, excluding the eyes/ears/nose/throat/larynx organ system[1], or any other life-threatening illness, medical condition or organ system dysfunction that – in the investigator´s opinion could comprise the patients safety or interfere with the absorption or metabolism of the study drugs

9. Requirement of therapy with strong CYP3A4 inhibitors/ inducers

10. Requirement of therapy with phenprocoumon or other vitamin K antagonists.

11. Use of investigational agents, e.g. monoclonal antibodies or other experimental drugs within clinical trials, which might interfere with the study drug within 28 days (or 5 times half-life [$t_{1/2}$] of the compound, whichever is longer) prior to registration

12. Known hypersensitivity to tislelizumab, zanubrutinib or any of the excipients

13. Pregnant women and nursing mothers (a negative pregnancy test is required for all women of childbearing potential within 7 days before start of treatment)

14. Fertile men or women of childbearing potential unless:

1 This is to allow that patients who have sensory impairments, such as hardness of hearing plus impaired vision, can still be enrolled, despite 4 points on the CIRS scale. Infections of the upper respiratory tract should be recorded un-der the category "respiratory".

- surgically sterile or ≥ 2 years after the onset of menopause, or
- willing to use two methods of reliable contraception including one highly effective contraceptive method (Pearl Index <1) and one additional effective (barrier) method during study treatment and for 12 months after the end of study treatment.

15. Vaccination with a live vaccine <28 days prior to randomization
16. Legal incapacity
17. Prisoners or subjects who are institutionalized by regulatory or court order
18. Persons who are in dependence to the sponsor or an investigator

Names of investigational medicinal products (IMPs):

- Tislelizumab (BGB-A317)
- Zanubrutinib (BGB-3111)

Treatment plan:

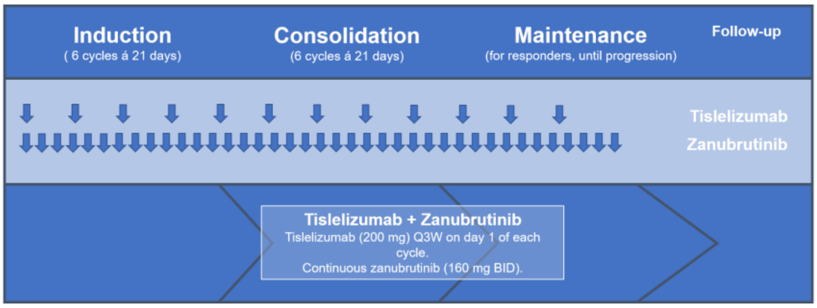

Dosage and method of administration of IMP:

Induction

Induction treatment consists of **6 cycles**, each with a duration of **21 days** (Q3W). Tislelizumab is administered intravenously on day 1 of each cycle. Continuous daily administration of zanubrutinib starts on day 1 of the first cycle as well.

Cycle 1-6:	Day 1:	Tislelizumab	200 mg iv
	QD:	Zanubrutinib	160 mg BID po

Consolidation

During consolidation, patients continue to receive both agents **over 6 cycles** (Q3W).

Cycle 7-12:	Day 1:	Tislelizumab	200 mg iv
	QD:	Zanubrutinib	160 mg BID po

Maintenance

Patients with response to therapy (CR, PR, and also SD) continue to take both agents until disease progression, non-tolerance or when receiving allogenic SCT for consolidation.

Duration of treatment:

Patients with response to therapy continue to take both agents until disease progression, non-tolerance or receiving allogenic SCT as consolidation.

Long-term follow up following the end of the study:

Patients will be followed up until 6 months after last study drug intake. To be able to collect long-term follow up data after the end of CLL-RT1 study, inclusion in the registry of the GCLLSG should be considered. For this purpose, each patient will be informed about the importance of long term follow data and asked for his/her consent to the long term follow-up within the GCLLSG registry. For patients with a written informed consent for the registry, data for overall survival, late toxicities such as secondary malignancies, further treatments and the course of the disease will be collected within the non-interventional GCLLSG registry after the end of the trial participation.

Interim safety analysis:

The first six patients will be part of an interim safety analysis, for which a close site monitoring will be maintained in order to take into account SAEs and AESIs. Special focus will be laid on:

- CTC° III/IV hematological toxicities related to study treatment, which require an intervention (e.g. additional monitoring, administration of G-CSF or blood transfusions),
- CTC° III/IV non-hematologic toxicities related to study treatment
- laboratory syndromes,
- cardiovascular and bleeding AEs, and
- AEs with a fatal outcome.

The interim safety analysis will be performed as soon as the first six patients have been treated for three cycles. The results from the interim safety analysis and all available data (also from other clinical trials) regarding the drugs used in this trial will be reviewed by the GPI, the coordinating physician, one statistician and the safety management team of the GCLLSG. This review will determine if the recruitment can be continued, if additional safety precautions and monitoring are needed or whether the trial will be prematurely stopped.

Stopping rules:

Any decision to prematurely terminate the study as a whole will be made

	by the sponsor in accordance with the regulatory and ethical principles. During the study, continuous monitoring of efficacy and toxicity will be performed.<br>Criteria for termination of the study as a whole are:<br>- An unexpectedly high rate of CTC°III/IV hematological and/or non-hematological AEs, cardiovascular and/or bleeding events in the patients from the interim safety analysis.<br>- An unacceptable profile or incidence rate of (serious) adverse events/ adverse events of special interest revealed in this or any other study in which at least one of the investigational products of this trial is administered.<br>- Demonstration that the study treatment is ineffective or only insufficiently active.<br>- Significant number of cases of death associated with the study treatment<br>- Any other factor that in the view of the sponsor constitutes an adequate reason for terminating the study as a whole.
Statistical methods and study assumptions:	For the analyses, the following patient populations will be defined:<br>- Full analysis set (FAS): The FAS comprises of all enrolled patients who received at least one dose of study medication in the third induction cycle (efficacy population). The FAS shall be used for analysis of all study endpoints except safety.<br>Patients with early discontinuation from study treatment (i.e. discontinuation prior to administration of third induction cycle) will be reported separately from the FAS.<br>- Safety population: The safety population is defined as all subjects enrolled in the study receiving at least one dose of trial treatment, whether withdrawn prematurely or not. The safety population shall be used for evaluating the safety endpoints.<br><br>The primary efficacy variable (primary endpoint) is the overall response rate (ORR) at interim staging after end of induction therapy (end of induction treatment response = EOIT). ORR is defined as the proportion of patients having achieved a CR or PR. Patients without any documented response assessment will be kept and labeled as 'non-responder' in the analysis.<br><br>Efficacy of the investigated regimen is assessed to be not effective if the ORR is less than 40 %. This boundary of efficacy of 40 % ORR corresponds to response rates observed in RS patients treated with conventional chemoimmunotherapy. It is assumed to improve the ORR to at least 60 % with the investigated regimen.

Sample size calculation: The primary endpoint ORR at end of induction therapy was used to determine the sample size of the study. The following study assumptions are considered:

- As stated before the ORR for a conventional regimen is assumed to be 40 % (=P0) with corresponding null hypothesis H0: ORR ≤ 0.40 and alternative hypothesis H1: ORR > 0.40.
- The investigated regimen is considered potentially useful and worthy of further research if we can reject the null hypothesis in favor of the alternative hypothesis.
- The type I error is set to α = 2.5 % and defines the chance that the investigated regimen will be investigated further although the true ORR is lower or equal to 40 %.
- The type II error is the chance that an effective treatment will not be studied further. It is assumed to improve the ORR to at least 60 % (=P1) with the investigated regimen. The type II error should not exceed β = 20 %, so that it is aimed to achieve a power of at least (1 - β) = 80 % at the assumed ORR P1.

According to the above determined study parameters a one-sided one-sample binomial-test with an overall significance level of 2.5 % provides the sample size N=48, such that statistical significance is achieved with a power of 80 %.

The following table describes the minimum number of responders (i.e. having a CR or PR) that are required to warrant further investigation of the new regimen based on different numbers of analyzable patients:

Number of analyzable patients	Minimum number of responders
51, 50	27
49, 48	26
47, 46	25
45, 44, 43	24
42, 41	23
40, 39	22
38, 37	21
36	20

Sample size calculations were performed with EAST 5 software and validated with Binomial tables.

Recruitment strategy:	10 sites in Germany, 1 site in Austria plus 1 site in Denmark	
Study duration:	Start of recruitment	Q1/2020
	End of recruitment	Q1/2023
	Expected End of study	Q1/2024
Statistician:	Dr. Dipl.-Math. Sandra Robrecht<br>Department of Internal Medicine I, Study office GCLLSG, University of Cologne, Kerpener Str. 62, 50924 Köln, Germany	
GCP conformance:	The present trial will be conducted in accordance with the valid versions of the trial protocol and the internationally recognized Good Clinical Practice Guidelines (ICH-GCP), including archiving of essential documents.	

IV. Study assessment table

		Informed consent, Incl. / Excl. Criteria	HIV / HBV / HCV test[2]	ECG	LVEF[3]	Urinalysis [16]	Pregnancy test[4]	G8[17]	CIRS Score / Medical history[5]	Concomitant medication (incl. premedication)[5]	ECOG / disease-related symptoms[6]	Height / Weight / BSA	Physical examination	Radiological assessment[7]	Complete blood count[8]	Serum chemistry[9]	Serum parameters (central lab Cologne)*	Immunophenotyping (central lab Cologne)*	FISH cytogenetics, TP53 and IGHV (central lab Ulm)*	Lymph node biopsy	Histopathological report (GCLLSG Central Office)	Biopsy sample [10] (Cologne)	Accompanying scientific program (Cologne)*	Bone marrow aspirate/biopsy[11]	Response assessment	New treatment and survival status	Tislelizumab (BGB-A317) i.v. administration	Zanubrutinib (BGB-3111) p.o. administration	Radiation[12]	Drug accountability	(serious) adverse events
**Screening/Baseline[1]**		X	X	X	O	X	(X)	(X)	X	CONTINUOUS REPORTING	X	X	X	X	X	X	X	X	X	X	X	X	X	(X)							
**Cycle 1-6**	**day 1**						(X)						X		X	X										CONTINUOUS REPORTING	X			X	CONTINUOUS REPORTING
	**days 1-21**														O	O												X			
**Interim staging**				X[15]		X	(X)	(X)			X		X	X	X	X							X	(X)	X				O		
**Cycle 7-(12)** (responders until PD)	**day 1**						(X)				X		X		X	X											X			X	
	**days 1-21**														O	O												X			
**Final restaging (after consolidation)**				X		X	(X)	(X)			X		X	X	X	X							X	(X)	X					X	
**Maintenance staging [13]**							(X)						X	(X)	X	X									X						
**Follow-up visits [14]**													X	(X)	X	X									X						
**Progressive disease**											X		X	X	X	X			X	X	X	X	X	(X)	X						

*) for details regarding the accompanying scientific program please see table IV
X = assessment mandatory, (x) = assessment mandatory in certain patients/certain situations, O = assessment recommended and to the investigator´s discretion, but not documented in the CRF.

[1]) Screening: once all screening CRF pages and results of the central diagnostics (immunophenotyping, serum parameters and cytogenetics) are available, these will be checked by a GCLLSG study physicain for approval of the patient; treatment should be initiated within 28 days after approval of the patient. Baseline: after approval of the patient and before start of therapy (e.g. blood sampling for accompanying scientific program).
[2]) Exclusion of HIV, HBV and HCV infection has to be performed ≤6 weeks prior to registration. Patients who are HBsAg negative/anti-HBc positive may be included if PCR for HBV DNA (with a lower limit of detection of the order of 10 WHO IU/mL) is negative and HBV-DNA PCR is performed every month until 1 year after last dosage of tislelizumab. If the HBV DNA assay becomes positive, patients should pre-emptively be treated with tenofovir, entecavir or la- mivudine) for at least 12 months after the last cycle of therapy or be referred to a gastroenterologist for management
[3]) Cardiologic diagnostics should be performed at the investigator´s discretion; in case of pathological findings, please report in the CIRS score page.
[4]) Pregnancy test is required for all women of childbearing potential (for definition see chapter *8.10.1.6. Teratogenicity and mutagenicity*) at baseline, every month, delayed menstruations (>1 month) and one month after last drug intake.
[5]) Medical history includes disease stage (Current Binet and Rai stage and Binet stage at initial diagnosis), prior therapies (treatment history) and concomitant medication 28 days prior to screening
[6]) Disease related symptoms include B-symtpoms like fever, weight loss, night sweat
[7])Radiological assessment of lymphadenopathy, liver and spleen using CT, PET-CT or MRI technique (head/neck, chest, abdomen and pelvis/inguinal region) is mandatory at screening, interim staging and final restaging. Further imaging can be performed at the investigator's discretion, e.g. in case of a suspected PD. It is recommended to perform ultrasound examinations (at least of the abdomen) at the stagings inbetween. PET-CT is mandatory at final restaging only, in which case an additional CT scan is not necessary. Written radiology reports should be provided to the study office.
[8]) including differential count: white blood cell count (WBC), hemoglobin, platelet count, absolute neutrophil and absolute lymphocyte count (ANC, ALC)
[9]) serum creatinine, total bilirubin, AST, ALT, LDH [are required at all timepoints during treatment]; sodium, potassium, calcium, phosphate, chloride and uric acid [during induction treatment]; immunoglobulins (IgA, IgM, IgG, IgE) and direct antiglobulin Coombs test [at screening and final restaging]
[10]) Submission of formalin-fixed paraffin embedded tumor tissue sample blocks is mandatory. If blocks are not available for shipment, 20 slides should be freshly cut and submitted to the testing laboratory within 14 days from site slide sectioning date otherwise a new specimen will be requested.
[11]) Bone marrow aspirate/biopsy may be performed whenever clinically indicated, e.g. it is recommended in case of uncertain cytopenia
[12]) Irradiation of nodal bulks or extranodular lesions is allowed after induction therapy at the discretion of the physician. A separate documentation for radiation and prior discussion with the PI is mandatory.
[13]) Maintenance restaging should be done every three cycles based on physical examination, blood count and serum chemistry. When PD is suspected, a CT scan is mandatory to assess new lesions.
[14]) Follow-up visits should be performed monthly for 6 months after last study drug intake to capture survival and subsequent treatments
[15]) ECG should be performed every six months, in particular to detect arrythmia like atrial fibrillation.
[16]) Urine testing strip every 6 months to check for urine glucose, occult blood and urobilinogen
[17]) G8 questionnaire has to be performed for basic geriatric assessment in patients >65 years in Austria at time point screening and is voluntary at other indicated time points. For other participating countries the completion of the G8 questionnaire is voluntary. The G8 questionnaire will be filled in paper-based only, but not into the eCRF (see Appendix B). A copy of the completed Appendix B will be provided to the GCLLSG office.

V. Accompanying scientific program

	TIMEPOINTS				MATERIAL	SHIPMENT DAYS	LABORATORY
	Screening[1]	Interim Staging [after induction]	Final restaging [after consolidation]	Progressive disease			
**Immunophenotyping**	X				10ml EDTA	Mo-Do	Kreuzer, Cologne
**Serum parameters** (Beta-2MG &TK)	X				5ml Serum	Mo-Do	Malchau, Cologne
**FISH cytogenetics & molecular genetics**[2]	X			X	30ml Heparin	Mo-Do	Stilgenbauer, Ulm
**ctDNA**	X	X	X	X	10 ml ctDNA tube	Mo-Do	Biobank, Cologne
**Karyotyping**	X				10ml Heparin	Mo-Do	Kreuzer, Cologne
**Sequencing, epigenetics, immunol. assays**[3]	X	X	X	X	40ml EDTA	Mo-Do	Biobank, Cologne
**Formalin-fixed paraffin embedded tumor tissue** [4]	X			X	FFP biopsy	Mo-Do	Biobank, Cologne

Blood sampling should always be performed before administration of study drug!

[1]) Screening: after obtaining informed consent and before administration of treatment.
[2]) Including BTK/PLCg2-Mutation in patients with prior BTK-Inhibitor treatment
[3]) Please also send bone marrow (5-10ml EDTA) together with a peripheral blood sample each time a bone marrow aspirate is performed.
[4]) Submission of formalin-fixed paraffin embedded tumor tissue sample blocks is mandatory. If blocks are not available for shipment, 20 slides should be freshly cut and submitted to the testing laboratory within 14 days from site slide sectioning date otherwise a new specimen will be requested.

I. Table of contents

I. Signatures 2

II. Summary of Changes 3

III. Synopsis 5

IV. Study assessment table 16

I. Table of contents 18

VI. List of tables 23

VII. Abbreviations 24

1. Introduction 26

1.1. Chronic lymphocytic leukemia 26

1.2. Malignant disease transformation 26

2. General aspects of the drugs used in the trial 28

2.1. Zanubrutinib (BGB-3111) 28

2.2. Tislelizumab (BGB-A317) 28

3. Objectives of the clinical trial 30

3.1. Rationale for the clinical trial 30

3.2. Benefit/Risk Assessment 30

3.2.1 Benefit-Risk Assessment regarding Covid-19 pandemic 31

3.3. Primary objective 33

3.4. Secondary and exploratory objectives 33

4. Organizational and administrative aspects of the trial 35

4.1. Sponsor 35

4.2. Chairman of the GCLLSG 35

4.3. Global Principal Investigator and Sponsor´s representative 35

4.4. Coordinating Physician 35

4.5. Head of the GCLLSG study office 35

4.6. Statistics 35

4.7. Project Management 35

4.8. Safety management 35

4.9. Other trial management 35

4.10. Monitoring 36

4.11. Investigators and trial sites 36

4.11.1. Requirements for investigators and trial sites 36

4.12. Financing 36

4.13. Data Monitoring Committee 36

5. Central laboratory assessment 37

5.1. Immunophenotyping 37

5.2. Serum parameters 37

5.3. Routine Genetic analyses 37

5.4. Tumor biopsies 37
5.5. Accompanying scientific program 38
5.6. Optional scientific analyses 38

6. Trial population 39

7. Trial conduct 41
7.1. Trial design 41
7.1.1. Induction 41
7.1.2. Consolidation 42
7.1.3. Maintenance 42
7.1.4. Treatment discontinuation 42
7.1.5. Follow up 42
7.2. Interim safety analysis 42
7.3. Trial duration 43
7.3.1. Planned study timelines 43
7.4. Registration and screening 43
7.5. Recruitment strategy and stratification 44
7.5.1. Gender distribution 44
7.5.2. Withdrawal 44
7.5.3. End of study 44
7.5.4. End of trial 44
7.6. GCLLSG Registry: Long-term follow up outside the CLL-RT1-trial 45

8. Study Medication 46
8.1. Ordering study medication 46
8.2. Tislelizumab (BGB-A317) 46
8.2.1. Concomitant medications 46
8.2.2. Preparation of tislelizumab infusions 46
8.2.3. Administration of tislelizumab 47
8.2.3.1. Management of infusion-related reactions (IRRs) 47
8.3. Zanubrutinib (BGB-3111) 49
8.3.1. Administration of zanubrutinib 49
8.4. Concomitant therapy 50
8.4.1. Permitted medications 50
8.4.1.1. Hematopoietic growth factors 50
8.4.1.2. Infectious prophylaxis 50
8.4.1.3. Medication and food to be used with caution during zanubrutinib treatment 51
8.4.1.4. CYP3A4 inhibitors/-inducers 51
8.4.1.5. Anticoagulants 53
8.4.2. Surgery 53
8.5. Prohibited medication during entire study 53
8.6. Dose and schedule modifications 54
8.6.1. Dose and schedule modifications for tislelizumab 54
8.6.1.1. Management of immune-related AEs caused by tislelizumab 54
8.6.2. Dose and schedule modifications zanubrutinib 66

8.6.2.1. Dose interruption and modification 67
8.6.2.2. Zanubrutinib dose reduction for hematologic toxicity 67
8.6.2.3. Zanubrutinib dose reduction for non-haematologic toxicity 67
8.7. Reasons for permanent discontinuation of all study treatment 68
8.8. Potential risks 68
8.8.1. Potential risks relevant with all study drugs 68
8.8.1.1. Cytopenias and infections 68
8.8.1.2. Immune related disorders 69
8.8.1.3. Teratogenicity and mutagenicity 70
1.1.1. Cardiac adverse events 72
8.8.2. Further potential risks with tislelizumab 73
8.8.3. Further potential risks with zanubrutinib 73
8.9. Special Situation Report Form 74

9. Measurement of efficacy and safety variables 75
9.1. Efficacy variables 75
9.2. Safety variables 75
9.3. Laboratory analyses 75
9.4. Response assessment 76
9.4.1. Definition of measurable sites of disease 76
9.4.2. Response criteria 76

10. Safety 81
10.1. Reporting periods 81
10.2. Definitions of AE, ADR, SAE, SADR, SUSAR 82
10.2.1. Adverse event (AE) and adverse drug reaction (ADR) 82
10.2.2. Adverse events of special interest (AESIs) 82
10.2.3. Adverse events of particular interest (AEPIs) 82
10.2.4. Serious adverse events (SAEs) and serious adverse reactions (SADRs) 82
10.2.5. Suspected unexpected serious adverse reactions (SUSAR) 83
10.3. Exceptions and specialties 83
10.3.1. Lab abnormalities 83
10.3.2. Concomitant diseases 83
10.3.3. Pregnancy 83
10.3.4. AEs of special interest 84
10.3.5. AEs of particular interest 84
10.3.6. Documentation and follow up of AE and SAE 85
10.3.7. Severity of adverse events 85
10.3.8. Causal relationship between AE and study drug 86
10.4. Reporting of SAE, pregnancy and changes in risk-benefit assessment 86
10.4.1. Reports from the investigator to the sponsor 86
10.5. Assessment and reporting responsibilities of the sponsor 87
10.5.1. Assessment of event by sponsor 87
10.5.2. Reference safety information documents 87
10.5.3. Notification of ethics committee and competent supreme federal authority 87

10.5.4. Review and reporting of changes in the risk-benefit ratio 87
10.5.5. Informing the investigators 88
10.5.6. Informing the marketing authorization holder 88
10.5.7. Development Safety Update report of trial patients 88
10.6. SAE reporting after the end of the clinical trial 88

11. Data quality assurance 89
11.1. Monitoring 89
11.1.1. Audits/Inspections 89
11.2. Documentation 89
11.3. Data management 90
11.4. Archiving 90

12. Closure of trial sites/Premature termination of the clinical trial 91
12.1. Closure of trial sites 91
12.2. Premature termination of trial 91
12.2.1. Stopping rules for the trial 91

13. Ethical and regulatory aspects 92
13.1. Ethical considerations 92
13.2. Good Clinical Practice and regulatory requirements 92
13.3. Obtaining informed consent 92
13.4. Insurance of patients in the trial 93
13.5. Data protection 93

14. Statistical methods and sample size calculation 95
14.1. General consideration 95
14.2. Sample size 95
14.3. Trial populations 96
14.4. General study overview 96
14.4.1. Conduct of study 96
14.4.2. Observation time 96
14.4.3. Demographic and baseline characteristics 96
14.4.4. Primary endpoint 96
14.4.5. Analysis of primary endpoint 96
14.5. Secondary efficacy endpoints 97
14.5.1. Response rates 97
14.5.1.1. Overall response rate (ORR) after induction therapy according to IWCLL criteria 97
14.5.1.2. Overall response rate (ORR) after consolidation therapy 97
14.5.2. Time-to-event endpoints 97
14.5.2.1. Progression-free survival (PFS) 97
14.5.2.2. Overall survival (OS) 98
14.5.2.3. Duration of response 98
14.5.2.4. Time to next treatment (TTNT) 98
14.6. Safety analyses 98
14.6.1. Treatment exposure 98
14.6.2. Adverse events 98

14.6.3. Mortality 99

14.6.4. Exploratory analyses 99

14.7. Analysis time-points 99

15. Final report and publication 100

15.1. Final report 100

15.2. Publication 100

16. Amendments to the trial protocol 101

17. Appendices 102

18. References 103

VI. List of tables

Table 1 Response criteria according to iwCLL guidelines 76
Table 2 Response criteria according to Lugano Classification 77
Table 3 Overview of reporting periods and documentation 81

VII. Abbreviations

Abbreviation	Meaning
ADME	Absorption, distribution, metabolism, and excretion
ADR	Adverse drug reaction
AE	Adverse Event
AEPI	Adverse event of particular interest
AESI	Adverse events of special interest
AIHA	Autoimmune hemolytic anemia
ALC	Absolute lymphocyte count
ALT	Alanine Aminotransferase
ANC	Absolute neutrophil count
ASCO	American Society of Clinical Oncology
AST	Aspartate aminotransferase
BfArM	Federal Institute for Drugs and Medical Devices (Bundesinstitut für Arzneimittel und Medizinprodukte)
BR	Bendamustine, Rituximab
BSA	Body surface area
B2MG	Beta-2-microglobulin
CBC	Complete blood count
CD	Cluster of differentiation
CIRS	Cumulative Illness Rating Scale
CLL	Chronic lymphocytic leukemia
CNS	Central nervous system
CR	Complete response
CRF	Case Report Form
CRi	Complete response with incomplete bone marrow recovery
CT	Computerized tomography (scan)
CTC	Common Toxicity Criteria
DNA	Deoxyribonucleic acid
EBMT	European Society for Blood and Marrow Transplantation
EC	Ethics committee
ECG	Electrocardiogram
ECOG	Eastern Cooperative Oncology Group
EFS	Event free survival
EOIT	End of induction treatment response
EOS	End of study
ERIC	European Research Initiative on CLL
EU	European Union
FCR	Fludarabine, Cyclophosphamide, Rituximab
FDA	U.S: Food and Drug Administration
FISH	Fluorescence in situ hybridization
FL	First-line
GCLLSG	German CLL Study Group
GCP	Good Clinical Practice
GI	gastrointestinal
G-CSF	Granulocyte colony stimulating factor
GPI	Global Principal Investigator
Hb	Hemoglobin
HBs	Hepatitis B surface
HBsAg	Hepatitis B surface antigen
HBV	Hepatitis B virus
HCV	Hepatitis C virus
HIV	Human Immunodeficiency virus
IB	Investigator´s brochure

ICH	International Conference on Harmonization of Technical Requirements for Registration of Pharmaceuticals for Human Use
i.e.	Id est
IMP	Investigational medicinal product
IRR	Infusion related reaction
irAE	immune related adverse event
i.v.	intravenous
iwCLL	International Workshop on Chronic Lymphocytic Leukemia
ITP	Idiopathic thrombocytopenic purpura
LKP	Leiter der klinischen Prüfung, Global Principal Investigator
LMWH	Low molecular weight heparin
LVEF	Left ventricular ejection fraction
MedDRA	Medical Dictionary for Regulatory Activities
NaCl	Sodium Chloride
NCI	National Cancer Institute
NHL	Non-Hodgkin's lymphoma
nPR	Nodular partial response
ORR	Overall response rate
OS	Overall survival
PD	Progressive disease
PEI	Paul-Ehrlich-Institut
PFS	Progression free survival
PI	Principle investigator
PML	Progressive multifocal leukoencephalopathy
PQC	Product quality complaint
PR	Partial response
PRCA	Pure red cell apasia
RNA	Ribonucleic acid
r/r	relapsed/refractory
SAE	Serious Adverse Event
SCT	stem cell transplantation
SD	Stable disease
SMPC	Summary of product characteristics
SUSAR	Suspected Unexpected Serious Adverse Reaction
TEAE	Treatment-Emergent Adverse Event
TK	Thymidine kinase
TTNT	time to next treatment
ULN	Upper limit of normal
WBC	White blood count
WHO	World health organization

1. Introduction

1.1. Chronic lymphocytic leukemia

Chronic lymphocytic leukemia (CLL) is the most common leukemia in adults in the western countries with an age-adjusted incidence rate of 4.3 per 100,000 inhabitants per year in the United States [14, 15]. The incidence rate of CLL rises with age [14], hence the prevalence of CLL is likely to increase further due to demographic changes.

According to the WHO Classification of Tumors of Hematopoietic and Lymphoid Tissues, CLL belongs to the mature B-cell neoplasms [16]. CLL is characterized by clonal proliferation and accumulation of neoplastic B-cells within the blood, bone marrow and secondary lymphatic organs [17]. CLL cells typically co-express the T-cell antigen CD5, and B-cell antigens CD19, CD20, and CD23; while compared to normal B cells the levels of CD20, and CD79b on CLL cells are characteristically low [18-20]. Each clone of CLL cells is restricted to either kappa or lambda immunoglobulin light chain expression [18, 20].

CLL cells are dependent on external signals from the microenvironment, such as antigens, cytokines, and cell-cell interactions, which seems to modulate the CLL cells' resistance to apoptosis[21]. One of the signaling pathways stimulated by the microenvironment is the B-cell receptor (BCR) pathway, that regulates apoptosis through activation of NF-KB through different tyrosine kinases[22], such as phosphatidyl-Insositol-3-kinase (PI3K) and Bruton´s-tyrosine-kinase (BTK). Bcl-2 (B-cell lymphoma 2), a regulator protein for apoptosis, which is classified as an oncogene because it inhibits cell death (anti-apoptotic), plays a role in the pathogenesis and treatment resistance of several malignancies, including CLL [23, 24].

The clinical course of CLL is highly variable, some patients live for decades without treatment, whereas others experience an aggressive course, that resembles an acute leukemia [25]. Yet, several parameters of prognostic impact have been identified [26]. The extent of disease, which is mainly reflected by enlargement of lymph nodes, liver and spleen and impairment of hematopoiesis, is currently used to define different stages of disease (Rai and Binet clinical staging systems), that are related to prognosis [27, 28]. Furthermore, chromosomal abnormalities like deletion 17p, 11q, trisomy 12 or deletion 13q [29], as well as mutations of the immunoglobulin heavy variable chain (IGHV) gene [30, 31] or the TP53 gene [32] influence the course of the disease. Also, patient related factors such as age and burden of comorbidity play a role for the patient´s prognosis and also for treatment decisions. The international prognostic index CLL-IPI clearly separates four risk groups based on the following five factors: age, clinical stage, elevated beta-2-microglobuline, as well as the genetic parameters IGHV mutational status and deletion 17p/TP53 mutation [33].

1.2. Malignant disease transformation

Richter syndrome (RS) or Richter transformation (RT) describes the rapid development of a histologically confirmed aggressive lymphoma, in most cases a diffuse large B cell lymphoma (DLBCL), in patients with CLL. The incidence rates of RT among CLL patients range from 2 to 10% [1]. RT can occur at any time during the course of CLL. Risk factors for development of RT include intrinsic biological features like TP53 mutations or 17p deletions as well as therapy-related factors as exposure to purine analogues like fludarabine [2]. However, up to one third of patients with RT are treatment naïve CLL patients [3].

RT patients have a very poor prognosis with a median OS of 6-8 moths. There is no established standard of care for RT and most patients are treated comparably to de-novo DLBCL patients with chemoimmunotherapies like R-CHOP or R-DHAP. While this regimen achieves high response rates in de-novo

DLBCL and even cures up to 80% of patients, patients with RT are rarely cured by chemoimmunotherapy and response rates are considerably lower between 20-60% [3]. Given the poor prognosis, fit patients are considered for allogenic transplantation once they respond to therapy. However, as CLL is a disease of the elderly with a median age of 72 years, most patients with RT are not fit enough to undergo allogenic transplantation.

The advent of a variety of novel antibodies and targeted drugs allows for new therapeutic approaches to address the unmet clinically need for a better care for RT patients.

Zanubrutinib (BGB-3111) is an orally bioavailable selective, irreversible inhibitor of Bruton's tyrosine kinase (BTK) that is currently developed in a variety of B-cell malignancies, including CLL and DLBCL. BTK is a well-established target for CLL treatment, as its inhibition by currently licensed agents like ibrutinib disrupts the BCR-dependent survival and proliferation of CLL cells. Pleiotropic effects of ibrutinib lead to distinct toxicities, particularly bleeding events and arrhythmia. Zanubrutinib is suggested to be more selective than ibrutinib and have less off target effects on other kinases like EGFR, JAK3 or ITK. Preclinical as well as early clinical data indicate that zanubrutinib has less side effects and a more favorable pharmacokinetic and pharmacodynamic profile [4].

Tislelizumab (BGB-A317) is a humanized IgG4 variant monoclonal antibody with no Fc gamma receptor binding that targets the programmed cell death-1 (PD-1) receptor. Expression of PD-1 is a mechanism by which malignant cells evade the immune system response. By blocking the interaction between PD-1 and its ligands, T-cells are allowed to recognize and kill tumor cells. So far, tislelizumab has shown clinical activity in a variety of tumors and is currently being tested in solid as well as hematological malignancies. A recent phase Ib trial has shown a manageable toxicity profile of the combination of zanubrutinib and tislelizumab in different B-cell malignancies [5].

Given that high PD-1 expression has been observed in patients with lymphoid malignancies, checkpoint inhibitors are promising candidates for treatment of RT. Previous data have shown that effective eradication of DLBCL cells in the bone marrow of RT patients can be achieved with single-agent PD-1 inhibitors [6]. However, persistence of CLL infiltration was observed as well, which suggests that a combinational approach might be indicated for effective treatment.

Currently, two trials are testing combinational approaches with nivolumab, a PD-1 inhibitor, plus ibrutinib and early interim analyses showed good response rates in pre-treated patients with RT [7, 8]. Taken together, preclinical as well as early clinical data provide a good rationale to investigate on a combination of PD-1 inhibition plus BTK inhibition in previously untreated patients with RT.

This prospective phase-II-trial will investigate a combinational regime of the PD-1 inhibitor tislelizumab and the BTK inhibitor zanubrutinib. The treatment schedule consists of 6 cycles of induction therapy (21-day cycles) during which tislelizumab will be administered once at a fixed dose, followed by 6 additional cycles of tislelizumab consolidation therapy. Zanubrutinib will be given two times daily (BID) from day 1 of cycle 1. Patients who show response to therapy after 12 cycles of therapy will continue until disease progression or unacceptable toxicities.

2. General aspects of the drugs used in the trial

2.1. Zanubrutinib (BGB-3111)

BCR signaling is essential for normal B-cell development, differentiation, function, and survival [34, 35]. The aberrant BCR pathway is implicated in the pathogenesis of several B-cell malignancies including mantle cell lymphoma (MCL), chronic lymphocytic leukemia (CLL), follicular lymphoma (FL), Waldenström's macroglobulinemia (WM), and diffuse large B-cell lymphoma (DLBCL) [36]. A member of the TEC family of kinases, BTK is a signaling molecule positioned within the BCR signaling cascade; it was initially identified as the defective protein in human X-linked agammaglobulinemia (XLA; also known as Bruton's agammaglobulinemia), which is characterized by a complete lack of mature peripheral B cells and low levels of serum Ig [37]. BTK is predominantly expressed in B lymphocytes at various stages of development. Activation of BTK in B cells initiates a series of signaling events, including recruitment of BTK to the plasma membrane, autophosphorylation at Tyr223, activation of phospholipase Cγ2, subsequent NFγB activation, and expression of genes involved in proliferation and survival [38, 39].

Inhibition of BTK has emerged as a promising strategy for targeting B-cell malignancies. Ibrutinib, the first-in-class BTK inhibitor, is approved for treatment of various BCR-driven B-cell malignancies. Similarly, other BTK inhibitors, such as acalabrutinib and zanubrutinib, are in development for treatment of B-cell malignancies.

Zanubrutinib (BGB-3111) is an irreversible BTK kinase inhibitor that is suggested to be more specific than ibrutinib. Biochemical assays showed that zanubrutinib was more selective than ibrutinib for the inhibition of kinase activity of BTK vs. EGFR, FGR, ITK, FRK, JAK3 and others. It has also shown more anti-tumor activity than ibrutinib in human MCL and DLBCL xenograft models.

Several phase I - III trials have been or are currently conducted with zanubrutinib as single agent or in combination with other compounds, including tislelizumab. Apart from pharmacokinetic phase I trials in healthy adult volunteers (BGB-3111-103 and -105), in which no deaths or SAEs were reported and which established a target dose level of 160 mg BID, several trials also addressed treatment of various NHL. 256 patients with CLL, NHL, WM, HCL or RT were treated within BGB-3111-AU003. The most frequent treatment emergent adverse events (TEAE) were upper respiratory tract infections, contusions and diarrhea. 9 fatal AEs were reported (1 in WM, 8 in NHL, none in CLL or RT), mainly related to infections. Regarding efficacy, ORR was 58%, the majority of which were partial responses (45%) as expected with single agent BTK inhibitors. Another ongoing phase II trial with single agent zanubrutinib in 30 patients with CLL, NHL or WM showed comparable results (BGB-3111-1002), most frequent TEAEs were neutropenia, upper respiratory tract infections and anaemia. Combinational strategies, e.g. combinations with CD20-antibodies like obinutuzumab (BGB-3111-GA101-001) or PD-1 inhibitors like tislelizumab (BGB-3111-A317-001) are currently being conducted as well (see section 2.2). Further data have been published in the drug's IB and were presented as congress abstracts [40].

Zanubrutinib is primarily subject to hepatic metabolization by CYP3A and is a moderate inhibitor of CYP2C8, CYP2C9, and CYP2C19. Preclinical testing suggests an elimination half-life between 1 to 4 hours after single-dose oral administration.

Detailed information on pharmacological characteristics are provided in the current investigator's brochure.

2.2. Tislelizumab (BGB-A317)

PD-1 is mainly expressed in activated T-cells, including cluster of differentiation (CD)8+ cytotoxic T-lymphocytes and CD4+ T-helper lymphocytes [41, 42]. It is believed that PD-1 plays an important role

in immune modulation of tumor progression by regulating the key inhibitory signaling in the T-cells when engaged by its ligands. The PD-1 signaling cascade negatively regulates T-cell receptor (TCR) and attenuates T-cell proliferation and functional activities, leading to T-cell exhaustion. PD-1 expression is markedly up-regulated in tumor-infiltrating lymphocytes, while the expression of PD-1 ligand PD-L1 is significantly increased in tumor cells and tumor-associated immune cells in the presence of stimulating cytokines such as interferon-gamma (IFN-γ) and interferon-alpha (IFN-α) in the tumor microenvironment. Furthermore, the increased PD-1 expression in tumor-infiltrating lymphocytes and/or program death ligand-1 (PD-L1) expression in tumor and tumor-associated stromal cells is observed in many types of human solid and lymphoid malignancies [43]. Currently approved PD-1 inhibitors include nivolumab and pembrolizumab, both licensed by the FDA and EMA for treatment of, among other indications, NSCLC, Hodgkin's lymphoma and malignant melanoma.

Tislelizumab (BGB-A317) is a humanized immunoglobulin G4 (IgG4) variant monoclonal antibody against the immune check point-inhibitory receptor, programmed cell death-1 (PD-1). It binds to the extracellular domain of human PD-1 with high specificity and affinity. Tislelizumab competitively blocks the binding of both PD-L1 and program death ligand-2 (PD-L2), inhibiting PD-1 mediated negative signaling in T-cells. In in vitro cell-based assays, the humanized antibody consistently and dose-dependently enhanced the functional activity of human T-cells and pre-activated, primary peripheral blood mononuclear cells (PBMCs). Thereby, it demonstrated anti-tumor activity in several human cancer xenograft models.

Several phase I – II clinical trials have been or are currently been conducted in patients with malignant, mostly advanced tumors. Based on pharmacokinetic, safety and efficacy form the first in-human trial with tislelizumab (BGB-A317-001), a flat dose of 200 mg IV Q3W was established for investigation in further clinical trials. A variety of solid tumors, including gastrointestinal, gynecological and pulmonary cancers, has been included in these trials so far. Most common TEAEs were fatigue, nausea, decreased appetite and diarrhea. Roughly 20% of patients experienced at least one immune-related TEAE, most of which were dermatological, endocrine or hepatobiliary. Two fatal immune-related TEAEs (pneumonitis and acute hepatitis) have been reported, further details and case reports are provided in the drug's IB.

Combinational strategies with tislelizumab plus zanubrutinib are investigated in ongoing phase 1b trial for patients with B-cell NHL (BGB-3111-A317-001). So far, the study did not show any relevant toxic interactions between both compounds in 25 patients; most frequent TEAEs were upper respiratory tract infections, diarrhea and cough. Single cases of grade 4 thrombocytopenia or anemia were observed as well as two cases of grade 3 hemolytic transfusion reactions and pneumonitis, otherwise no additional toxicities or safety signals have been observed so far with the combined PD-1 and BTK inhibition.

Overall, the safety profile of tislelizumab seems to be consistent with the therapeutic class of the drug, i.e. PD-1 inhibitors, with a relatively low rate of drug-related Grade 3 or above toxicity.

3. Objectives of the clinical trial

3.1. Rationale for the clinical trial

RT remains one of the biggest challenges in the treatment and management of CLL. While considerable progress has been made in the treatment of CLL, the prognosis of CLL patients with malignant disease transformation still is very poor and reported median OS is between 6 to 8 months. Conventional approaches with chemo- and chemoimmunotherapy have largely failed to improve response rates in RT patients. However, as the established treatment approach for de-novo DLBCL is chemoimmunotherapy with R-CHOP, this has become the most commonly used regimen for lack of alternative strategies, despite poor efficacy. Patients being fit enough for allogeneic transplantation are undergoing this procedure after induction with R-CHOP. However, the majority of patients are not suitable for transplantation and relapse quickly. Hence, there is urgent need to improve therapy of RT by testing new compounds and combinations for treatment of this disease. Based on the available pre-clinical and preliminary clinical data on checkpoint inhibition plus BTK inhibition [5, 41, 44, 45], the current trial will systematically assess the safety and toxicity of tislelizumab, a PD-1 inhibitor, plus zanubrutinib, a BTK inhibitor in patients with RT.

3.2. Benefit/Risk Assessment

Zanubrutinib and tislelizumab have different mechanisms of action, however, preclinical studies have suggested a synergistic effect of PD1-inhibition and BTK-inhibition [45]. Given the poor response to chemotherapeutic approaches, recent results from phase I and phase II trials with combinations of checkpoint inhibitors and BTK inhibitors in a variety of NHL showed promising response rates in heavily pretreated patients (see literature review in 1.2).

Zanubrutinib

Zanubrutinib (BGB-3111) is an orally bioavailable selective, irreversible inhibitor of Bruton's tyrosine kinase (BTK) that is currently developed in a variety of B-cell malignancies, including CLL and DLBCL. BTK is a well-established target for CLL treatment, as its inhibition by currently licensed agents like ibrutinib disrupts the BCR-dependent survival and proliferation of CLL cells. Pleiotropic effects of ibrutinib lead to distinct toxicities, particularly bleeding events and arrhythmia. Zanubrutinib is suggested to be more selective than ibrutinib and have less off target effects on other kinases like EGFR, JAK3 or ITK. Preclinical as well as early clinical data indicate that zanubrutinib has less side effects and a more favorable pharmacokinetic and pharmacodynamic profile [4]. The compound has been tested in over 1200 patients within various phase I to III trials, including a current head-to-head comparison with ibrutinib, the first-in-class BTK inhibitor.

Tislelizumab

Tislelizumab (BGB-A317) is a humanized IgG4 variant monoclonal antibody with no Fc gamma receptor binding that targets the programmed cell death-1 (PD-1) receptor. Expression of PD-1 is a mechanism by which malignant cells evade the immune system response. By blocking the interaction between PD-1 and its ligands, T-cells are allowed to recognize and kill tumor cells. So far, tislelizumab has shown clinical activity in a variety of tumors and is currently being tested in solid as well as hematological malignancies. A recent phase Ib trial has shown a manageable toxicity profile of the combination of zanubrutinib and tislelizumab in different B-cell malignancies [5]. Overall, more than 400 patients have been treated with tislelizumab at dose levels similar to this trial.

Possible overlapping toxicities and risk mitigation strategy

The safety profiles of both drugs seem to be compatible with little overlapping toxicities. The main expected overlap in toxicities concerns hematological toxicities, in particular anemia and leukopenia, which have been commonly observed in patients treated with single agent tislelizumab and single agent zanubrutinib. *The protocol provides detailed guidance including permitted medications and dose reduction and discontinuation recommendations to reduce the risks to patients' safety. (chapter 8.4.1.1 and 8.4.1.2; 8.6.1, 8.6.2.1 and 8.6.2.2).* The ongoing BGB-3111_BGB-A317_Study_001, in which both compounds are evaluated, did not show a proportionally increased toxicity, however, given that the number of RT patients treated with this combination in clinical trials is quite low and both agents are still in clinical development, several risk mitigation measures will be implemented in this study: An interim safety analysis with close monitoring of any toxicities will be established at the beginning of this trial. After three cycles, the GPI, the coordinating physician, one statistician and other members of the protocol committee will assess whether the toxicity profile of the trial is acceptable and whether recruitment is continued (see chapter on interim safety analysis) or additional safety precautions will be necessary. Moreover, regular and close monitoring of blood count is mandated within this study (see study assessment table) and pre-cautions like administration of G-CSF are implemented in the protocol.

Hence, in light of the currently available poor therapeutic options for RT patients and the suggested good tolerability of both investigational products, the benefit-risk ratio for this study is considered favorable.

3.2.1 Benefit-Risk Assessment regarding Covid-19 pandemic

In March 2020, the SARS-CO2 virus outbreak was classified as a pandemic by the WHO. The pandemic has an impact on the health system as well as clinical trials and study participants.
Therefore, the conduct of the CLL-RT1 study was assessed on a risk-based basis and the study management decided on measures which should ensure the safety of the study participants as best as possible and which ensure the validity of the data collected. Deviations from the study plan are tolerated when the study participants are e.g. in self-isolation / quarantine, the access to public places (including hospitals) is restricted due to the risk of the spread of infections, or the study staff is not fully available at the trial site.

This assessment is based on the current situation and may need readjustment to meet research challenges and ensure the rights, safety and wellbeing of study participants.

Risk-assessment

Only patients with relapsed Richter's transformation will be included in the CLL-RT1 study. Richter's transformation is an aggressive lymphoma that urgently requires treatment. It is associated with a very poor prognosis and left untreated Richter's transformation can quickly lead to death. Hence, postponing therapy is not an option for all patients with Richter's transformation. This applies to standard therapy in the situation with the SARS-COV2 pandemic as well as to study therapy. In the CLL-RT1 study, planned treatment consists of up to 6 induction cycles in which tislelizumab is given once per cycle and zanubrutinib is taken twice daily, followed by consolidation therapy. During consolidation therapy tislelizumab is administered once per cycle and zanubrutinib is again taken twice daily. Patients who respond to therapy can continue to be treated as part of maintenance therapy with the same therapy regimen until progression or an allogeneic stem cell transplant.

Patients with Richter's transformation are most commonly treated with intensive chemoimmunotherapy outside of clinical studies. The chemotherapy-free study treatment in the RT1 protocol has less immunosuppressive properties than chemoimmunotherapeutic regimens like R-CHOP which are frequently used for RT.

Patients with CLL and RT suffer from a compromised immune system, both from the disease and from the treatment. For this reason, the protocol and patient information already contain detailed information on how to draw attention to infections and how to treat them appropriately. In addition, infections CTC° III or higher have to be reported as "adverse events of special interest" throughout the duration of the study.
The study management regularly evaluates the safety data of the study. The early detection of safety signals minimizes the risk for the included patients in case COVID-19 infections will occur more frequently or if those will take an unusual course.

Decisions and measures taken

• Patients who are currently under therapy should, if possible, attend all of the planned visits, the administration of the antibody must take place at the trial site and cannot be delegated.

• The therapy should not be interrupted.

• The final restaging should be carried out as intended, since the response assessment is the primary endpoint of the study.

• The associated blood sample shipment to Cologne should also be performed due to the scientific importance of the accompanying scientific program.

•. Patients in the follow-up phase should attend, if possible, all planned visits.

Central laboratories

The central laboratories in Ulm and Cologne are not closed and staff is on its full capacity again, so results are sent with no delay. In case SARS-CO2 virus spread will be worse at a given timepoint and staff will be reduced, results may be sent with a delay.

SAE reporting

SAEs, all AESIs and AEPIs must be reported within the legal deadlines. In case of reduced staff capacities (e.g. illness due to Covid-19) and in the event that the TrialMaster cannot be used for documentation in the eCRF system, SAE / AEPI should be reported by telephone and /or e-mail. As the employees of the Study Office of the GCLLSG work also partly from home, the SAE forms can be scanned and sent by email to Study Office of the GCLLSG as well.

SAEs received by telephone or sent informally by e-mail are temporarily transferred to an SAE form by the safety staff at the Study Office of the GCLLSG and must be re-documented by the trial site as soon as possible.

Medical advice

Medical advice from the study physicians working in the Study Office of the GCLLSG is possible at any time. The GCLLSG telephone will also be manned. The inquiries are then forwarded. The sites should

address their inquiries preferably by email to the appropriate contact persons so that forwarding the mails to the study physicians is possible.

Data management

In case of reduced staff (e.g. illness due to Covid-19), delays in the process of data management at the Study Office of the GCLLSG might occur. This applies, for example, to answering non-medical questions and registering patients in the GCLLSG register.

Monitoring

On-site monitoring visits are currently possible again, although regularly with requirements (e.g. SARS-CO2 virus testing before entering site) and/or restrictions (e.g. visit time restrictions). No telephone monitoring are momentarily performed, only onsite-visits. In case a telephone-monitoring visit will occasionally performed due to elevated visit restrictions, the respective onsite-visit will be made up as soon as possible.

Recommendation for COVID-19 vaccination

Every patient who takes part in studies of the GCLLSG should be given the option of COVID vaccination using mRNA, vector or protein-based vaccines due to the increased COVID-19 associated mortality in the context of the haematological disease.

The time point of vaccination must be decided on a case-by-case basis by the treating physician and consultation of the GCLLSG study office is highly recommended. The sites should address their inquiries preferably by email to the appropriate contact persons so that they forward these to the study physicians.

Interrupting therapy should be avoided as far as possible!

Communication to the trial sites

The sites open in the CLL-RT1 study were informed about the measures taken in a newsletter. The sites will be informed about changes and new findings on a regular basis. Relevant information is also available on the GCLLSG homepage.

3.3. Primary objective

The primary objective of the study is to evaluate the efficacy of a combinational therapy with tislelizumab and zanubrutinib in CLL patients with Richter transformation to DLBCL. This will be based on the overall response rate (ORR) after induction therapy, (i.e. after 6 cycles) according to the refined Lugano Classification of 2016 [12].

3.4. Secondary and exploratory objectives

Further secondary endpoints to assess efficacy are: ORR after induction therapy according to IWCLL criteria (Hallek et al, 2018) [13], ORR after consolidation therapy (i.e. 12 cycles), progression-free survival (PFS), overall survival (OS), duration of response and time to next treatment (TTNT).

Furthermore, the safety of the regimen will be assessed by documentation of the type, frequency, and severity of adverse events (AEs) and their relationship to study treatment.

Exploratory objectives focus on the evaluation of the relationship between various baseline markers and

clinical outcome parameters. For instance, the response rates will be evaluated for different subgroups based on genetic aberrations like TP53 mutations, the expression of PD-1/PD-L1 or the mutational load.

4. Organizational and administrative aspects of the trial

4.1. Sponsor

University of Cologne, Albertus-Magnus-Platz, 50923 Cologne, Germany.

4.2. Chairman of the GCLLSG

Prof. Dr. med. Michael Hallek; Department I of Internal Medicine, Cologne University Hospital, Kerpener Strasse 62, 50937 Cologne, Germany.

4.3. Global Principal Investigator and Sponsor´s representative

Prof. Dr. med. Barbara Eichhorst (barbara.eichhorst@uk-koeln.de); Department I of Internal Medicine, Cologne University Hospital, Kerpener Strasse 62, 50937 Cologne, Germany.

4.4. Coordinating Physician

Dr. med. Othman Al-Sawaf; Department I of Internal Medicine, Cologne University Hospital; Kerpener Strasse 62, 50937 Cologne, Germany.

4.5. Head of the GCLLSG study office

Dr. med. Kirsten Fischer; Study Office of the German CLL Study Group, Department I of Internal Medicine, Cologne University Hospital; Kerpener Strasse 62, 50937 Cologne, Germany.

4.6. Statistics

Dr. Dipl.-Math. Sandra Robrecht; Study Office of the German CLL Study Group, Department I of Internal Medicine, Cologne University Hospital, Kerpener Str. 62, 50937 Cologne, Germany.

4.7. Project Management

Vanessa Beste (vanessa.beste@uk-koeln.de, 0221-478-96123), Dr. Laura Miesen (laura.miesen@uk-koeln.de, 0221-478-42564), Dr. Emily Holmes (emily.holmes@uk-koeln.de, 0221-478-96118), Study Office of the German CLL Study Group, Department I of Internal Medicine, Cologne University Hospital, Kerpener Str. 62, 50937 Cologne, Germany.

4.8. Safety management

Dr. med. Anna Fink (anna-maria.fink@uk-koeln.de), Sabine Frohs (sabine.frohs@uk-koeln.de, 0221-478-89621), Tanja Annolleck (tanja.annolleck@uk-koeln.de, 0221-478-96579) and Study Office of the German CLL Study Group, Department I of Internal Medicine, Cologne University Hospital, Kerpener Str. 62, 50937 Cologne, Germany.

For reporting of SAEs the eCRF (Trial Master) should be used. In case there is a problem with the eCRF the SAE can be sent paper based to the **fax-number 0221-478-86886** of the GCLLSG study office must be used, only for questions the above listed persons can be contacted directly or via the email **cll-safety@uk-koeln.de.** Please be aware that the paper based procedure only applies in emergencies and the site must later add the data to the eCRF.

4.9. Other trial management

Data Management and all other trial management (e.g. quality assurance) will also be executed by the GCLLSG study office (0221-478-88220; cll-studie@uk-koeln.de). Study Office of the German CLL Study Group, Department I of Internal Medicine, Cologne University Hospital, Kerpener Str. 62, 50937 Cologne, Germany.

4.10. Monitoring

Kompetenznetz Maligne Lymphome (KML) e.V. (Competence network malignant lymphomas), Geschäftsstelle, Cologne University Hospital, 50924 Cologne, Germany.

4.11. Investigators and trial sites

This clinical trial will be carried out as an open-label multicenter trial at approximately ten sites in Germany, one in Austria and one in Denmark. If necessary, further qualified trial sites may be recruited to the trial.

A list of trial sites involved, including information on the principal investigators, further investigators, and optionally on trial staff, will be kept in the TMF and continuously updated.

4.11.1. Requirements for investigators and trial sites

In this trial, sites with experience in the treatment of CLL as well as aggressive lymphoma were selected. Only physicians are allowed to be investigators in this trial.

4.12. Financing

The clinical trial is financially supported by the pharmaceutical company BeiGene, Ltd., who will also provide the study drugs zanubrutinib (BGB-3111) and tislelizumab (BGB-A317).

4.13. Data Monitoring Committee

A data monitoring committee is not planned for this trial. In order to oversee the safety of the patients in the trial the GPI will review all serious adverse events in this trial, but also the SUSAR reports of all other trials evaluating zanubrutinib and tislelizumab as single agents or in combination as well as all other available data from other clinical trials. Also, the progress of the trial, as well as the integrity and validity of the data collected, will be checked via monitoring, data management and medical review.

In addition, an interim safety review will be performed by the GPI, the coordinating physician ~~coordinating PI~~, one statistician and the safety management team of the GCLLSG three months after recruitment of the first six patients. This review will assess if the toxicity profile of the trial is acceptable and will decide if the recruitment may be continued without a fixed recruitment rate, if additional safety precautions and monitoring are needed or the trial will be prematurely stopped.

5. Central laboratory assessment

All tests performed in the context of the central laboratory assessments will be performed free of charge for the participating sites and investigators. The results of immunophenotyping, serum parameters, and routine genetic analyses will be sent to the sites and to the GCLLSG study office. Shipping costs will be reimbursed by GCLLSG.

Please note:

Shipping days are from **Monday** to **Thursday** (exceptions might occur during public holidays, please see the Lab Manual or contact the GCLLSG for further information). The samples should be sent to the appropriate laboratories together with the appropriate shipment forms **within 48 hours**.

Further information e.g. sample size, vials to be used and shipping addresses are listed in the *Appendix A: central laboratory assessment*.

5.1. Immunophenotyping

The immunophenotyping as central reference testing for confirmation of diagnosis will be performed at the time point of screening before inclusion to the trial in the hematological laboratory (LMHO) at the University Hospital in Cologne.

5.2. Serum parameters

The central reference testing of the two parameters serum beta-2-microglobulin (ß2MG) and thymidine kinase (TK), will be performed at screening at the central laboratory at Cologne University Hospital, samples will be shipped to the hematological laboratory and forwarded to the central laboratory.

5.3. Routine Genetic analyses

The central genetics reference testing includes fluorescence in situ hybridization (FISH) and mutation analysis of genes recurrently mutated in CLL (TP53, ATM, NOTCH1, SF3B1, BIRC3 etc.) by targeted resequencing and IGHV mutation status. These analyses will be performed at the GCLLSG reference laboratory at the University Hospital Ulm during screening and in the event of progression/relapse.

In addition, patients with prior treatment with ibrutinib or another BTK inhibitor (as single agent or in combination) will be tested for known mutations of BTK and Phospholipase C Gamma 2 (PLCg2), which are known to cause resistance. This analysis will also be performed at screening in the laboratory at Ulm University Hospital.

Furthermore, a karyotyping of the CLL cells will be performed at baseline by the hematological laboratory (LMHO) at Cologne University Hospital.

5.4. Tumor biopsies

Diagnosis of RT must be confirmed by lymph node biopsy and/or bone marrow biopsy. The written histopathological report has to be provided to the GCLLSG office in Cologne together with the Screening CRFs prior to treatment initiation. Furthermore, submission of formalin-fixed paraffin embedded tumor tissue sample blocks to Cologne is mandatory. If blocks cannot be shipped, 20 unstained slides should be freshly cut and submitted to the testing laboratory within 14 days from site slide section date, otherwise new specimen will be requested.

For patients who relapse or progress during or after study treatment, collection of a new lymph node biopsy is strongly encouraged. Paraffin blocks/unstained slides should be shipped to Cologne for further analyses.

The collected material will be used to centrally re-confirm diagnosis of RT and will be used for further

scientific analysis. Patients' written consent will be obtained prior to further scientific analysis of the biopsies.

5.5. Accompanying scientific program

Given the limited understanding of the causes and genetic basis of RT, within this trial characteristics of patients with malignant transformation and the impact of immunotherapy in the treatment of cancer will be analyzed systematically. In order to gain a deeper insight into the pathogenesis of RT, tumor biopsy material, which is routinely collected and mandatory for diagnosis of RT, will be analyzed in the accompanying scientific program. Moreover, regular sampling of peripheral blood will allow a longitudinal observation of the immune system during therapy and the impact of checkpoint inhibition and BTK inhibition on antitumor immunity. Extended targeted or genome-wide analyses using next-generation sequencing methods will be performed with patient samples obtained at study entry as well as upon disease progression. Alterations of the BCR pathway or of epigenetic signatures will be analyzed as well. These investigations will be performed by research laboratories at the University of Cologne and cooperation partners.

Patients will be asked to agree that parts of the provided material will be used for these scientific purposes in pseudonymized manner. Written consent will be obtained and stored in the patients' files.

5.6. Optional scientific analyses

All patients will be asked to agree that the leftovers of samples sent to Cologne and Ulm for the above described analyses may be stored and used for other scientific analyses and projects. However, the consent for the use of the leftovers and the sampling for the below two scientific projects is by choice and not a prerequisite for the participation in this clinical trial and will be covered by a separate transfer of ownership agreement.

6. Trial population

Eligible patients must fulfill the following inclusion/exclusion criteria:

Inclusion criteria

1. Confirmed diagnosis of CLL according to iwCLL criteria (Hallek et al, 2018) [13].
2. Confirmed histopathological diagnosis of RT (diffuse large B-cell lymphoma or Hodgkin's lymphoma [Hodgkin's lymphoma only when not eligible for more intensive treatment])
3. Previously untreated RT or patients with objective response or non-tolerance to first-line RT treatment
4. Creatinine clearance ≥30ml/min calculated according to the modified formula of Cockcroft and Gault or directly measured with 24hr urine collection or an equivalent method.
5. Adequate liver function as indicated by a total bilirubin≤ 2 x, AST/ALT ≤ 2.5 x the institutional ULN value, unless directly attributable to the patient's CLL or to Gilbert's Syndrome, in which case a max. total bilirubin ≤ 4 x and AST/ALT ≤ 5 x the institutional ULN value are required.[1]
6. Negative serological testing for hepatitis B (HBsAg negative and anti-HBc negative; patients positive for anti-HBc may be included if PCR for HBV DNA is negative and HBV-DNA PCR is performed every two months until 2 months after last dose of zanubrutinib), negative testing for hepatitis-C RNA and negative HIV test within 6 weeks prior to registration
7. Age at least 18 years
8. ECOG performance status 0-2, ECOG 3 is only permitted if related to CLL (e.g. due to anaemia or severe constitutional symptoms)
9. Life expectancy ≥ 3 months
10. Ability and willingness to provide written informed consent and to adhere to the study visit schedule and other protocol requirements

Exclusion criteria

1. Patients who did not respond to previous line of RT therapy (i.e. primary progressive patients)[2]
2. Patients with more than one prior line of RT therapy
3. Allogenic stem cell transplantation within the last 100 days or signs of active GVHD after prior allogeneic stem cell transplantation within any time

1 For patients who start study treatment with elevated liver enzymes due to CLL/RT or Gilbert's syndrome, toxicity and AE reporting will follow CTCAE grading once these values further increase. E.g. if a patient starts with a bilirubin value of 2.0 m/dl, which rises to 3.0 mg/dl after one cycle, this should be reported as grade 2 bilirubinemia (see CTCAE v5)

2 **Note**: In cases with urgent need for treatment, a prephase treatment with steroids, vincristine (up to 2 mg IV) or cyclophosphamide (up to 200 mg^2 daily for max 3 days) can be administered at the discretion of the treating physician prior to enrolment or start of study medication.

4. Patients with confirmed PML
5. Uncontrolled autoimmune condition
6. Malignancies other than CLL currently requiring systemic therapies (unless the malignant disease is in a stable remission at the discretion of the treating physician)
7. Active infection currently requiring systemic treatment
8. Any comorbidity or organ system impairment rated with a CIRS (cumulative illness rating scale) score of 4, excluding the eyes/ears/nose/throat/larynx organ system [1]or any other life-threatening illness, medical condition or organ system dysfunction that – in the investigator´s opinion could comprise the patient's safety or interfere with the absorption or metabolism of the study drugs
9. Requirement of therapy with strong CYP3A4 inhibitors/inducers
10. Requirement of therapy with phenprocoumon or other vitamin K antagonists.
11. Use of investigational agents, e.g. monoclonal antibodies or other experimental drugs within clinical trials, which might interfere with the study drug within 28 days (or 5 times half-life [$t_{1/2}$] of the compound, whichever is longer) prior to registration
12. Known hypersensitivity to tislelizumab, zanubrutinib or any of the excipients
13. Pregnant women and nursing mothers (a negative pregnancy test is required for all women of childbearing potential within 7 days before start of treatment)
14. Fertile men or women of childbearing potential unless:
    - surgically sterile or ≥ 2 years after the onset of menopause, or
    - willing to use two methods of reliable contraception including one highly effective contraceptive method (Pearl Index <1) and one additional effective (barrier) method during study treatment and for 12 months after the end of study treatment.
15. Vaccination with a live vaccine <28 days prior to randomization
16. Legal incapacity
17. Prisoners or subjects who are institutionalized by regulatory or court order
18. Persons who are in dependence to the sponsor or an investigator

1 This is to allow that patients who have sensory impairments, such as hardness of hearing plus impaired vision, can still be enrolled, despite 4 points on the CIRS scale. Infections of the upper respiratory tract should be recorded un-der the category "respiratory".

7. Trial conduct

7.1. Trial design

This trial is a phase-II study and – according to the definitions of the ICH Harmonized Tripartite Guideline E8 – a therapeutic exploratory type of study, which is designed as a prospective, multicenter, single-arm trial. The trial aims to investigate the efficacy and safety of a regimen consisting of 6 cycles of tislelizumab plus zanubrutinib as induction therapy, followed by 6 cycles of tislelizumab plus zanubrutinib as consolidation therapy. Patients with response to therapy continue treatment until disease progression. Patients with prior response and progression during treatment interruption may continue with therapy after consulting with the sponsor`s representative, GCLLSG study office.

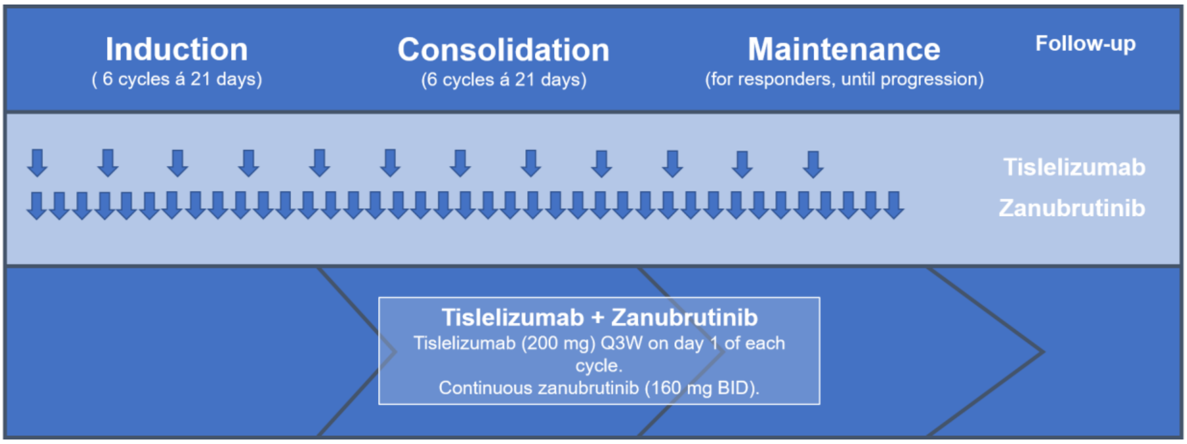

Figure 1 Treatment plan

7.1.1. Induction

Induction treatment consists of **6 cycles**, each with a duration of **21 days** (Q3W). Tislelizumab is administered intravenously on day 1 of each cycle. Continuous daily administration of zanubrutinib starts on day 1 of the first cycle as well.

Cycle 1-6:	Day 1:	Tislelizumab	200 mg iv
	QD:	Zanubrutinib	160 mg BID po

There have been infrequent reports of severe infusion-related reactions, characterized by systemic symptoms that start during or after the infusion. These include flushing, dyspnea, hypotension, chills, pruritus, and rash. For severe reactions, infusion should be stopped. For mild or moderate symptoms, the rate of infusion should be slowed by 50%.

Tislelizumab must be administered in a clinical setting (inpatient/ outpatient clinic/private practice). Patients should be under close supervision of the investigator at all times; resuscitation equipment and medications (including epinephrine for subcutaneous injections, corticosteroids, antihistamines for i.v. injection) should be available for immediate use.

On days with administration of zanubrutinib with tislelizumab, oral intake of zanubrutinib should be followed by intravenous administration of the antibody.

Co-administration of either a low-fat meal or a high-fat meal with zanubrutinib did not result in clinically relevant changes in exposure, thus zanubrutinib may be administered with or without food. Grapefruit juice and Seville oranges should be avoided, as they may affect the metabolism of zanubrutinib.

7.1.2. Consolidation

During consolidation, patients continue to receive both agents **over 6 cycles** (Q3W).

Cycle 7-12:	Day 1:	Tislelizumab	200 mg iv
	QD:	Zanubrutinib	160 mg BID po

7.1.3. Maintenance

Patients with response to therapy (i.e. CR, PR, and also SD) continue to take both agents until disease progression, non-tolerance or when receiving allogenic SCT for consolidation.

7.1.4. Treatment discontinuation

In case of unacceptable toxicities or disease progression during treatment, the treating physician may decide to stop maintenance treatment.

In case signs of disease progression occur during a phase of treatment interruption, study treatment may be resumed or continued after consultation of the GCLLSG study office, particularly in cases where alternative RT treatment options are not available.

Also, in case the patient achieves a response to therapy, the treating physician might at his own discretion, after consultation of the GCLLSG study office, consider referring the patient to allogenic transplantation. Treatment must be stopped prior to the beginning of any conditioning therapy.

7.1.5. Follow up

Patients will be followed up until 6 months after last study drug intake. To be able to collect long-term follow up data after the end of CLL-RT1 study, inclusion into the registry of the GCLLSG should be considered. For this purpose, each patient will be informed about the importance of long term follow data and asked for his/her consent to the long term follow-up within the GCLLSG registry. For patients with a written informed consent for the registry, data for overall survival, late toxicities such as secondary malignancies, further treatments and the course of the disease will be collected within the non-interventional GCLLSG registry after the end of the trial.

7.2. Interim safety analysis

The first six patients will be part of an interim safety analysis, for which a close site monitoring will be maintained in order to take into account SAEs and AESIs. The adverse events as specified below of the six patients in the safety cohort and the SAEs and AESIs of all patients included in the trial will be

analyzed. Special focus will be laid on:

- CTC° III/IV hematological toxicities related to study treatment, which require an intervention (e.g. additional monitoring, administration of G-CSF or blood transfusions),
- CTC° III/IV non-hematologic toxicities related to study treatment
- Serious laboratory syndromes,
- Serious cardiovascular and bleeding AEs, and
- AEs with a fatal outcome.

The results from the interim safety analysis and all available data (also from other clinical trials) regarding the drugs used in this trial will be reviewed by the GPI, the coordinating PI, one statistician and other members of the protocol committee. This review will determine if the recruitment can be continued and if additional safety precautions and monitoring are needed or the trial will be prematurely stopped.

7.3. Trial duration

Patient´s trial participation will include a screening phase, a treatment phase, and a follow-up phase. During the screening phase the central diagnostics, including an immunophenotyping for confirmation of CLL diagnosis and review of the histopathological report for confirmation of RT will be performed. A central medical review of the patient´s baseline characteristics and results from the central diagnostics will be performed in order to check the patient´s eligibility to the trial. After approval of the patient for inclusion to the trial, treatment should be initiated as soon as possible, without exceeding a 28 day period between time point of pretherapeutic staging and start of treatment.

Approximately 48 patients will be included into the trial; recruitment duration is approximately 2 years. Treatment consists of 6 induction cycles, each with a duration of 21 days, followed by 6 consolidation cycles. An interim staging after induction therapy as well as after consolidation therapy will be performed. Patients who maintain response to therapy after 12 cycles of therapy can continue treatment until disease progression or unacceptable toxicity or intolerability. Patients will be followed up until 6 months after last study drug intake. The end of the clinical trial is defined as the time point at which the last patient has completed his last follow up visit.

7.3.1. Planned study timelines

The following timelines are expected based on the expected recruitment rate (2 years expected recruitment phase) and maximum duration of treatment of the patients:

- Start of recruitment, i.e. first patient/first visit (FPFV): Q1/2020
- End of recruitment, i.e. last patient/first visit (LPFV): Q1/2023
- Expected end of study, i.e. last patient/last visit (LPLV): Q1/2024
- Final study report: Q1/2025

7.4. Registration and screening

The investigator assumes the responsibility of obtaining written informed consent for each subject before any study-specific procedures are performed.

After obtaining informed consent, all patients who meet the eligibility criteria for study entry can be enrolled into the trial. To verify the eligibility of patients, an internal medical review of the screening documents will be performed. Therefore, the registration form and completed CRF pages have to be provided to the GCLLSG study office. These will be reviewed by one of the GCLLSG study physicians together with the results of the baseline assessments in the central laboratories (especially immunophenotyping for confirmation of CLL diagnosis) and the written histopathological report (for confirmation of RT diagnosis) for verification of the eligibility of the patient. **In cases with a need for immediate treatment, the screening and approval process may be accelerated by sending the complete screening CRF pages together with local results from immunophenotyping for review to the GCLLSG study office; likewise, in cases with urgent treatment indication, results of molecular and cytogenetic examinations or radiographic reports may be handed in later, as long as the examinations have been performed per protocol.** Patients will be approved for trial participation as soon as possible (usually within a few hours) without the results of the central laboratories. However, samples for all central laboratory assessments need to be sent in for confirmation before initiation of treatment.

Please note: Approval of enrolment by the GCLLSG central study office is mandatory before initiation of study treatment; treatment should be initiated as soon as possible, without exceeding a 28 day period between time point of approval of the patient and start of treatment.

7.5. Recruitment strategy and stratification

For the statistical assumptions a CLL population composed solely of RT patients is assumed. To ensure 80 % power it will be necessary to enroll 48 patients. Patients will not be stratified.

7.5.1. Gender distribution

It is expected that more male patients will be included in the trial as men are affected more often by CLL than women (M:F ~1,9:1)[14]. Gender-specific differences will be compared descriptively as the trial is not powered for such comparisons.

7.5.2. Withdrawal

Investigators will make every reasonable effort to keep each patient in the study until all planned treatments and assessments have been performed. Patients have the right to withdraw from the study at any time for any reason.

Patients, who discontinue study treatment prematurely, will be followed up until they are considered end of study.

The investigator will inform the study office of the German CLL Study Group about the early withdrawal within seven days. At the time point of withdrawal, the investigators will make any reasonable effort to document all data available for the subject in the CRF and source documents including date and reasons of withdrawal.

7.5.3. End of study

All patients will be followed up until death or up to 6 months after last study drug intake. Afterwards the patients are considered end of study. Patients who withdraw their consent are also considered end of study.

7.5.4. End of trial

In contrast to the definition of end of study, the end of the clinical trial is defined as the time point once the last patient completed the last follow up visit.

7.6. GCLLSG Registry: Long-term follow up outside the CLL-RT1-trial

To be able to collect long term follow up data after the end of CLL-RT1 study, all patients should be included in the registry of the GCLLSG. For this purpose, each patient will be informed about the importance of long-term follow data and asked for his/her written informed consent to the long-term follow-up within the GCLLSG registry. For patients who agree to participate in the registry, data for overall survival, late toxicities such as secondary malignancies, further treatments and the course of the disease will be collected within the non-interventional GCLLSG registry after the end of the trial. However, participation in the CLL-RT1 trial is independent from the registry and is not mandatory for patients from the CLL-RT1 trial.

8. Study Medication

8.1. Ordering study medication

The pharmaceutical company BeiGene will provide the study drugs tislelizumab (BGB-A317) and zanubrutinib (BGB-3111). Sites will be provided with study medication via a central pharmacy.

The on-site pharmacy or clinical trial unit will receive tislelizumab and zanubrutinib and is responsible for the proper storage and drug accountability of all drugs and for the preparation of i.v. infusions for tislelizumab. Also, the on-site pharmacy is allowed to destroy unused trial medication in an appropriate way, after review by the site monitor. The receipt, drug accountability and destruction of all study medication has to be documented in the “Site inventory log”, which is filed in the investigator site file (ISF) at the site or in the pharmacy. Dispensing of tislelizumab and zanubrutinib trial medication to the patient has to be documented on the “Patient dispensing log”. The destruction of unused trial medication needs to be documented on the “Site inventory log” by the pharmacist or the site staff and a witness. Also, the loss of labeled trial medication has to be documented. In case the on-site pharmacy will not destroy unused study medication, this can be returned to BeiGene.

8.2. Tislelizumab (BGB-A317)

Tislelizumab (BGB-A317) will be distributed free of charge by BeiGene. For the most up to date information on tislelizumab please refer to the current version of the IB.

8.2.1. Concomitant medications

Information about clinical drug interactions with tislelizumab is not yet available.

The potential for drug-drug interaction between tislelizumab and small-molecule drug products is very low, given tislelizumab is a therapeutic monoclonal antibody. As tislelizumab is expected to be degraded into amino acids and recycle into other proteins, it is unlikely to have an effect on drug metabolizing enzymes or transporters.

8.2.2. Preparation of tislelizumab infusions

Tislelizumab is formulated as a sterile, nonpyrogenic, and isotonic injectable solution for IV administration in buffered formulation at pH 6.5, consisting of citrate, histidine, trehalose, polysorbate-20, and Water for Injection (WFI). It should be stored at 2°C to 8°C in the packaging boxes, protected from light until time to use. Tislelizumab is prepared for IV injection in a single-use vial containing a total of 100 mg antibody in 10 mL of buffered isotonic solution (i.e. 0.9% sodium chloride). The established flat dose for all patients is 200 mg of tislelizumab every three weeks.

Tislelizumab should be prepared by a healthcare professional using aseptic technique. From a microbiological point of view, the diluted product should be used immediately. If not used immediately, in use storage times and conditions prior to use are the responsibility of the user and should normally not be longer than 24 hours at 2 to 8 °C, unless dilution has taken place in controlled and validated aseptic conditions. For clinical batch-specific and formulation-specific instructions, and information on in-use stability, see the packaging label.

Unused tislelizumab vials and infusions can be either destroyed at the on-site pharmacy or returned to the vendor; unused tislelizumab cannot be re-used in this study or outside the trial.

8.2.3. Administration of tislelizumab

Interim analysis showed that the pharmacokinetic of tislelizumab was linear after IV infusion. The maximum serum concentration (Cmax) and AUC increased in a nearly dose-proportional manner from 0.5 mg/kg to 10 mg /kg. The mean terminal half-life (T1/2) estimated to be ~19 days and the steady state is expected to be reached in 76 days. Population PK analysis showed that patient body weight is not a significant covariate on the CL of tislelizumab, which supports flat-dosing of 200 mg Q3W.

Tislelizumab must be administered in a clinical setting (inpatient/outpatient clinic/private practice) after blood sampling and after intake of the oral study drugs (if applicable). Full emergency resuscitation facilities should be immediately available, and patients should be under supervision of the investigator at all times. Tislelizumab should be given as a slow i.v. infusion through an intravenous line with an infusion pump to control the infusion rate. Tislelizumab may not be administered as a bolus or i.v. push.

Pre-medication

For prevention of infusion related reactions, an antihistamine (eg, diphenhydramine or equivalent), antipyretic (eg, paracetamol or equivalent), and if considered indicated oral or IV glucocorticoids can be administered prior to the first infusion of tislelizumab.

Infusion rate

The initial infusion (Cycle 1, Day 1) will be delivered over 60 minutes; if this is well tolerated, then the subsequent infusions may be administered over 30 minutes, which is the shortest time period permissible for infusion. Tislelizumab must not be concurrently administered with any other drug.

8.2.3.1. Management of infusion-related reactions (IRRs)

Resuscitation equipment and appropriate medications (including epinephrine for s.c. injections, corticosteroids, antihistamines for i.v. injection) should be available for immediate use.

Treatment Modification for Symptoms of Infusion-Related Reactions Due to Tislelizumab

NCI-CTCAE Grade	Treatment Modification for Tislelizumab
**Grade 1 - mild**<br>Mild transient reaction; infusion interruption not indicated; intervention not indicated.	Decrease infusion rate by 50%. Any worsening is closely monitored. Medical management as needed.<br>Subsequent infusions should be given after premedication and at the reduced infusion rate.
**Grade 2 - moderate**<br>Therapy or infusion interruption indicated but responds promptly to symptomatic treatment (eg, antihistamines, NSAIDS, narcotics, IV fluids); prophylactic medications indicated for ≤ 24 h	Stop infusion. Infusion may be resumed at 50% of previous rate once infusion-related reactions has resolved or decreased to Grade 1 in severity. Any worsening is closely monitored. Proper medical management should be instituted as described below.<br>Subsequent infusions should be given after premedication and at the reduced infusion rate.
**Grade 3 – severe**<br>Prolonged (eg, not rapidly responsive to symptomatic medication and/or brief interruption of infusion); recurrence of symptoms following initial improvement; hospitalization indicated for clinical sequelae.	Immediately stop the infusion. Proper medical management should be instituted as described below.<br>The patient should be withdrawn from study drug(s) treatment.
**Grade 4 – life threatening**<br>Life-threatening consequences; urgent intervention indicated.	Immediately stop the infusion. Proper medical management should be instituted as described below.<br>The patient should be withdrawn from study drug(s) treatment.<br>Hospitalization is recommended.

Abbreviations: h, hours; IV, intravenous; NCI-CTCAE, National Cancer Institute Common Terminology Criteria for Adverse Event; NSAIDs, nonsteroidal anti-inflammatory drugs.

Once the tislelizumab infusion rate has been decreased by 50% or suspended due to an infusion related reaction, it must remain decreased for all subsequent infusions with premedication. If the patient has a second infusion-related reaction (≥ Grade 2) on the slower infusion rate, infusion should be discontinued and the patient should be withdrawn from tislelizumab treatment.

NCI-CTCAE Grade 1 or 2 infusion reaction: Proper medical management should be instituted, as indicated per the type of reaction. This includes but is not limited to an antihistamine (eg, diphenhydramine or equivalent), antipyretic (eg, paracetamol or equivalent), and if considered indicated oral or IV glucocorticoids, epinephrine, bronchodilators, and oxygen. In the next cycle, patients should receive oral premedication with an antihistamine (eg, diphenhydramine or equivalent) and an antipyretic (eg, paracetamol or equivalent), and they should be closely monitored for clinical signs and symptoms of an infusion reaction.

NCI-CTCAE Grade 3 or 4 infusion reaction: Proper medical management should be instituted immediately, as indicated per type and severity of the reaction. This includes but is not limited to oral or IV antihistamine, antipyretic, glucocorticoids, epinephrine, bronchodilators, and oxygen

It needs to be stressed that in the event of a life-threatening (which may include pulmonary or cardiac events), prolonged or recurrent IRR, tislelizumab should be discontinued immediately and no further tislelizumab should be administered. Patients experiencing IRRs CTC grade III or IV should receive aggressive symptomatic treatment and will be discontinued from further study treatment with tislelizumab. However, the treatment with zanubrutinib may be continued at the investigator´s discretion and the patient remains in the study.

8.3. Zanubrutinib (BGB-3111)

Zanubrutinib (BGB-3111) will be distributed free of charge by Beigene. For the most up to date information on zanubrutinib please refer to the current version of the IB.

The drug product consists of zanubrutinib drug substance filled into hard gelatin capsules as the active ingredient, as well as other excipients (microcrystalline cellulose as a diluent, croscarmellose sodium as a disintegrant, sodium lauryl sulfate as a wetting agent, colloidal silica as a fluid aid agent, and magnesium stearate as a lubricant). The drug product is provided at a dose strength of 80 mg, intended for oral use. It should be stored at room temperature (15°C – 30°C) in the provided high-density polyethylene bottles with induction seals. Bottles will contain 80 tablets.

Bottles with remaining tablets or empty bottles should be retrieved from the patients at the end of each dispensing interval and the date and number of returned tablets should be documented on the bottle and in the drug accountability records. All returned bottles should be retained for review of the drug accountability by the site monitor prior to destruction. Returned tablets cannot be re-used in this study or outside the study.

Bottles containing zanubrutinib have to be stored at controlled room temperature (i.e. 15 - 30°C) and must be maintained under adequate security and stored under the conditions specified on the label until dispensed for subject use or returned to the sponsor. Site personnel will instruct the patients how to store medication at home.

8.3.1. Administration of zanubrutinib

Zanubrutinib is rapidly absorbed and eliminated after oral administration in human subjects. The mean T1/2 was approximately 4 hours, and peak concentrations occurred around 2 hours postdose. The Cmax and the drug exposure (the AUC) increased in a nearly dose proportional manner from 40 mg to 320 mg, both after the single-dose and repeat-dose administrations. Food has minimal impact on PK of zanubrutinib. Human ADME studies indicated that zanubrutinib was primarily eliminated by hepatic metabolism and fecal excretion.

Based on available nonclinical metabolism data, zanubrutinib is primarily metabolized by CYP3A. **Avoid concomitant administration of zanubrutinib with strong CYP3A inhibitors.** For short-term use (treatment for 7 days or less) of strong CYP3A inhibitors (e.g., antifungals and antibiotics, see 8.4.2.1), consider interrupting zanubrutinib therapy until the CYP3A inhibitor is no longer needed. The GCLLSG study office should be consulted in these situations. Avoid concomitant administration of zanubrutinib with strong CYP3A inducers. Consider alternative agents with less CYP3A induction. Based on in vitro data, zanubrutinib is a moderate inhibitor of the human isoenzymes CYP2C8, CYP2C9, and CYP2C19. Drugs that are primarily metabolized by these isoenzymes should be used with caution when administering zanubrutinib, with monitoring of drug concentrations where appropriate.

Patients will receive the first dosage of zanubrutinib (day 1 of the first cycle) under medical supervision

in clinic/outpatient clinic/private practice. Patients will be advised to take all other doses of zanubrutinib at home.

Co-administration of either a low-fat meal or a high-fat meal with zanubrutinib did not result in clinically relevant changes in exposure, and the RP2D of 160 mg BID may be administered with or without food. **Zanubrutinib should be taken orally and swallowed whole with 250 ml water at breakfast and approximately 12 hours later, e.g. at dinner.** The capsules should not be opened or dissolved in water. Grapefruit juice and Seville oranges should be avoided, as they may affect the metabolism of zanubrutinib.

If vomiting occurs within 15 minutes after taking zanubrutinib and all expelled tablets are still intact, the intake may be repeated with the same dosage. Otherwise, no replacement dose is to be taken. If a dose is missed, it can be taken up to 3 hours after the scheduled time with a return to the normal schedule with the next dosage. If the delay is greater than 3 hours, the dose should be skipped and the patient should continue treatment the next scheduled dosage.

No dose escalation of study medication above 160mg zanubrutinib twice daily is allowed in this study.

Treatment with zanubrutinib should be temporarily held in case of adverse events (especially in case of adverse events of CTC grade III or higher that are deemed related to zanubrutinib. A permanent discontinuation may be necessary in case of unmanageable, potentially study drug-related toxicity as described in section 8.6.2.

8.4. Concomitant therapy

All concomitant therapies have to be documented on the respective CRF pages.

8.4.1. Permitted medications

Standard supportive care therapies (e.g. antiemetics, hydration G-CSF) needed for the management of symptoms are permitted, as clinically indicated.

8.4.1.1. Hematopoietic growth factors

Hematopoietic growth factors (e.g. G-CSF) may be administered during the entire study according to the American Society of Clinical Oncology (ASCO)[46], EORTC, and ESMO guidelines, namely in patients with advanced age, relevant comorbidities and in case of previous prolonged neutropenias and infections. The use of G-CSF is recommended for the treatment of neutropenia of CTC grade IV and especially in case of neutropenic infections.

8.4.1.2. Infectious prophylaxis

Prophylaxis of pneumocystis jirovecii-pneumonia is recommended, e.g. with cotrimoxazole (trimethoprim/sulfamethoxazole 160/800mg, either 1 tablet 3x/week [Mo/Wed/Fr, 1-0-0] or 2 tablets 2x/week [Mo/Thu, 2-0-0] or according to standard institutional practice). In case of not acceptable side effects or intolerances to cotrimoxazole or bone marrow insufficiency due to previous chemotherapy or other myelotoxicity, prophylactic use of an aerosolized formulation of pentamidine (inhalation by nebulizer once monthly) instead of cotrimoxazole should be considered.

Antiviral prophylaxis may be given at the discretion of the investigator. Particularly in patients with history of recurrent infections, e.g. Herpes simplex virus, prophylaxis with aciclovir is recommended. Because of drug interactions antifungal prophylaxis should be discussed with the GCLLSG office.

Prophylactic antibiotics, e.g. with chinolones, may be considered in all patients, in patients with multiple lines of previous therapy and in case of neutropenias CTC °III-IV with a duration >7 days.

However, caution should be taken regarding possible drug-drug-interactions with tislelizumab and za-nubrutinib, see below.

In case of hypogammaglobulinemia and repeated (urinary tract) infections, prophylactic substitution of intravenous immunoglobulins (IVIG) can be considered.

8.4.1.3. Medication and food to be used with caution during zanubrutinib treatment

8.4.1.4. CYP3A4 inhibitors/-inducers

Based on available nonclinical metabolism data, zanubrutinib is primarily metabolized by CYP3A and is a moderate inhibitor of CYP2C8, CYP2C9, and CYP2C19.

Avoid concomitant administration of zanubrutinib with strong CYP3A inhibitors. For short-term use (treatment for 7 days or less) of strong CYP3A inhibitors (e.g., antifungals and antibiotics), consider interrupting zanubrutinib therapy until the CYP3A inhibitor is no longer needed. The GCLLSG study office should be consulted in these situations.

Avoid concomitant administration of zanubrutinib with strong CYP3A inducers. Consider alternative agents with less CYP3A induction.

Based on in vitro data, zanubrutinib is a moderate inhibitor of the human isoenzymes CYP2C8, CYP2C9, and CYP2C19. Drugs that are primarily metabolized by these isoenzymes should be used with caution when administering zanubrutinib, with monitoring of drug concentrations where appropriate.

Pre-clinical and clinical investigations did not show any QT interval prolongation potential of za-nub-rutinib. The most relevant CYP3A4 inhibitors/inducers are listed below, a comprehensive list of P450 inhibitors, inducers and substrates can be found at **http://medicine.iupui.edu/clinpharm/ddis/main-table/**. This website is continually revised and should be checked frequently for updates.

Strong CYP3A4 inhibitors should be **avoided**, but may be used after consulting the GCLLSG study office if the benefit outweighs the risk and no alternative treatment options are available.

Examples of strong CYP3A4 inhibitors are:

- clarithromycin[1]
- fluconazole, ketoconazole and itraconazole
- ritonavir (Norvir®, Kaletra®), indinavir, saquinavir and nelfinavir

Moderate CYP3A4/5 inhibitors should be **avoided**, but may be used after consulting the GCLLSG if the benefit outweighs the risk and no alternative treatment options are available.

Examples of moderate CYP3A4 inhibitors are:

- ciprofloxacin and erythromycin
- voriconazole (VFEND®) and fluconazole (Diflucan®)
- aprepitant (Emend®)
- amiodaron
- diltiazem and verapamil [calcium channel blockers]
- dronedaron (Multaq®) [class III antiarrhythmic]

1) The use of Azithromycin should be considered in case of upper respiratory tract or other infections, as this macrolide antibi-otic does not inhibit CYP3A4.

- darunavir (Prezista®), (fos)amprenavir and atazanavir
- crizotinib (Xalkori®)[1] and imatinib (Glivec®)[2]
- grapefruit and seville oranges

Weak CYP3A4/5 inhibitors, e.g. cimetidine (H2 antagonist) can be used with caution.

Other known CYP3A4/5 inhibitors are:

- norfloxacin, chloramphenicol, trolenandomycin
- boceprevir, delaviridine, telaprevir
- fluoxetine (metabolite norfluoxetine), fluvoxamine [SSRIs]
- diethyl-dithiocarbamate
- gestodene
- mifepristone
- mibefradil [calcium channel blockers]
- star fruit.

Strong and moderate CYP3A4/5 inducers should be **avoided** because they can potentially decrease the plasma concentration of tislelizumab and zanubrutinib and put the patient at risk of an inefficient treatment and development of resistance.

Examples of **CYP3A4/5 inducers** include:

- glucocorticoids
- pioglitazone (Actos®), troglitazone
- efavirenz (Sustiva®, Atripla®), nevirapine (Viramune®)
- rifabutin, rifampin
- modafinil
- St. John´s wort
- carbamazepine (Tegretal®), oxcarbazepine (Trileptal®)
- phenytoine
- barbiturates, phenobarbital (Luminal®).

Because of potential interactions due to CYP3A4 inhibition, the consumption of the following food should be avoided:

- Seville oranges (often contained in bitter-orange marmalade and candied orange peel)
- grapefruit (including juice and other products containing grapefruit)
- star fruit.

Patients should be **strongly cautioned against excessive consumption of grapefruit or Seville oranges** (which contain potent CYP3A inhibitors), but also against using **herbal remedies or dietary supplements (in particular those containing St. John's wort**, which is a potent CYP3A inducer).

1) As these two agents are anticancer therapies, they are not to be used during the whole study treatment anyways.

If any of these agents will be used, the following dose modifications should be considered:

CYP3A	Co-administered Drug	Recommended use
Inhibition	Strong CYP3A inhibitor (eg. Ketoconazole, conivaptan, clarithromycin, indinavir, itraconazole, lopinavir, ritonavir, telaprevir, posaconazole, voriconazole)	80 mg once daily
	Moderate CYP3A inhibitor (eg. Erythromycin, ciprofloxacin, diltiazem, dronedarone, fluconazole, verapamil, aprepitant, imatinib, grapefruit products)	80 mg twice daily
Induction	Strong CYP3A inducer (eg. Carbamazepine, phenytoin, rifampin, St. John's wort)	Avoid concomitant use; Consider alternative agents with less induction potential.
	Moderate CYP3A inducer (eg. Bosentan, efavirenz, etravirine, modafinil, nafcillin)	160 mg twice daily, use with caution; Monitor for potential lack of efficacy.

8.4.1.5. Anticoagulants

Patients receiving treatment with phenprocoumon or other vitamin K antagonists are excluded from trial participation (see exclusion criterion no. 10) due to the limited experience with the concomitant administration of these agents with zanubrutinib and due a potentially increased risk of bleeding. However, switching the anticoagulant to heparine or novel anticoagulants, e.g. the oral direct Xa inhibitors rivaroxaban (Xarelto®), apixaban (Eliquis®) and edoxaban, as well as the direct thrombin inhibitor dabigatran (Pradaxa®) is permitted.

In patients requiring the initiation of anticoagulation during the course of the study, risk and benefit of continuation of study treatment and choice of the anticoagulant should be weighted carefully and possibly also discussed with the patient. Dabigatran (Pradaxa®) might be favoured over the direct Xa inhibitors as it is not metabolized via CYP3A4/5 and because with the antibody idarucizumab (Praxabind®), an "antidote" is available. However, fewer major bleeding events have been observed with apixaban as compared to rivaroxaban and dabigatran [47].

During co-administration heparin, direct Xa or thrombin inhibitors, vitamin k antagonists or antiplatelet agents together with zanubrutinib, patients should be observed closely for signs and symptoms of bleeding and the coagulation should be monitored carefully. In case of severe bleeding events, zanubrutinib should be withheld, however, no preemptive dose reduction zanubrutinib should be performed.

8.4.2. Surgery

Due to a potentially increased risk of bleeding, especially when used in combination with anticoagulants or antiplatelet agents, withholding zanubrutinib should be considered at least three days before and after surgical interventions.

8.5. Prohibited medication during entire study

The following medications are prohibited throughout the whole duration of the study treatment:

- any other chemotherapy, anticancer immunotherapy
- use of investigational agents

- continuous use of corticosteroids (at dosages ≥20mg/day prednisone or equivalent)
- immunization with a live vaccine

As the safety of immunization with live or attenuated vaccines (e.g. varicella, or measles, mumps, and rubella) during treatment with tislelizumab and zanubrutinib has not been studied, these vaccinations are prohibited during study treatment and cannot be recommended until B-cell recovery due to the potential risk of infection.

Also, no data exist regarding the safety and efficacy of other vaccinations (with inactivated vaccines, toxoids and conjugate vaccines) during antibody treatment; though the ability to generate an immune response may be impaired, these vaccines may be used if at the treating physician´s discretion.

8.6. Dose and schedule modifications

According to the assessment table, complete blood counts and analyses of serum chemistry as well as evaluations for (serious) adverse events will be performed; depending on the results of these assessments treatment should be held or postponed.

8.6.1. Dose and schedule modifications for tislelizumab

Tislelizumab should be held in case of:

- Febrile or active infection[1]
- any CTC °III/IV Aes (except hematological Aes that are due to bone marrow involvement of the CLL and/or RT)
- severe and life-threating immune-related adverse events associated with tislelizumab (see below)
  - hepatitis (AST / ALT elevation and/or increased bilirubin)
  - colitis / diarrhea
  - pneumonitis
  - hypo/hyperthyroidism
  - nephritis / renal dysfunction
  - myocarditis

In case of these Aes, patients should be monitored at least weekly until the event resolves. Administration of tislelizumab can be postponed for a maximum of six weeks, if the event doesn´t resolve within six weeks the investigator can decide to continue treatment with tislelizumab after consultation of the GCLLSG study office. No dose modification will be performed for tislelizumab.

8.6.1.1. Management of immune-related Aes caused by tislelizumab

Immune-related Aes are of special interest in this study. If the events listed below or similar events occur, the investigator should exclude alternative explanations (eg, combination drugs, infectious disease, metabolic, toxin, disease progression or other neoplastic causes) with appropriate diagnostic tests, which may include but are not limited to serologic, immunologic, and histologic (biopsy) data.

A list of potential irAEs is shown below. All conditions similar to those listed should be evaluated in patients receiving tislelizumab to determine whether they are immune-related.

1 If the infection is manageable and the patient is at risk of developing PD during treatment interruption, study treatment may be resumed or continued after consultation of the GCLLSG study office.

Recommendation for diagnostic evaluation and management of irAEs is based on European Society for Medical Oncology (ESMO) and American Society of Clinical Oncology (ASCO) guidelines (Haanen et al 2017, Brahmer et al 2018).

Recommended Diagnostic Tests in the Management of Possible Immune-related Adverse Events

**Immune-related Toxicity**	**Diagnostic Evaluation Guideline**
Thyroid Disorders	Scheduled and repeat thyroid function tests (TSH and T4).
Hypophysitis	Check visual fields and consider pituitary endocrine axis blood profile. Perform pituitary and whole brain MRI in patients with headache, visual disturbance, unexplained fatigue, asthenia, weight loss and unexplained constitutional symptoms.<br><br>Consider consultation with an endocrinologist if an abnormality is detected.
Pneumonitis	All patients presenting with new or worsened pulmonary symptoms or signs, such as an upper respiratory infection, new cough, shortness of breath or hypoxia should be assessed by high-resolution CT. Consider pulmonary function test including *D*LCO.<br><br>Radiographic appearance is often nonspecific. Depending on the location of the abnormality, bronchoscopy and bronchoalveolar lavage or lung biopsy may be considered. Consult with a respiratory medicine physician for cases of uncertain cause.
Neurological Toxicity	Perform a comprehensive neurological examination and brain MRI for all CNS symptoms; review alcohol history and other medications. Conduct a diabetic screen, and assess blood B12/folate, HIV status, TFTs, and consider autoimmune serology. Consider the need for brain/spine MRI/MRA and nerve conduction study for peripheral neuropathy. Consult with a neurologist if there are abnormal findings.
Colitis	Review dietary intake and exclude steatorrhea. Consider comprehensive testing, including the following: FBC, UEC, LFTs, CRP, TFTs, stool microscopy and culture, viral PCR, Clostridium difficile toxin, cryptosporidia (drug-resistant organism).<br><br>In case of abdominal discomfort, consider imaging, eg, X-ray, CT scan. If a patient experiences bleeding, pain or distension, consider colonoscopy with biopsy and surgical intervention, as appropriate.
Eye Disorders	If a patient experiences acute, new onset, or worsening of eye inflammation, blurred vision, or other visual disturbances, refer the patient urgently to an ophthalmologist for evaluation and management.

Recommended Diagnostic Tests in the Management of Possible Immune-related Adverse Events

Immune-related Toxicity	Diagnostic Evaluation Guideline
Hepatitis	Check ALT/AST/total bilirubin, INR/albumin; the frequency will depend on severity of the AE (eg, daily if Grade 3-4; every 2-3 days if Grade 2, until recovering). Review medications (eg, statins, antibiotics) and alcohol history. Perform liver screen including Hepatitis A/B/C serology, Hepatitis E PCR and assess anti-ANA/SMA/LKM/SLA/LP/LCI, iron studies. Consider imaging, eg, ultrasound scan for metastases or thromboembolism. Consult with a hepatologist and consider liver biopsy.
Renal toxicity	Review hydration status and medication history. Test and culture urine. Consider renal ultrasound scan, protein assessment (dipstick/24-hour urine collection), or phase-contrast microscopy. Refer to nephrology for further management assistance.
Dermatology	Consider other causes by conducting a physical examination, consider dermatology referral for skin biopsy.
Joint or muscle inflammation	Conduct musculoskeletal history and perform complete musculoskeletal examination. Consider joint X-ray and other imaging as required to exclude metastatic disease. Perform autoimmune serology and refer to rheumatology for further management assistance.<br>For suspected myositis/rhabdomyolysis/myasthenia include: CK, ESR, CRP, troponin and consider a muscle biopsy.
Myocarditis	Perform ECG, echocardiogram, CK/CK-MB, troponin (I and/or T), and refer to a cardiologist.

Abbreviations: AE, adverse event; ALT, alanine aminotransferase; ANA, antinuclear antibody; AST, aspartate aminotransferase; CK, creatinine kinase; CK-MB, creatinine kinase cardiac isoenzyme; CNS, central nervous system; CRP, C-reactive protein; CT, computed tomography; *D*LCO, diffusing capacity for carbon monoxide; ECG, electrocardiogram; ESR, erythrocyte sedimentation rate; FBC, full blood count; HIV, human immunodeficiency virus; INR, international normalized ratio; LCI, liver cytosolic antigen; LFT, liver function test; LKM, liver kidney microsomal antibody; LP, liver pancreas antigen; MRA, magnetic resonance angiogram; MRI, magnetic resonance imaging; PCR, polymerase chain reaction; SLA, soluble liver antigen; SMA, smooth muscle antibody; T4, thyroxine; TFT, thyroid function tests; TSH, thyroid-stimulating hormone; UEC, urea electrolytes and creatinine.

Treatment of Immune-Related Adverse Events

- Immune-related Aes can escalate quickly; study treatment interruption, close monitoring, timely diagnostic work-up and treatment intervention, as appropriate, with patients is required
- Immune-related Aes should improve promptly after introduction of immunosuppressive therapy. If this does not occur, review the diagnosis, seek further specialist advice and contact the study medical monitor

- For some Grade 3 toxicities that resolve quickly, rechallenge with study drug may be considered if there is evidence of a clinical response to study treatment, after consultation with the GCLLSG study office
- Steroid dosages in the table below are for oral or intravenous (methyl)prednisolone. Equivalent dosages of other corticosteroids can be substituted. For steroid-refractory irAEs, consider use of steroid-sparing agents (eg, mycophenolate mofetil [MMF])
- Consider prophylactic antibiotics for opportunistic infections if the patient is receiving long-term immunosuppressive therapy

**Autoimmune Toxicity**	**Grade**	**Treatment Guidelines (Subject to Clinical Judgement)**	**Study Drug Management**
**Thyroid Disorders**	**1-2**<br>Asymptomatic TFT abnormality or mild symptoms	Replace thyroxine if hypothyroid, until TSH/T4 levels return to normal range.<br>Thyrotoxic patients should be referred to an endocrinologist. In cases with systemic symptoms: withhold study treatment, treat with a beta blocker and consider oral prednisolone 0.5 mg/kg/day for thyroid pain. Taper corticosteroids over 2-4 weeks. Monitor thyroid function regarding the need for hormone replacement.	Continue study treatment or withhold treatment in cases with systemic symptoms.
	**3-4**<br>Severe symptoms, hospitalization required	Refer patient to an endocrinologist.<br>If hypothyroid, replace with thyroxine 0.5-1.6 µg/kg/day (for the elderly or those with co-morbidities, the suggested starting dose is 0.5 µg/kg/day). Add oral prednisolone 0.5 mg/kg/day for thyroid pain. Thyrotoxic patients require treatment with a beta blocker and may require carbimazole until thyroiditis resolves.	Hold study treatment; resume when resolved/improved to Grade 0-1.

Autoimmune Toxicity	Grade	Treatment Guidelines (Subject to Clinical Judgement)	Study Drug Management
**Hypophysitis**	**1-2**<br><br>Mild-moderate symptoms	Refer patient to an endocrinologist for hormone replacement. Add oral prednisolone 0.5-1 mg/kg/day for patients with pituitary inflammation. Taper corticosteroids over at least 1 month. If there is no improvement in 48 hours, treat as Grade 3-4. Taper corticosteroids over at least 1 month.	Continue study treatment.
	**3-4**<br><br>Severe or life-threatening symptoms	Refer patient to an endocrinologist for assessment and treatment. Initiate pulse IV methylprednisolone 1 mg/kg for patients with headache/visual disturbance due to pituitary inflammation. Convert to oral prednisolone and taper over at least 1 month. Maintain hormone replacement according to endocrinology advice. Maintain hormone replacement according to endocrinology advice.	Hold study treatment for patients with headache/visual disturbance due to pituitary inflammation until resolved/improved to Grade 2 or less. Discontinuation is usually not necessary.
**Pneumonitis**	**1**<br><br>Radiographic changes only	Monitor symptoms every 2-3 days.<br><br>If appearance worsens, treat as Grade 2.	Consider holding study treatment until appearance improves and cause is determined.
	**2**<br><br>Symptomatic: exertional breathlessness	Commence antibiotics if infection suspected. Add oral prednisolone 1 mg/kg/day if symptoms/appearance persist for 48 hours or worsen. Consider Pneumocystis infection prophylaxis. Taper corticosteroids over at least 6 weeks.<br><br>Consider prophylaxis for adverse steroid effects: eg, blood glucose monitoring, vitamin D/calcium supplement.	Hold study treatment. Retreatment is acceptable if symptoms resolve completely or are controlled on prednisolone ≤ 10 mg/day. Discontinue study treatment if symptoms persist with corticosteroid treatment.

Autoimmune Toxicity	Grade	Treatment Guidelines (Subject to Clinical Judgement)	Study Drug Management
	**3-4**<br>Severe or life-threatening symptoms<br>Breathless at rest	Admit to a hospital and initiate treatment with IV methylprednisolone 2-4 mg/kg/day. If there is no improvement, or worsening after 48 hours, add infliximab 5 mg/kg (if no hepatic involvement). Convert to oral prednisolone and taper over at least 2 months. Cover with empiric antibiotics and consider prophylaxis for Pneumocystis infection and other adverse steroid effects, eg, blood glucose monitoring, vitamin D/calcium supplement.	Discontinue study treatment.
**Neurological Toxicity**	**1**<br>Mild symptoms	–	Continue study treatment.
	**2**<br>Moderate symptoms	Treat with oral prednisolone 0.5-1 mg/kg/day. Taper over at least 4 weeks. Obtain neurology consultation.	Hold study treatment; resume when resolved/improved to Grade 0-1.
	**3-4**<br>Severe/life-threatening	Initiate treatment with oral prednisolone or IV methylprednisolone 1-2 mg/kg/day, depending on symptoms. Taper corticosteroids over at least 4 weeks.<br>Consider azathioprine, MMF, cyclosporine if no response within 72-96 hours.	Discontinue study treatment.
**Colitis/Diarrhea**	**1**<br>Mild symptoms: < 3 liquid stools per day over baseline and feeling well	Symptomatic management: fluids, loperamide, avoid high fiber/lactose diet.<br>If Grade 1 persists for > 14 days manage as a Grade 2 event.	Continue study treatment.

<table>
<tr><th>Autoimmune Toxicity</th><th>Grade</th><th>Treatment Guidelines (Subject to Clinical Judgement)</th><th>Study Drug Management</th></tr>
<tr><td rowspan="3"></td><td>2

Moderate symptoms: 4-6 liquid stools per day over baseline, or abdominal pain, or blood in stool, or nausea, or nocturnal episodes</td><td>Oral prednisolone 0.5 mg/kg/day (non-enteric coated).

Do not wait for any diagnostic tests to start treatment. Taper steroids over 2-4 weeks, consider endoscopy if symptoms are recurring.</td><td>Hold study treatment; resume when resolved/improved to baseline grade.</td></tr>
<tr><td>3

Severe symptoms: > 6 liquid stools per day over baseline, or if episodic within 1 hour of eating</td><td rowspan="2">Initiate IV methylprednisolone 1-2 mg/kg/day.

Convert to oral prednisolone and taper over at least 4 weeks. Consider prophylaxis for adverse steroid effects, eg, blood glucose monitoring, vitamin D/calcium supplement.

If no improvement in 72 hours or symptoms worsen, consider infliximab 5 mg/kg if no perforation, sepsis, TB, hepatitis, NYHA grade III/IV CHF or other immunosuppressive treatment: MMF or tacrolimus.

Consult gastroenterologist to conduct colonoscopy/ sigmoidoscopy.</td><td>Hold study treatment; retreatment may be considered when resolved/improved to baseline grade and after discussion with the study medical monitor.</td></tr>
<tr><td>4

Life-threatening symptoms</td><td>Discontinue study treatment.</td></tr>
<tr><td rowspan="2">Skin reactions</td><td>1

Skin rash, with or without symptoms, < 10% BSA</td><td>Avoid skin irritants and sun exposure; topical emollients recommended.</td><td>Continue study treatment.</td></tr>
<tr><td>2

Rash covers 10%-30% of BSA</td><td>Avoid skin irritants and sun exposure; topical emollients recommended.

Topical steroids (moderate strength cream once a day or potent cream twice a day) ± oral or topical antihistamines for itch. Consider a short course of oral steroids.</td><td>Continue study treatment.</td></tr>
</table>

Autoimmune Toxicity	Grade	Treatment Guidelines (Subject to Clinical Judgement)	Study Drug Management
	**3**<br><br>Rash covers > 30% BSA or Grade 2 with substantial symptoms	Avoid skin irritants and sun exposure; topical emollients recommended.<br><br>Initiate steroids as follows based on clinical judgement:<br><br>For moderate symptoms: oral prednisolone 0.5-1 mg/kg/day for 3 days then taper over 2-4 weeks.<br><br>For severe symptoms: IV methylprednisolone 0.5-1 mg/kg/day; convert to oral prednisolone and taper over at least 4 weeks.	Hold study treatment.<br><br>Re-treat when AE is resolved or improved to mild rash (Grade 1-2) after discussion with the study medical monitor.
	**4**<br><br>Skin sloughing > 30% BSA with associated symptoms (eg, erythema, purpura, epidermal detachment)	Initiate IV methylprednisolone 1-2 mg/kg/day. Convert to oral prednisolone and taper over at least 4 weeks.<br><br>Admit to a hospital and seek urgent dermatology consultation.	Discontinue study treatment.
**Hepatitis**	**1**<br><br>ALT or AST > ULN to 3X ULN	Check LFTs within 1 week and before the next dose check LFTs to verify that there has been no worsening.<br><br>If LFTs are worsening, recheck every 48-72 hours until improvement is seen.	Continue study treatment if LFTs are unchanged or improving.<br><br>Hold study treatment if LFTs are worsening until improvement is seen.
	**2**<br><br>ALT or AST 3-5X ULN	Recheck LFTs every 48-72 hours:<br><br>For persistent ALT/AST elevation: consider oral prednisolone 0.5-1 mg/kg/day for 3 days then taper over 2-4 weeks.<br><br>For rising ALT/AST: start oral prednisolone 1 mg/kg/day and taper over 2-4 weeks; re-escalate dose if LFTs worsen, depending on clinical judgement.	Hold study treatment; treatment may be resumed when resolved/improved to baseline Grade and prednisolone tapered to ≤ 10 mg.

Autoimmune Toxicity	Grade	Treatment Guidelines (Subject to Clinical Judgement)	Study Drug Management
	**3**<br><br>ALT or AST 5-20X ULN	ALT/AST < 400 IU/L and normal bilirubin/INR/albumin: Initiate oral prednisolone 1 mg/kg and taper over at least 4 weeks.<br><br>ALT/AST > 400 IU/L or raised bilirubin/INR/low albumin: Initiate IV (methyl)prednisolone 2 mg/kg/day. When LFTs improve to Grade 2 or lower, convert to oral prednisolone and taper over at least 4 weeks.	Hold study treatment until improved to baseline Grade; reintroduce only after discussion with the study medical monitor.
	**4**<br><br>ALT or AST > 20X ULN	Initiate IV methylprednisolone 2 mg/kg/day. Convert to oral prednisolone and taper over at least 6 weeks.	Discontinue study treatment.
	**Worsening LFTs despite steroids:**<br><br>• If on oral prednisolone, change to pulsed IV methylprednisolone<br>• If on IV, add mycophenolate mofetil (MMF) 500-1000 mg twice a day<br>• If worsens on MMF, consider addition of tacrolimus<br><br>Duration and dose of steroid required will depend on severity of event		
**Nephritis**	**1**<br><br>Creatinine 1.5X baseline<br><br>or > ULN to 1.5X ULN	Repeat creatinine weekly.<br><br>If symptoms worsen, manage as per criteria below.	Continue study treatment.
	**2**<br><br>Creatinine > 1.5X-3X baseline or > 1.5X-3X ULN	Ensure hydration and review creatinine in 48-72 hours; if not improving, consider creatinine clearance measurement by 24-hour urine collection. Discuss with nephrologist the need for kidney biopsy.<br><br>If attributed to study drug, initiate oral prednisolone 0.5-1 mg/kg and taper over at least 2 weeks.<br><br>Repeat creatinine/U&E every 48-72 hours.	Hold study treatment.<br><br>If not attributed to drug toxicity, restart treatment.<br><br>If attributed to study drug and resolved/improved to baseline grade: Restart study drug if tapered to < 10 mg prednisolone.

Autoimmune Toxicity	Grade	Treatment Guidelines (Subject to Clinical Judgement)	Study Drug Management
	**3**<br>Creatinine > 3X baseline or > 3X-6X ULNf	Hospitalize patient for monitoring and fluid balance; repeat creatinine every 24 hours; refer to a nephrologist and discuss need for biopsy. If worsening, initiate IV (methyl)prednisolone 1-2 mg/kg. Taper corticosteroids over at least 4 weeks.	Hold study treatment until the cause is investigated.<br>If study drug suspected: Discontinue study treatment.
	**4**<br>Creatinine > 6X ULN	As per Grade 3, patient should be managed in a hospital where renal replacement therapy is available.	Discontinue study treatment.
**Diabetes/ Hyperglycemia**	**1**<br>Fasting glucose value ULN to 160 mg/dL; ULN to 8.9 mmol/L	Monitor closely and treat according to local guideline. Check for C-peptide and antibodies against glutamic acid decarboxylase and islet cells are recommended	Continue study treatment.
	**2**<br>Fasting glucose value 160-250 mg/dL; 8.9-13.9 mmol/L	Obtain a repeat blood glucose level at least every week. Manage according to local guideline.	Continue study treatment or hold treatment if hyperglycemia is worsening. Resume treatment when blood glucose is stabilized at baseline or Grade 0-1.
	**3**<br>Fasting glucose value 250-500 mg/dL; 13.9-27.8 mmol/L	Admit patient to hospital and refer to a diabetologist for hyperglycemia management. Corticosteroids may exacerbate hyperglycemia and should be avoided.	Hold study treatment until patient is hyperglycemia symptom-free, and blood glucose has been stabilized at baseline or Grade 0-1.
	**4**<br>Fasting glucose value > 500 mg/dL; > 27.8 mmol/L	Admit patient to hospital and institute local emergency diabetes management. Refer the patient to a diabetologist for insulin maintenance and monitoring.	
**Ocular Toxicity**	**1**<br>Asymptomatic eye exam/test abnormality	Consider alternative causes and prescribe topical treatment as required.	Continue study treatment.

Autoimmune Toxicity	Grade	Treatment Guidelines (Subject to Clinical Judgement)	Study Drug Management
	**2**<br><br>Anterior uveitis or mild symptoms	Refer patient to an ophthalmologist for assessment and topical corticosteroid treatment. Consider a course of oral steroids.	Continue study treatment or hold treatment if symptoms worsen or if there are symptoms of visual disturbance.
	**3**<br><br>Posterior uveitis/ panuveitis or significant symptoms	Refer patient urgently to an ophthalmologist. Initiate oral prednisolone 1-2 mg/kg and taper over at least 4 weeks.	Hold study treatment until improved to Grade 0-1; reintroduce only after discussion with the study medical monitor.
	**4**<br><br>Blindness (at least 20/200) in the affected eyes	Initiate IV (methyl)prednisolone 2 mg/kg/day. Convert to oral prednisolone and taper over at least 4 weeks.	Discontinue study treatment.
**Pancreatitis**	**2**<br><br>Asymptomatic, blood test abnormalities	Monitor pancreatic enzymes.	Continue study treatment.
	**3**<br><br>Abdominal pain, nausea and vomiting	Admit to hospital for urgent management. Initiate IV (methyl)prednisolone 1-2 mg/kg/day. Convert to oral prednisolone when amylase/lipase improved to Grade 2, and taper over at least 4 weeks.	Hold study treatment; reintroduce only after discussion with the study medical monitor.
	**4**<br><br>Acute abdominal pain, surgical emergency	Admit to hospital for emergency management and appropriate referral.	Discontinue study treatment.
**Arthritis**	**1**<br><br>Mild pain with inflammation, swelling	Management per local guideline.	Continue study treatment.
	**2**<br><br>Moderate pain with inflammation, swelling, limited instrumental (fine	Management as per local guideline. Consider referring patient to a rheumatologist. If symptoms worsen on treatment manage as a Grade 3 event.	Continue treatment or, if symptoms continue worsens, hold study

Autoimmune Toxicity	Grade	Treatment Guidelines (Subject to Clinical Judgement)	Study Drug Management
	motor) activities		treatment until symptoms improve to baseline or Grade 0-1.
	**3**<br>Severe pain with inflammation or permanent joint damage, daily living activity limited	Refer patient urgently to a rheumatologist for assessment and management. Initiate oral prednisolone 0.5-1 mg/kg and taper over at least 4 weeks.	Hold study treatment unless improved to Grade 0-1; reintroduce only after discussion with the study medical monitor.
**Mucositis/ stomatitis**	**1**<br>Test findings only or minimal symptoms	Consider topical treatment or analgesia as per local guideline.	Continue study treatment.
	**2**<br>Moderate pain, reduced oral intake, limited instrumental activities	As per local guidelines, treat with analgesics, topical treatments and oral hygiene care. Ensure adequate hydration. If symptoms worsen or there is sepsis or bleeding, manage as a Grade 3 event.	Continue study treatment.
	**3**<br>Severe pain, limited food and fluid intake, daily living activity limited	Admit to hospital for appropriate management. Initiate IV (methyl)prednisolone 1-2 mg/kg/day. Convert to oral prednisolone when symptoms improved to Grade 2 and taper over at least 4 weeks.	Hold study treatment until improved to Grade 0-1.
	**4**<br>Life-threatening complications or dehydration	Admit to hospital for emergency care. Consider IV corticosteroids if not contraindicated by infection.	Discontinue study treatment.
**Myositis/ Rhabdomyolysis**	**1**<br>Mild weakness with/without pain	Prescribe analgesics.<br>If CK is significantly elevated and patient has symptoms, consider oral steroids and treat as Grade 2	Continue study treatment.
	**2**<br>Moderate weakness with/without pain	If CK is 3X ULN or worse, initiate oral prednisolone 0.5-1 mg/kg and taper over at least 4 weeks	Hold study treatment until improved to Grade 0-1

Autoimmune Toxicity	Grade	Treatment Guidelines (Subject to Clinical Judgement)	Study Drug Management
	**3-4**<br>Severe weakness, limiting self-care	Admit to hospital and initiate oral prednisolone 1 mg/kg. Consider bolus IV (methyl)prednisolone and 1-2 mg/kg/day maintenance for severe activity restriction or dysphagia. If symptoms do not improve add immunosuppressant therapy. Taper oral steroids over at least 4 weeks	Hold study treatment until improved to Grade 0-1. Discontinue if any evidence of myocardial involvement
**Myocarditis**	**< 2**<br>Asymptomatic but significantly increased CK-MB or increased troponin OR clinically significant intraventricular conduction delay	Initiate cardiac evaluation under close monitoring with repeat serum testing; consider referral to a cardiologist.<br>If diagnosis of myocarditis is confirmed, treat as Grade 2	Hold study treatment.<br>If a diagnosis of myocarditis is confirmed, permanently discontinue study treatment in patients with moderate or severe symptoms. Patients with no symptoms or mild symptoms may not restart tislelizumab unless cardiac parameters have returned to baseline and after discussion with the study medical monitor.
	**2**<br>Symptoms on mild-moderate exertion	Admit to hospital and initiate oral prednisolone or IV (methyl)prednisolone at 1-2 mg/kg/day. Consult with a cardiologist and manage symptoms of cardiac failure according to local guidelines.<br>If no immediate response change to pulsed doses of (methyl)prednisolone 1g/day and add MMF, infliximab or anti-thymocyte globulin	
	**3**<br>Severe symptoms with mild exertion		
	**4**<br>Life-threatening		

8.6.2. Dose and schedule modifications zanubrutinib

Zanubrutinib (BGB-3111) should be held in case of:

- any unmanageable CTC °III AE and any CTC °IV AE (if they are potentially study-drug related and not due to underlying CLL and/or RT)

Weekly monitoring should be performed until the event resolves or improves to CTC °I or to baseline. Treatment with zanubrutinib can be postponed for a maximum of four weeks, however, if the event doesn´t resolve within three weeks, the investigator can decide to continue treatment with zanubrutinib after consultation of the GCLLSG study office.

8.6.2.1. Dose interruption and modification

The guidelines below should be followed for dose interruption or modification of zanubrutinib for hematologic and non-hematologic (other than hypertension adequately controlled with oral medication or asymptomatic laboratory events; laboratory events indicating liver or renal dysfunction will not be considered asymptomatic laboratory events) toxicities.

Toxicity Occurrence	Dose Level	Zanubrutinib Dose
First	0 = starting dose	Restart at 160 mg twice daily
Second	-1 dose level	Restart at 80 mg twice daily
Third	-2 dose level	Restart at 80 mg once daily
Fourth	Discontinue zanubrutinib	Discontinue zanubrutinib

Zanubrutinib may be restarted upon resolution of toxicity and per investigator discretion if held for a maximum of 28 consecutive days. If, in the investigator's opinion, it is in the patient's best interest to restart treatment after > 28 days, the GCLLSG study office should be consulted to discuss individual cases.

8.6.2.2. Zanubrutinib dose reduction for hematologic toxicity

Dosing will be held for individual patients under any of the following conditions, based on investigator assessment of study-drug relatedness:

- Grade 4 neutropenia (lasting > 10 days)
- Grade 4 thrombocytopenia (lasting > 10 days)
- Grade 3 thrombocytopenia associated with significant bleeding
- ≥ Grade 3 febrile neutropenia

For the first occurrence of hematologic toxicity, treatment may restart at full dose upon recovery of the toxicity to ≤ Grade 1 or baseline.

If the same event recurs, patients will restart at 1 dose level lower upon recovery of the toxicity to ≤ Grade 1 or baseline. A maximum of 2 dose reductions will be allowed. Patients with ≥ Grade 3 thrombocytopenia associated with significant bleed requiring medical intervention should be discussed with the medical monitor.

Asymptomatic treatment-related lymphocytosis should not be considered an AE. Patients with asymptomatic treatment-related lymphocytosis should remain on study treatment and continue with all study-related procedures.

8.6.2.3. Zanubrutinib dose reduction for non-haematologic toxicity

For non-haematological toxicities ≥ Grade 3, other than hypertension adequately controlled with oral medication or asymptomatic laboratory events (laboratory events indicating liver or renal dysfunction will not be considered asymptomatic laboratory events) suspected to be related to study drug treatment, study drug will be held until recovery to ≤ Grade 1 or baseline, then restart at original dose level.

If the event recurs at ≥ Grade 3, study drug will be held until recovery to ≤ Grade 1 or baseline, then restart at 1 dose level lower (level -1). If the event recurs at ≥ Grade 3 at level -1, drug will be held until recovery to ≤ Grade 1 or baseline, then restart at level -2. If the event recurs at ≥ Grade 3 at level -2, the patient will be discontinued from study treatment.

For patients with symptomatic and/or incompletely controlled ≥ Grade 3 atrial fibrillation, study drug may be restarted at either the original dose or at dose level -1; atrial fibrillation is controlled at the discretion of the treating investigator. Zanubrutinib should be permanently discontinued for any intracranial haemorrhage.

8.7. Reasons for permanent discontinuation of all study treatment

In all following cases all trial medication must immediately stopped:

- pregnancy in a female patient
- failure to use two methods of reliable contraception (as defined in paragraph 8.8.1.3)
- uncontrollable immune related disorders
- progressive multifocal leukoencephalopathy (PML)
- adverse event or intercurrent illness, that precludes further study treatment according to the investigator´s discretion
  Please note: in case only one of the two study drugs have to be discontinued, study treatment with the other(s) may be continued at the investigator´s discretion
- disease progression
- withdrawal of consent
- refusal to continue study treatment
- protocol violation, that - in the judgment of the investigator - rules out continuation of study drug
- death

The reason and date for discontinuation should be recorded in the appropriate form of the CRF and in the source documents. It should be stressed that a discontinuation of treatment (end of treatment: EOT) does not equal withdrawal from follow up or documentation.

8.8. Potential risks

In this chapter, the potential risks for all study drugs are described; for further detailed information please refer to the respective IB or SMPC.

8.8.1. Potential risks relevant with all study drugs

8.8.1.1. Cytopenias and infections

All cytotoxic drugs, as well as antibodies and also the targeted agents cause cytopenias with decreased levels of hemoglobin, neutrophil and platelet count. However, it needs to be considered that patients with CLL and RT commonly have pre-existing myelosuppression due to bone marrow infiltration or previous therapy. These abnormal laboratory values are clinically relevant if they drop below a certain level, as a neutropenia increases the patient´s risk for infections and a thrombocytopenia can lead to severe bleeding.

Cytopenias and infections mostly occur during treatment and the risk seems to be highest in the beginning of treatment because of pre-existing myelosuppression due to bone marrow infiltration or previous therapy. However, this risk also persists after termination of treatment, e.g. the B-cells may not recover after an anti-CD20 antibody treatment for months to years and this increases the risk of opportunistic and other infections, including also PML (progressive multifocal leukoencephalopathy) and hepatitis B reactivation (see below).

In certain cases red blood cell or platelet transfusions might be warranted; these and antibacterial, antiviral and antifungal prophylaxis should be administered at the treating physicians discretion. Primary prophylaxis with granulocyte colony stimulating factors (G-CSF) is recommended as per the ASCO,

EORTC, and ESMO guidelines, namely in patients with advanced age, relevant comorbidities and in case of previous prolonged neutropenias and infections, see also chapter 8.4.1 Permitted medications.

8.8.1.2. Immune related disorders

Treatment with checkpoint inhibitors can cause immune-related adverse events by unbalancing the immune system. This can clinically manifest with autoimmune-like phenomena affecting almost any organ, including skin, gastrointestinal, hepatic, pulmonary and endocrine systems [48]. These effects have been termed “immune-related adverse events” (irAEs). Based on a safety population of 439 patients with different malignant conditions (BGB-A317-001 study), various irAEs related to tislelizumab monotherapy have been described.

For instructions on the management of irAEs, please see 8.6.1 *Dose and schedule modifications*.

Hepatitis

Cases of acute hepatitis and abnormal liver function, including fatal hepatitis, have been reported with tislelizumab. Patients require liver function test monitoring, diagnostic evaluation, and treatment with corticosteroids and other immunosuppressants according to the management guidance provided in the study protocol. Unless significant metastatic liver disease is present, treatment with tislelizumab should be suspended at the onset of Grade 2 abnormal liver function. Treatment should be discontinued with Grade 4 abnormal liver function or if significantly abnormal values persist. Monitoring should continue until the abnormality has resolved. Patients who have recovered after Grade 3 liver function abnormalities may resume treatment only after agreement by the GCLLSG study office.

Pneumonitis

Cases of pneumonitis, including fatal pneumonitis, have been reported under treatment with tislelizumab

Patients should be monitored for dyspnea and respiratory tract signs, and evaluated with radiographic imaging. High-resolution computed tomography scanning and pulmonary function tests should be considered when the diagnosis is uncertain. Patients with persistent Grade 2 and all patients with Grade 3 or 4 worse pneumonitis should be treated with corticosteroids according to the management guidance in the study protocol. Treatment with tislelizumab should be suspended at the onset of suspected pneumonitis and discontinued with Grade 3 or 4 pneumonitis or persistent Grade 2 pneumonitis.

Myocarditis/Myositis

Fatal myocarditis and polymyositis was reported in 1 patient who received a single dose of tislelizumab, in combination with paclitaxel and cisplatin. The patient’s initial symptoms were dyspnea and tea-colored urine 2 weeks after starting treatment. Elevated urine and serum cardiac markers and skeletal muscle were reported. The patient died of multi-organ failure six days later.

In patients with creatine kinase elevation and muscle pain or weakness, suspend treatment and treat all patients with Grade 3 or worse symptoms with corticosteroids. Discontinue treatment in patients with persistent symptoms or any evidence of myocardial involvement. Patients who have recovered after non-life-threatening (Grade 3) reactions may resume treatment only after agreement by the GCLLSG study office.

Colitis

Cases of colitis and diarrhea have been reported with tislelizumab. Patients reporting abdominal pain,

diarrhea, and rectal hemorrhage should be evaluated, given a low residue diet, and treated with corticosteroids according to the management guidance in the study protocol. Treatment with tislelizumab should be suspended for Grade 2 colitis or moderate diarrhea, and permanently discontinued when the symptoms persist or become severe. Additional immunosuppressive treatment is required if severe symptoms worsen or persist for more than 72 hours.

Endocrinopathies

Tislelizumab is associated with diabetes mellitus, including hyperglycemia and ketoacidosis. Patients should be monitored for hyperglycemia and its symptoms. Treatment with tislelizumab should be suspended while hyperglycemia is corrected, including insulin treatment, where indicated. If insulin-dependent diabetes persists, discontinue treatment.

Cases of thyroiditis, including thyrotoxicosis and hypothyroidism have been reported. Thyroid replacement therapy should be instituted as required.

Hypophysitis has been reported in patients treated with tislelizumab. Hypophysitis should be suspected when symptoms of hypopituitarism are reported, such as persistent fatigue, weakness, and weight gain that are not explained by the underlying disease. Endocrinology evaluation, including adrenal stimulation testing, should be carried out when hypophysitis is suspected. Corticosteroid treatment and hormone replacement therapy should be instituted for significant endocrine insufficiencies.

Other Immune-related Reactions

The following immune-related events have been reported in patients with tislelizumab monotherapy: skin reactions (including rash and pruritus); arthralgia; hemolytic anemia, nephritis, proteinuria; encephalitis, neuropathy, arthritis, pancreatitis, stomatitis, uveitis, and dry eye. For clinically significant reactions, suspend treatment with tislelizumab and initiate corticosteroid treatment tapered over at least 1 month until recovered to Grade 1 or less. Permanently discontinue tislelizumab for persistent or Grade 4 reactions. Patients who have recovered after Grade 3 reactions may resume treatment only after agreement by the GCLLSG study office.

Rash

Frequently reported tislelizumab- related TEAEs included rash,rash maculo-papular, and dermatitis, rash erythematous, and rash papular .

Hemolytic transfusion reaction

Hemolytic transfusion reactions during administration of red cell transfusions have been reported in patients receiving tislelizumab and zanubrutinib for relapsed Waldenström macroglobulinemia (WM). Patients developed severe direct antiglobulin test (DAT) negative hemolysis and reticulocytopenia associated with transfusions. The events were assessed as related to tislelizumab and required high doses of steroids and in one case cyclophosphamide. Bone marrow biopsies showed profound profound reticulocytopenia and marked erythroid hypoplasia which suggests that erythroid precursors may have been a predominant target of the immune attack, which may account for the negative DAT. Further cases details were published elsewhere [49].

8.8.1.3. Teratogenicity and mutagenicity

Zanubrutinib:

The risk of fertility impairment was considered to be low as zanubrutinib had no impact on fertility or

early embryonic development to implantation in male or female rats at doses up to 300 mg/kg or histopathological changes in reproductive organs in rats or dogs in up to 91-day repeat-dose studies. No apparent treatment-related maternal or embryo-fetal toxicities were noted at doses up to 150 mg/kg/day in both rats and rabbits; no apparent teratogenicity was noted in the rabbit fetus at any dose levels; and the only teratogenicity included one 3-chambered heart (0.3%), one 2 chambered heart (0.3%), and five 3-chambered hearts (1.5%) noted in rat fetuses at doses of 30, 75, and 150 mg/kg/day, respectively.

Tislelizumab:

In 3-month toxicity studies in cynomolgus monkeys, no gross lesion or histopathological changes were noted in male or female reproductive organs. Based on the published data, blocking of the PD-1/PD-L1 pathway resulted in fetal loss in animal models. Therefore, in vivo reproductive toxicity studies were not conducted with tislelizumab. Patients should use effective methods of contraception throughout treatment and for at least 120 days after stopping treatment.

Given the limited data available, in this trial, a pregnancy must be prevented in both female patients of childbearing potential and partners of male patients in the trial during and 120 days after last dosage of study treatment.

Definition of women of childbearing potential and contraception

Within this study, all women of childbearing potential (WOCP) must have a negative pregnancy test ≤7 days prior initiation of study treatment. A woman of childbearing potential is defined as any female who does not meet the criteria of non-childbearing potential. These are as follows:

- documented hysterectomy, bilateral oophorectomy (ovarectomy), or bilateral tubal ligation
- post-menopausal (a practical definition accepts menopause ≥1 year without menses with an appropriate clinical profile, e.g. age >45 years in the absence of hormone replacement therapy (HRT). In questionable cases, the subject must have a follicle stimulating hormone (FSH) value >40 mIU/ml and an estradiol value < 40pg/ml.

Sexually active men and women of child-bearing potential must use two methods of reliable contraception including one highly effective (Pearl Index <1) and one additional effective (barrier) method as described below while on study and maintained for up to 120 days after the last dose of CLL-RT1 study therapy.

The following **contraceptive methods with a Pearl Index <1** are regarded as highly-effective:

- oral hormonal contraception (‘pill’)
  Please note: in case that its efficacy is impaired during the trial, e.g. due to vomiting and diarrhea, additional/other methods as listed below are required to assure adequate safety
- dermal hormonal contraception/contraceptive plaster
- vaginal hormonal contraception (NuvaRing®)
- long-acting injectable contraceptives/implants that release progesterone (Implanon®)
- tubal ligation (female sterilisation)
- intrauterine devices that release hormones (hormone spiral)
- double barrier methods
- partner’s vasectomy

Additional effective (barrier) methods are:

- male condom

- diaphragm/cervical cap

The following contraceptive methods are not regarded as safe: condom plus spermicide, simple barrier methods (vaginal pessaries, condom, female condoms), copper spirals, rhythm/basal temperature method and withdrawal method (coitus interruptus).

A **pregnancy must be prevented during study treatment and afterwards for the below listed time periods** after last administration of the respective study drug:

Female patients or partners of male patients:
- at least 120 days after last infusion of tislelizumab,
- at least 30 days after last administration of zanubrutinib.

Since blood levels and effectiveness of steroidal contraceptives may be reduced by zanubrutinib, the hormonal contraceptive method must be supplemented with a second barrier method of contraception (preferably male condom).

Women with childbearing potential must undergo a pregnancy test at screening, at each month, in case of delayed menstrual period (over one month between menstruations) and one month after last study drug intake. Suitable pregnancy tests are urine or serum pregnancy tests with a sensitivity of at least 25 mIU/ml.

If a female subject becomes pregnant during treatment or within the above listed time periods, all study treatment must be discontinued, and the sponsor must be notified immediately.

Male patients are requested to refrain from sperm donation from the initial study drug administration until at least six months after last administration of study drug.

Nursing women are excluded in this trial as it is not known whether the study drugs or metabolites are excreted in human milk and the potential for absorption and harm to the infant is unknown.

1.1.1. Cardiac adverse events

Patients with CLL patients are often elderly, sometimes have cardiac comorbidities and have received multiple prior chemotherapeutic agents. Therefore, an ECG should be performed at screening and every six months. Cardiac ultrasound for assessment of LVEF is recommended at screening (per investigator's discretion) and as clinically indicated afterwards. Worsening of preexisting cardiac disease and adverse events such as angina pectoris, acute coronary syndrome, myocardial infarction, heart failure and arrhythmias, including atrial fibrillation and tachyarrhythmia have been observed during treatment with most CLL therapies. Therefore, patients with a history of cardiac disease should be monitored closely and should be hydrated with caution in order to prevent a potential fluid overload.

Zanubrutinib

Assays showed that zanubrutinib was more selective than ibrutinib for the inhibition of kinase activity of BTK vs. EGFR, FGR, ITK, FRK, JAK3 and others. Thus, it is suggested that the rate of cardiac arrythmias, which is approx. 6% with ibrutinib [50], might be reduced with zanubrutinib. According to the current version of the IB for zanubrutinib (version 5 as of February 2018), one case of atrial fibrillation has been reported. Apart from that, two other related cardiac Aes have been reported, one congestive heart failure and one myocardial ischemia.

8.8.2. Further potential risks with tislelizumab

Infusion-related reactions (IRRs) and anaphylaxis:

Infusion-related reactions have been reported in 4.1% of patients treated with tislelizumab monotherapy. There have been infrequent reports of severe infusion-related reactions, characterized by systemic symptoms that start during or after the infusion. These include flushing, dyspnea, hypotension, chills, pruritus, and rash. For severe reactions, stop the infusion. For mild or moderate symptoms, slow the rate of infusion by 50%. Further details are provided in section 8.2.3.1 *Management of infusion-related reactions (IRRs)*.

8.8.3. Further potential risks with zanubrutinib

Bleeding events:

Serious and fatal haemorrhagic events have occurred in patients treated with Zanubrutinib monotherapy. Grade 3 or higher bleeding events including intracranial and gastrointestinal haemorrhage, haematuria and haemothorax have been reported in patients (see section 4.8). Bleeding events of any grade including purpura and petechiae occurred in patients with haematological malignancies. The mechanism for the bleeding events is not well understood. Zanubrutinib may increase the risk of haemorrhage in patients receiving antiplatelet or anticoagulant therapies and patients should be monitored for signs of bleeding. Dose modification may be necessary for Grade 3 or greater adverse reactions as recommended (see section 8.6.2.). Warfarin or other vitamin K antagonists should not be administered concomitantly with Zanubrutinib. The co-administration of heparine, direct Xa or thrombin inhibitors, or antiplatelet agents together with zanubrutinib is permitted after careful risk-benefit assessment (see chapter *8.4.1.5 Anticoagulants*). Patients should be observed closely for signs and symptoms of bleeding and the coagulation should be monitored carefully. In case of severe bleeding events, zanubrutinib should be withheld. Also, it should be considered to withhold zanubrutinib at least three days before and after surgery (see chapter *8.4.2 Surgery*).

Infections

Fatal and non-fatal infections (including bacterial, viral, fungal infections, or sepsis) and opportunistic infections (e.g. herpes viral, cryptococcal, aspergillus and pneumocystis jiroveci infections) have occurred in patients treated with Zanubrutinib monotherapy. Grade 3 or higher infections occurred in patients. The most common Grade 3 or higher infection was pneumonia. Infections due to hepatitis B virus (HBV) reactivation have also occurred. Before initiating treatment with Zanubrutinib, patients' HBV status should be established. Consultation with a liver disease expert physician is recommended for patients who test positive for HBV or have positive hepatitis B serology, before initiating treatment. Patients should be monitored and managed according to the medical standards to prevent hepatitis B reactivation. Consider prophylaxis according to standard of care in patients who are at increased risk for infections. Patients should be monitored for signs and symptoms of infection and treat appropriately

Second primary malignancies

Second primary malignancies, including non-skin carcinoma have occurred in patients treated with Zanubrutinib monotherapy. The most frequent second primary malignancy was skin cancer (basal cell carcinoma and squamous cell carcinoma of skin). Advise patients to use sun protection.

Atrial fibrillation and flutter

Atrial fibrillation and atrial flutter have occurred in patients treated with Zanubrutinib monotherapy, particularly in patients with cardiac risk factors, hypertension, and acute infections. Monitor signs and symptoms for atrial fibrillation and atrial flutter and manage as appropriate.

Tumor lysis syndrome:

Singular cases of tumor lysis syndrome in patients with CLL and high tumor burden treated with zanubrutinib have been observed. Particularly patients with high tumor burden at start of treatment should

be monitored carefully for signs of TLS, and appropriate measures, such as hydration, diuretics or rasburicase, should be taken.

8.9. Special Situation Report Form

Whenever a serious protocol violation regarding intake of study medication occurs (e.g. overdose, intake of wrong medication, damaged medication resulting in an AE, abuse) the event has to be reported on the special situation report form to GCLLSG office via fax or email.

9. Measurement of efficacy and safety variables

Serial measurements of efficacy, safety and exploratory variables will be performed at screening and at scheduled intervals throughout the duration of the study as outlined in IV. *Study assessment table*. All scheduled staging visits will have a ± 7-day window unless otherwise stated.

9.1. Efficacy variables

- Lymph nodes, spleen and liver measurements by physical examination
- Ultrasound of abdomen and for measurement of enlarged lymph nodes, spleen and liver
- Computed tomography (CT) or magnetic resonance imaging (MRI) scans for confirmation of a CR at first or final restaging and/or before treatment termination (for confirmation of a CR) and whenever clinically indicated
- PET scan at final restaging
- Complete blood count (CBC)
- Bone marrow aspirate/biopsy for histopathology and/or before treatment termination (for confirmation of a CR) and whenever clinically indicated (e.g. unclear cytopenia)
- Assessment of constitutional symptoms
- Survival status
- Survey of start and type of next treatment for CLL
- Lymph node / bone marrow biopsy at baseline (mandatory) and after relapse/disease progression for genome sequencing studies in patients who voluntarily agree to this procedure

9.2. Safety variables

- Clinical laboratory evaluations
- ECOG Performance Status
- Assessment of comorbidity burden by CIRS-Score and concomitant medications
- Aes by NCI CTCAE Version 5
- HBV-DNA PCR every two months in patients with positive anti-HBc (irrespective of HbsAg) at screening
- pregnancy test ≤ 7 days before start of treatment for all women of childbearing potential

9.3. Laboratory analyses

All hematology, serum chemistries and coagulation analyses (including platelet function for a subgroup of patients), the screening for HIV, HBV and HCV and pregnancy tests (if applicable) will be performed by the local laboratory of each investigational site. Also, bone marrow aspirate/biopsy and lymph node or other tissue will be examined by the local pathologist and/or hematological laboratory. However, the written pathology report as well as the paraffin-embedded specimen (in exceptional cases unstained slides) have to be shipped to Cologne.

Central laboratory assessment will be performed for initial immunophenotyping (including CD38 and ZAP70 expression), the serum parameters beta-2-microglobulin and thymidine kinase, cytogenetics by FISH, molecular genetics (e.g. IGHV and TP53 mutation, as well as BTK and PLCg2 mutations in patients with prior ibrutinib treatment) and karyotyping, as well as scientific analyses. (see 5 *Central laboratory assessment)*

9.4. Response assessment

Response will be assessed on the basis of the guidelines of the international workshop on CLL (iwCLL) [13] as well as the Lugano classification [51], see below.

The first response assessment will be performed after 6 cycles of therapy, i.e. at cycle 7 day 1 and the final response will be assessed after 12 cycles of therapy. In case of a progression or start of new treatment for CLL or RT (including allogenic SCT), the patient has to be staged immediately prior to administration of any new treatment. Follow-up visits will be performed every month for 6 months to capture survival status and subsequent treatments.

9.4.1. Definition of measurable sites of disease

Measurable sites of disease are defined as lymph nodes/lymph node masses, enlarged liver or spleen or any extranodal manifestation of CLL/RT. The presence of enlarged lymph nodes, hepato- and splenomegaly and their size is determined by clinical palpation. A CT scan/MRI of head/neck, chest, abdomen and pelvis/inguinal region is required at baseline and should be repeated at the interim staging if criteria for CR are fulfilled and for all patients after 12 cycles of therapy. It is recommended to perform ultrasound examinations (at least of the abdomen) at the staging visits in between.

According to the iwCLL guidelines, lymph nodes are considered enlarged if ≥1.0 cm in diameter at baseline, however, during and after treatment the cutoff for pathological lymph nodes is ≥1.5cm in diameter. Likewise, the response assessment of NHL according to the Lugano Guidelines includes a cutoff for pathological lymph nodes of >1.5 cm.

It is difficult to determine a cutoff for the size of liver and spleen as their size also varies with the size of the patient and potentially also due to his/her comorbidities. Spleen and liver are usually not palpable below the respective costal margin and if enlarged in the clinical examination the size below the costal margin should be indicated in cm. For the measurement in a radiological examination, a usual cutoff for the spleen is 12 cm (largest diameter) and for the liver 17 cm (measured in the medio-clavicular line), however, in this trial a spleen size of 12 to 14 cm and any liver size may be considered normal if the treating physician confirms that this is due to the patient's constitution or a comorbidity documented in the CIRS score.

9.4.2. Response criteria

Response assessment will be based on the revised iwCLL guidelines of 2018 [13] and the Lugano Classification of 2014 [51].

Table 1 **Response criteria according to iwCLL guidelines**

Group	Parameter	CR	PR	PD	SD
A	Lymph nodes	None ≥1.5 cm	Decrease ≥50% (from baseline)*	Increase ≥50% from baseline or from response	Change of −49% to +49%
	Liver and/or spleen size±	Spleen size <13 cm; liver size normal	Decrease ≥50% (from baseline)	Increase ≥50% from baseline	Change of −49% to +49%

Group	Parameter	CR	PR	PD	SD
				or from response	
	Constitutional symptoms	None	Any	Any	Any
	Circulating lymphocyte count	Normal	Decrease ≥50% from baseline	Increase ≥50% over baseline	Change of −49% to +49%
	Platelet count	≥100 × 10^9/L	≥100 × 10^9/L or increase ≥50% over baseline	Decrease of ≥50% from baseline secondary to CLL	Change of −49 to +49%
	Hemoglobin	≥11.0 g/dL (untransfused and without erythropoietin)	≥11 g/dL or increase ≥50% over baseline	Decrease of ≥2 g/dL from baseline secondary to CLL	Increase <11.0 g/dL or <50% over baseline, or decrease <2 g/dL
B	Marrow	Normocellular, no CLL cells, no B-lymphoid nodules	Presence of CLL cells, or of B-lymphoid nodules, or not done	Increase of CLL cells by ≥50% on successive biopsies	No change in marrow infiltrate

* Sum of the products of 6 or fewer lymph nodes (as evaluated by CT scans and physical examination in clinical trials or by physical examination in general practice).

† Spleen size is considered normal if 13 cm. There is not firmly established international consensus of the size of a normal liver; therefore, liver size should be evaluated by imaging and manual palpation in clinical trials and be recorded according to the definition used in a study protocol.

CR complete remission (all of the criteria have to be met);

PD progressive disease (at least 1 of the criteria of group A or group B has to be met);

PR partial remission (for a PR, at least 2 of the parameters of group A and 1 parameter of group B need to improve if pre viously abnormal; if only 1 parameter of both groups A and B is abnormal before therapy, only 1 needs to improve);

SD stable disease (all of the criteria have to be met; constitutional symptoms alone do not define PD).

Table 2 **Response criteria according to Lugano Classification**

Response and site	PET-CT based response	CT-based response
**Complete**	**Complete metabolic response**	**Complete radiologic response (all of the following)**
Lymph nodes and extralymphatic sites	Score 1, 2, or 3[a] with or without a residual mass on 5PS[b]<br>It is recognized that in Waldeyer's ring or extranodal sites with high physiologic uptake or with activation within	Target nodes/nodal masses must regress to ≤ 1.5 cm in LDi<br>No extralymphatic sites of disease

	spleen or marrow (eg, with chemotherapy or myeloid colonystimulating factors), uptake may be greater than normal mediastinum and/or liver. In this circumstance, complete metabolic response may be inferred if uptake at sites of initial involvement is no greater than surrounding normal tissue even if the tissue has high physiologic uptake.	
Non-measured lesion	Not applicable	Absent
Organ enlargement	Not applicable	Regress to normal
New lesions	None	None
Bone marrow	No evidence of FDG-avid disease in marrow	Normal by morphology; if indeterminate, IHC negative
**Partial**	**Partial metabolic response**	**Partial remission (all of the following)**
Lymph nodes and extralymphatic sites	Score 4 or 5[b] with reduced uptake compared with baseline and residual mass(es) of any size<br>At interim, these findings suggest responding disease<br>At end of treatment, these indicate residual disease	≥ 50% decrease in sum of perpendicular diameters (SPD) of up to 6 target measurable nodes and extranodal sites<br>When a lesion is too small to measure on CT, assign 5 mm × 5 mm as the default value<br>When no longer visible, 0 × 0 mm<br>For a node > 5 mm × 5 mm, but smaller than normal, use actual measurement for calculation
Non-measured lesion	Not applicable	Absent/normal, regressed, but no increase
Organ enlargement	Not applicable	Spleen must have > 50% in length beyond normal
New lesions	None	None
Bone marrow	Residual uptake higher than uptake in normal marrow but reduced compared with baseline (diffuse uptake compatible with reactive changes from chemotherapy allowed). If there are persistent focal changes in the marrow in the context of a nodal response, consideration should be	Not applicable

	given to further evaluation with MRI or biopsy or an interval scan	
**No response or stable disease**	**No metabolic response**	**Stable disease**
Target nodes/nodal masses, extranodal lesions	Score 4 or 5b with no significant change in FDG uptake from baseline at interim or end of treatment	<50% decrease from baseline in SPD of up to 6 dominant measurable nodes and extranodal sites; no criteria for progressive disease are met
Non-measured lesion	Not applicable	No increase consistent with progression
Organ enlargement	Not applicable	No increase consistent with progression
New lesions	None	None
Bone marrow	No change from baseline	Not applicable
**Progressive disease**	**Progressive metabolic response**	**Progressive disease requires at least 1 of the following**
Individual target nodes/nodal masses<br><br>Extranodal lesions	Score 4 or 5[b] with an increase in intensity of uptake from baseline and/or<br>New FDG-avid foci consistent with lymphoma at interim or end-of-treatment assessment	PPD progression:<br><br>An individual node/lesion must be abnormal with:<br>LDi > 1.5 cm and<br>Increase by ≥ 50% from PPD nadir and<br>An increase in LDi or SDi from nadir<br>0.5 cm for lesions ≤ 2 cm<br>1.0 cm for lesions > 2 cm<br><br>In the setting of splenomegaly, the splenic length must increase by > 50% of the extent of its prior increase beyond baseline (e.g., a 15 cm spleen must increase to > 16 cm). If no prior splenomegaly, must increase by at least 2 cm from baseline<br>New or recurrent splenomegaly
Non-measured lesions	None	New or clear progression of pre-existing non-measured lesions
New lesions	New FDG-avid foci consistent with lymphoma rather than another etiology (eg, infection, inflammation). If	Regrowth of previously resolved lesions<br>A new node > 1.5 cm in any axis

	uncertain regarding etiology of new lesions, biopsy or interval scan may be considered	A new extranodal site > 1.0 cm in any axis, if < 1.0 cm in any axis, its presence must be unequivocal and must be attributable to lymphoma<br><br>Assessable disease of any size unequivocally attributable to lymphoma
Bone marrow	New or recurrent FDG-avid foci	New or recurrent involvement

Abbreviations: 5PS = 5-point scale; CT = computed tomography; FDG = fluorodeoxyglucose; GI = gastrointestinal; IHC = immunohistochemistry; LDi = longest transverse diameter of a lesion; MRI = magnetic resonance imaging; PET = positron emission tomography; PPD = cross product of the LDi and perpendicular diameter; SDi = shortest axis perpendicular to the LDi; SPD = sum of the product of the perpendicular diameters for multiple lesions.

[a] A score of 3 in many patients indicates a good prognosis with standard treatment, especially if at the time of an interim scan. However, in trials involving PET where de-escalation is investigated, it may be preferable to consider a score of 3 as inadequate response (to avoid undertreatment). Measured dominant lesions: Up to six of the largest dominant nodes, nodal masses, and extranodal lesions selected to be clearly measurable in two diameters. Nodes should preferably be from disparate regions of the body and should include, where applicable, mediastinal and retroperitoneal areas. Non-nodal lesions include those in solid organs (e.g., liver, spleen, kidneys, and lungs), GI involvement, cutaneous lesions, or those noted on palpation. Non-measured lesions: Any disease not selected as measured, dominant disease and truly assessable disease should be considered not measured. These sites include any nodes, nodal masses, and extranodal sites not selected as dominant or measurable or that do not meet the requirements for measurability but are still considered abnormal, as well as truly assessable disease, which is any site of suspected disease that would be difficult to follow quantitatively with measurement, including pleural effusions, ascites, bone lesions, leptomeningeal disease, abdominal masses, and other lesions that cannot be confirmed and followed by imaging. In Waldeyer's ring or in extranodal sites (e.g., GI tract, liver, bone marrow), FDG uptake may be greater than in the mediastinum with complete metabolic response, but should be no higher than surrounding normal physiologic uptake (e.g., with marrow activation as a result of chemotherapy or myeloid growth factors).

[b] PET 5PS: 1, no uptake above background; 2, uptake ≤ mediastinum; 3, uptake > mediastinum but ≤ liver; 4, uptake moderately > liver; 5, uptake markedly higher than liver and/or new lesions; X, new areas of uptake unlikely to be related to lymphoma.

10. Safety

For all safety issues or questions regarding the study please contact the safety management of the GCLLSG study office:

Email: **cll-safety@uk-koeln.de**
Fax-number: **+49-221-478-86886**
Phone-number: **+49-221-478-88220**

10.1. Reporting periods

The reporting period of all serious adverse events (SAEs), adverse events (AEs), AEs of special interest (AESIs) and AEs of particular interest (AEPIs) starts with the administration of the first dose of trial medication. All SAEs have to be reported until the end of the study, In case the patient withdraws his/her consent for documentation, no further AEs/SAEs and AESIs/AEPIs (occurring after the withdrawal of consent) may be documented.

Table 3 **Overview of reporting periods and documentation**

**Event**	**Reporting period**		**Documentation**
	**Start**	**End**	
**SAEs**<br>(related and unrelated)	after first dose of study medi-cation	end of the study	AE Form and additional SAE Module required
**AEs**<br>(related or unrelated)	after first dose of study medi-cation	28 days after end of treatment	only AE Form required
**AEs of Special interest**[1]<br>- major hemorrhage (see below)<br>- cardiovascular events<br>- immune related adverse events (any grade)<br>- infections (CTC°III or higher)	after first dose of study medi-cation	end of study or start of new treatment	AE Form and additional SAE Module Required
**AEs of particular interest**<br>- second primary malignancies<br>- infections (CTC°II or higher)	after first dose of study medi-cation	end of the study	only AE Form required
**Pregnancy**	after first dose of study medi-cation	end of the study	Pregnancy form required

[1] Not fulfilling SAE criteria

10.2. Definitions of AE, ADR, SAE, SADR, SUSAR

10.2.1. Adverse event (AE) and adverse drug reaction (ADR)

An **adverse event (AE)** is any untoward medical occurrence after starting study drug treatment, regardless of whether the event is considered related or unrelated to the study drug.

An **adverse drug reaction (ADR)** is any noxious and unintended response to any of the components of the study medication related to any dose with at least a reasonably possible causal relationship with the study medication.

An AE can therefore be any unfavorable and unintended sign (including an abnormal laboratory finding), symptom, or disease temporally associated with the use of a medicinal (investigational) product, whether or not considered related to the medicinal (investigational) product.

The investigator will evaluate changes in physical signs, laboratory values, or other diagnostic procedures in determining AEs. Subjective AEs should be elicited by first questioning the subject in a non-directive manner, then, if any unfavorable symptoms are reported, questioning in a more detailed manner to obtain the information necessary for reporting the event. AEs should be documented in the source documents and on the appropriate pages of the CRF. The NCI Common Terminology Criteria for Adverse Events (NCI-CTCAE), Version 5, will be used for assessing the severity of AEs. MedDRA will be used for the coding of adverse events.

Please note: Disease progression should not be recorded as an adverse event or serious adverse event term. In addition any hospitalizations planned for further therapies i.e. stem cell transplantation or CAR-T-cell- therapy and hospitalizations for expected adverse events of these subsequent therapies i.e. graft-versus-host disease should not be recorded as an adverse event or serious adverse event.

10.2.2. Adverse events of special interest (AESIs)

Certain adverse events are considered to be AEs of special interest (AESIs) due to their observed frequency and/or clinical relevance, please see *10.3.4 AEs of special interest*.

10.2.3. Adverse events of particular interest (AEPIs)

Patients with CLL have abnormal cellular and humoral immune responses due to quantitative and qualitative defects in immune effector cells. Therefore, secondary malignancies, which may be related to the immunodeficiency due to CLL, are considered to be AEs of particular interest (AEPIs) due to their clinical relevance please see *10.3.5 AEs of particular interest*.

10.2.4. Serious adverse events (SAEs) and serious adverse reactions (SADRs)

Serious adverse events (SAEs) or serious adverse drug reactions (SADR) are untoward medical occurrence that at any dose, that

- result in death
- are life-threatening at the time of the event
- require inpatient hospitalization or prolongation of existing hospitalization
- result in persistent or significant disability/incapacity
- are a congenital anomaly or birth defect (§ 3(8) GCP Regulations)
- are medically significant.

Medical and scientific judgment should be exercised in deciding whether expedited reporting is also appropriate in other situations, such as important medical events that may not be immediately life threatening or result in death or hospitalization but may jeopardize the subject or may require intervention to prevent one of the other outcomes listed in the definition above. These should usually be considered serious.

Please note: In-patient hospitalization is defined as any stay in hospital that includes at least one night (midnight to 06:00). Admissions to hospital for the following reasons are **not SAEs** but must be documented in a proper manner in the patient's medical records and CRF:

- before the first administration of study drug,
- planned before the first administration of the study drug,
- planned for the administration of the study drug or management of the tumor-lysis prophylaxis,
- hospitalizations and life-threatening situations or death due to the progression of CLL/ Richter transformation.

10.2.5. Suspected unexpected serious adverse reactions (SUSAR)

A suspected unexpected serious adverse reaction (SUSAR) is an adverse event the nature or severity of which is not consistent with the reference safety information as listed in section*10.5.2 Reference safety information documents* is regarded as serious and has at least a possible causal relationship with the study drug.

10.3. Exceptions and specialties

10.3.1. Lab abnormalities

Lab abnormalities are only considered as an AE if they fulfill at least one of the following criteria:

- accompanied by clinical symptoms
- leading to a change in study medication (e.g. dose modification, interruption or permanent discontinuation)
- leading to a premature control of the abnormal value before the next scheduled blood test
- requires a change in concomitant therapy (e.g. addition or change in a concomitant medication, therapy or treatment)

E.g. lymphopenia is an expected and desired effect of therapy and should not be reported as an adverse event or serious adverse event.

10.3.2. Concomitant diseases

Preexisting diseases (present before first administration of the study drug) are not documented as adverse events but as concomitant diseases. New diseases and preexisting diseases that worsen during the trial are documented as (S)AEs.

A diagnosis or syndrome, rather than the individual signs or symptoms of the diagnosis or syndrome should be recorded on the AE page of the CRF.

10.3.3. Pregnancy

Any subject who becomes pregnant during the study must be promptly discontinued from further study treatment.

All initial reports of pregnancy must be reported to the sponsor by the study-site personnel within 24 hours of their knowledge of the event using the appropriate pregnancy report form. Abnormal pregnancy outcomes (e.g. spontaneous abortion, stillbirth, and congenital anomaly) are considered serious ad-

verse events and must be reported using the Serious Adverse Event Form. Follow-up information regarding the outcome of the pregnancy and any postnatal sequelae in the infant will be required. Because the effect of the study drug on sperm is unknown, pregnancies in partners of male patients included in the study will, provided free consent is given, also be reported by the study-site personnel within 24 hours of their knowledge of the event using the appropriate pregnancy report form.

10.3.4. AEs of special interest

The following AEs of special interest (AESI) will be reported as AEs and SAE (considered an "Important Medical Event" even if no other criteria for seriousness apply) during the entire duration of the study or until start of a new anti-cancer treatment whichever occurs first.

Major hemorrhage:

- any bleeding CTC grade ≥3
- intraocular bleeding causing loss of vision
- bleeding that requires a transfusion of ≥2 units of packed red cells or an equivalent amount of whole blood
- bleeding that results in a hospitalization, or prolongation of hospitalization
- subdural hematoma/hemorrhage of any grade of severity
- epidural hematoma/hemorrhage of any grade of severity
- intracerebral hemorrhage of any grade of severity

Cardiovascular events

Infections (CTC°III or higher)

Infectious complications have a significant impact on the clinical course of patient. Due to the changes in the immune system and immune deficiency of CLL patients, it may be difficult to distinguish between the occurrence of infections related to the disease itself or due to the therapy. As new agents and treatment approaches are tested in clinical settings, it is important to evaluate not only the impact of these regimen on the disease itself, but to assess the influence of these agents on the immune function and resulting infectious complications as well. It is important to identify patient subsets that are at increased risk for infections.

The severity of infection should be quantified according to the Common Toxicity Criteria (CTC) Version 5. Infections of CTC °III or higher should be reported as SAEs during the entire duration of the study.

Immune related adverse events (irAEs)

Treatment with checkpoint inhibitors can cause immune-related adverse events by unbalancing the immune system. This can clinically manifest with autoimmune-like phenomena affecting almost any organ, including skin, gastrointestinal, hepatic, pulmonary and endocrine systems. Moreover, some autoimmune complications like autoimmune hemolytic anemia (AIHA), idiopathic thrombocytopenic purpura (ITP) and pure red cell aplasia (PRCA) can occur in treatment-naïve as well as treated CLL patients. The severity should be quantified according to the Common Toxicity Criteria (CTC) Version 5. All grades of irAEs have to be documented and should be reported as AEs and SAEs during the entire duration of the study.

10.3.5. AEs of particular interest

The following AEs of particular interest (AEPI) will be reported as AEs and SAE (if any criterion for

seriousness applies) during the entire duration of the study (also after start of a new anti-cancer treatment).

Second primary malignancies

Patients with CLL have an increased risk for the development of other malignant neoplasms and multiple factors, such as a profound immunosuppression with abnormal cellular and humoral immune responses due to quantitative and qualitative defects in immune effector cells, but also the use of chemotherapeutic agents may contribute to this phenomenon [52]. All grades of secondary malignancies have to be documented as AEs and SAEs (only if at least one criterion for seriousness is fulfilled) during the entire duration of the study.

Infections °II: Non-serious infections of CTC °II should be reported as AEs during the entire duration of the study.

10.3.6. Documentation and follow up of AE and SAE

The sponsor ensures that all persons involved in the treatment of patients in the trial are adequately informed of the responsibilities and actions required when AEs occur. Patients will be asked at each visit whether they have experienced AEs or SAEs. AEs will be documented in the patient's medical records and in the CRF according to the reporting periods and documentation requirements listed in 10.1 *Reporting periods*:

- term (will be coded with MedDRA)
- date of onset and resolution
- severity (quantified according to the Common Toxicity Criteria (CTC) Version 5)
- seriousness
- causal relationship with study treatment
- interruption or withdrawal of study treatment and other measures taken.

Regardless of whether a causal relationship between the SAE/AE and one of the study drugs is suspected, patients who develop (serious) adverse events must be monitored until:

- AE/SAE resolved or improved to baseline
- investigator confirms that no further improvement can be expected
- patient´s death
- clinical or safety data will no longer be collected as the patient is considered end of study
- the event can be attributed to agents other than the study drug or to factors unrelated to study conduct
- it becomes unlikely that any additional information can be obtained (subject or health care practitioner refuses to provide additional information, lost to follow-up after demonstration of due diligence with follow-up efforts).

10.3.7. Severity of adverse events

The Common Toxicity Criteria (CTC) Version 5 will be used to describe and classify the AEs and the severity of the AEs will be classified as follows:

- CTC °I: mild; asymptomatic or mild symptoms; clinical or diagnostic observations only; intervention not indicated.
- CTC °II: moderate; minimal, local or non-invasive intervention indicated; limiting age-appropriate instrumental Activities of Daily Living (ADL).
- CTC °III: severe or medically significant but not immediately life-threatening; hospitalization or

prolongation of hospitalization indicated; disabling; limiting self-care Activities of Daily Living (ADL).

- CTC °IV: life-threatening consequences; urgent intervention indicated.
- CTC °V: death related to AE.

10.3.8. Causal relationship between AE and study drug

The investigator will assess every AE whether a causal relationship with the study drugs can be assumed. The assessment includes consideration of the nature and type of reaction, the temporal relationship with the administration of the drug, the clinical status of the patient, concomitant medication and other relevant clinical factors. If the event is considered being related to the underlying disease due to lack of efficacy of the study drug or is evaluates as a symptom or sign of the underlying disorder, no causal relationship will be assumed.

The Investigator(s) must determine the relationship between the administration of study drug and the occurrence of an AE/SAE as "not related" or "related" as defined below:

- Not related: The temporal relationship of the adverse event to study drug administration makes a causal relationship unlikely or remote, or other medications, therapeutic interventions, or underlying conditions provide a sufficient explanation for the observed event.
- Related: The temporal relationship of the adverse event to study drug administration makes a causal relationship possible, and other medications, therapeutic interventions, or underlying conditions do not provide a sufficient explanation for the observed event.

10.4. Reporting of SAE, pregnancy and changes in risk-benefit assessment

At the time of the initial report, the below listed information should be provided. In case of incomplete information, a SAE should be reported including the information printed in bold letters (minimal criteria) signed by the investigator. The missing data need to be supplemented in the follow up report.

- study identifier
- study Center and **subject number**
- **term of the event**
- **documentation of study medication**
- description of the event
- date of onset
- outcome
- whether study treatment was discontinued
- the reason why the event is classified as serious
- investigator assessment of the association between the event and the study treatment

10.4.1. Reports from the investigator to the sponsor

As outlined above, the investigator must complete the Serious Adverse Event (SAE) report form and send it via **eCRF** or by facsimile (only if the access to the clinical data base is not possible) immediately, without undue delay, under no circumstances later than 24 hours after becoming aware of the event to the sponsor`s designee:

GCLLSG study office fax-number: +49-221-478-86886

All SAEs **must** be collected and reported according section 10.1 *Reporting periods*. The definition and reporting requirements of the EU Good Clinical Practice Guideline CPMP/ICH/135/95 will be adhered to.

The original and the duplicate copies of the SAE report form, as well as the fax confirmation sheet must be kept with the case report forms at the study site. Follow-up information of a previously reported SAE must also be reported to the GCLLSG study office within 24 hours and should be sent to the sponsor or designee by fax, re-stating the date of the original report. Either a new SAE report form is sent (stating that this is a follow-up), or the original one resubmitted (with the new information highlighted and a new date provided). The follow-up report should describe whether the event has resolved or continues, if and how it was treated, and whether the subject continued or discontinued study participation. The form and fax confirmation sheet must also be retained.

The investigator will inform the sponsor without delay about any pregnancy that occurs during the trial, i.e. within 24 hours of becoming made aware of such. This will be documented on a separate pregnancy reporting form.

10.5. Assessment and reporting responsibilities of the sponsor

10.5.1. Assessment of event by sponsor

All cases of suspected SAEs are assessed by the sponsor with regard to seriousness, causality and expectedness, regardless of the investigator's assessments.

10.5.2. Reference safety information documents

In this trial the current versions of the below listed documents will be used for the assessment of the expectedness.

- Tislelizumab (BGB-A317): investigator's brochure (IB)
- Zanubrutinib (BGB-3111): investigator's brochure (IB)

10.5.3. Notification of ethics committee and competent supreme federal authority

Every SUSAR that becomes known in the trial will be reported by the sponsor to the competent authorities and the ethics committee. Reference documents for safety information are the above listed papers.

Fatal and life-threatening SUSARs

The responsible competent authorities and the leading ethics committee must be informed by the sponsor of all fatal or life-threatening SUSARs. This must be done without delay, at the latest 7 calendar days after becoming aware of the minimum criteria for reporting (see above). In all cases, attempts must be made to obtain further relevant information, which must be supplied to the competent supreme federal authority and the ethics committee within a further 8 days.

SUSARs that are not fatal or life threatening

The responsible competent authorities and the leading ethics committee will be informed without delay by the sponsor of all SUSARs, at the latest within 15 calendar days of becoming aware of the minimum criteria for reporting. Further relevant details will be passed on as soon as possible. If the information at the time of reporting is incomplete, further information to enable adequate assessment of the case will be requested from the reporter or other available sources.

10.5.4. Review and reporting of changes in the risk-benefit ratio

Without delay, and at the latest within 15 days of the decision for the need to do so, the sponsor will inform the ethics committee responsible and the competent authorities of all other member states of the EU or EEA where the trial is being conducted, of any events or factors that mean that the risk-benefit ratio of the study drug has to be reviewed. These consist of especially:

- individual reports of expected serious ADRs with an unexpected outcome

- a clinically relevant increase in the rate of occurrence of expected SADRs
- SUSARs in trial patients who have already completed the follow-up period of the clinical trial (”end-of-trial visit”)
- factors emerging in connection with trial conduct or the development of the study drug that may affect the safety of persons concerned.

10.5.5. Informing the investigators

The GCLLSG informs investigators of all SUSARs including all relevant further information within the periods set by the supreme federal authority. If new information becomes known that is different from the scientific information given to the investigator, all investigators will be informed by the GCLLSG.

10.5.6. Informing the marketing authorization holder

The GCLLSG will also inform the marketing authorization holder about all SAEs and SUSARs including information reported to the competent supreme authority and ethics committee in accordance with contractual agreements. Details of this reporting will be covered in a contract between the Sponsor and the company.

10.5.7. Development Safety Update report of trial patients

Once per year or on demand, the GCLLSG will supply a report on the safety of all patients in the trial in accordance with GCP §13 and ICH E2F guideline “Note for guidance on development safety update reports” with all relevant information during the reference period to the competent supreme federal authority and the leading ethics committee responsible. The GCLLSG will supply the report within 60 days of one year after the reference date (data-lock point).

10.6. SAE reporting after the end of the clinical trial

The investigator is not required to actively monitor patients after the study has ended. According to section 3.E.3 „Post study events” of the ICH E2A-guideline the investigator may report adverse events he evaluated as related to the participation in that trial to the sponsor.

Although such information is not routinely sought or collected by the sponsor, SAE that occurred after the patient had completed a clinical study (including any protocol-required post-treatment follow-up) will possibly be reported by an investigator to the sponsor. Such cases should be regarded for expedited reporting purposes as though they were study reports. Therefore, a causality assessment and determination of expectedness are needed for a decision on whether or not expedited reporting is required. The investigator may report such late adverse events on the regular SAE reporting form of this study. If not available, he should contact the study office of the GCLLSG to be provided with an adequate SAE form.

11. Data quality assurance

11.1. Monitoring

The trial sites will be monitored to ensure the quality of the data collected. The objectives of the monitoring procedures are to ensure that the patient´s safety and rights as a study participant are respected, that accurate, valid and complete data are collected, and that the trial is conducted in accordance with the trial protocol, the principles of GCP and local legislation.

The exact extent of the monitoring procedures is described in a separate monitoring manual. In principle, there will be an initiation visit before a patient may be recruited at each particular site. Afterwards there will be regular monitoring visits dependent on recruitment.

All investigators agree that the monitor regularly visits the trial site and assure that the monitor will receive appropriate support in their activities at the trial site, as agreed in separate contracts with each trial site. The declaration of informed consent (see Section *13.3 Obtaining informed consent*) includes a statement to the effect that the monitor has the right – while observing the provisions of data protection legislation – to compare the case report forms (CRFs) with the trial subject's medical records (doctor's notes, ECGs, laboratory printouts etc.). The investigator will secure access for the monitor to all necessary documentation for trial-related monitoring. The aims of the monitoring visits are as follows:

- check the declarations of informed consent
- monitor trial subject safety (occurrence and documentation/reporting of AEs and SAEs)
- check the completeness and accuracy of entries on the CRFs
- validate the entries on the CRFs against those in the source documents (source data verification, SDV),
- perform drug accountability checks
- evaluate the progress of the trial
- evaluate compliance with the trial protocol
- assess whether the trial is being performed according to GCP at the trial site
- discuss with the investigator aspects of trial conduct and any deficiencies found

A monitoring visit report is prepared for each visit describing the progress of the clinical trial and any problems (e.g. refusal to give access to documentation).

11.1.1. Audits/Inspections

As part of quality assurance, the sponsor has the right to audit the trial sites and any other institutions involved in the trial. The aim of an audit is to verify the validity, accuracy and completeness of data, to establish the credibility of the clinical trial, and to check whether the patient's rights and trial subject safety are being maintained. The sponsor may assign these activities to persons otherwise not involved in the trial (auditors). These persons are allowed access to all trial documentation (especially the trial protocol, case report forms, patients' medical records, drug accountability documentation, and trial-related correspondence).

The sponsor and all trial sites involved undertake to support auditors and inspections by the competent authorities at all times and to allow the persons charged with these duties access to the necessary original documentation.

All persons conducting audits will make all efforts to keep all patients´ data and other trial information confidential.

11.2. Documentation

All patient-related data will be recorded in a pseudonymized way. Each patient will be unequivocally identified by a trial subject number, attributed at recruitment into the study. The investigator is required

to keep a patient identification log, including the full name and address of the patient and eventually additional relevant personal data such as hospital record number, home physician etc.

All data entries during the study should be performed by authorized, trained site staff. All recorded data are to be plausible and complete.

The investigator has to review all pages within the CRF for accuracy and consistency with the protocol and sign and date the CRF pages with a secure signature upon completion. Furthermore the Principal Investigator or Deputy Principal Investigator has to confirm, to the best of their knowledge, the accuracy, correctness and completeness of the data documented for the patient with date and signature.

The study office and / or monitor checks the entered data for completeness and plausibility. In case of any discrepancies, queries will be raised.

All data entered on the eCRF must be documented in a source document. The CRF is not a source document.

CT scans have to be reported in summary in the CRF. The original reports, traces and films must be retained by the investigator for future reference.

11.3. Data management

The GCLLSG study office is responsible for the data management and for the selection of a clinical database. All changes made to the data in the clinical database are documented in an audit trail. The software has a user and role concept that can be adjusted on a trial-specific basis. State of the art security and backup strategies are implemented.

The trial database will be tested before data entry starts. A data management plan will be created before data collection begins and will describe all specifications for data collection, data handling, data validation and the cleaning process. These data quality procedures are in compliance with the protocol, GCP, and any applicable regulatory requirements.

11.4. Archiving

All CRFs and the complete trial master file, including other important trial materials will be archived for at least 10 years from the end of the clinical trial in accordance with §13 Sec. 10 of the GCP Regulations. Trial subject identification lists at each trial site will be stored separately from trial documentation.

12. Closure of trial sites/Premature termination of the clinical trial

12.1. Closure of trial sites

The sponsor and the sponsor's representative have the right to discontinue a single site at any time during the study for reasonable medical or administrative reasons.

Possible reasons for trial site closure could be, but are not limited to:

- unsatisfactory enrolment with respect to quantity or quality
- inaccurate or incomplete data collection
- non-compliance with GCP
- failure to adhere to the study protocol

12.2. Premature termination of trial

The sponsor has the right to terminate the trial prematurely if there are any relevant medical or ethical concerns, or for reasonable administrative reasons. If such action is taken, the reasons for terminating the trial must be documented in detail. All patients still under treatment at the time of termination must undergo a final staging, which must be documented. The Global Principal Investigator must be informed without delay if any investigator has ethical concerns about continuation of the trial.

12.2.1. Stopping rules for the trial

The sponsor has the right to terminate the trial prematurely if there are any relevant medical or ethical concerns, or for reasonable administrative reasons. If such action is taken, the reasons for terminating the trial have to be documented in detail. All patients who are not considered end of study must undergo a final examination, which must be documented.

Criteria for termination of the study as a whole are:

- An unacceptable safety profile revealed by the interim safety analysis of this trial (to be performed by the GPI together with the coordinating physician, one statistician and other members of the protocol committee as soon as six patients have completed three cycles of therapy), see chapter *7.2 42*
- An unacceptable profile or incidence rate of adverse events/ adverse events of special interest revealed in this or any other study in which at least one of the investigational products of this trial is administered.
- Demonstration that the study treatment is ineffective or only insufficiently active.
- Significant number of cases of death associated with the study treatment.
- Any other factor that in the view of the sponsor constitutes an adequate reason for terminating the study as a whole.

The GPI has to be informed without delay if any investigator has ethical concerns.

13. Ethical and regulatory aspects

13.1. Ethical considerations

The Sponsor and all investigators agree to conduct this study in accordance with the protocol and with consensus ethical principles derived from international guidelines including the Declaration of Helsinki and Council for International Organizations of Medical Sciences (CIOMS) International Ethical Guidelines, applicable ICH Good Clinical Practice (GCP) Guidelines and applicable laws and regulations.

The participating investigators/institutions permit trial-related monitoring, audits, IRB/IEC review and regulatory inspections and provide direct access to source documents/data. The investigator assumes the responsibility of obtaining written informed consent for each patient before any study-specific procedures are performed and before any study drug is administered. If an amendment to the protocol changes the risk participation schedule in scope or activity, or increases the potential risk to the subject, the informed consent document must be used to obtain re-consent from any patients currently enrolled in the study if the patient is affected by the amendment and must be used to document consent from any new patients enrolled after the approval date of the amendment. The Sponsor and the investigators affirm and uphold the principle of the patients' right to privacy. The investigators shall comply with applicable privacy laws. The investigator must assure that the patients' anonymity will be maintained and that the identities are protected from unauthorized parties. The investigator should maintain documents not for submission to the GCLLSG study office e.g. subjects written informed consent forms, in strict confidence.

All clinical and scientific data are collected under a unique code or "pseudonym" composed of an abbreviation of the study and a random string of 6 digits and is stored in the main clinical trial database. All data exchange with the study center is made solely via the unique code. All participating study centers are obliged to keep a secret trial subject identification list.

13.2. Good Clinical Practice and regulatory requirements

The protocol for this study has been designed in accordance with the general ethical principles outlined in the Declaration of Helsinki. The Sponsor will be responsible for preparing documents for submission to the relevant ethics committees (EC) as well as the Clinical Trial Application to the appropriate Health Authorities and obtaining written approval of both the EC and the appropriate Health Authorities for this study. The approval will be obtained prior to the initiation of the study.

The approval for both the protocol and informed consent must specify the date of approval, protocol number and version, or amendment number. Any amendments to the protocol after receipt of approval by the ethics committee (EC), Paul-Ehrlich-Institut (PEI) must be submitted by the Sponsor to the EC as well as the appropriate Health Authorities for approval. The Sponsor is also responsible for notifying the EC as well as the PEI of any serious deviations from the protocol, or anything else that may involve added risk to patients. Any advertisements used to recruit patients for the study must be reviewed and approved by the EC prior to use. The participating investigators/institutions permit trial-related monitoring, audits, independent EC review, and regulatory inspections and provide direct access to source documents/data.

13.3. Obtaining informed consent

Patients may not be enrolled into the trial unless they have consented to take part in the trial after having been informed verbally and in writing in comprehensible language of the nature, scope and possible consequences by a trial investigator. Together with the consent to take part in the trial, the trial subject must also agree to representatives of the sponsor (e.g. monitors or auditors) or the competent supervisory or federal authorities having access to the data recorded within the framework of the clinical trial.

The trial subject will be informed of the potential benefit and possible side effects of the Investigational Medicine Product (IMP), of the need and reasons to conduct a clinical trial, and the nature of the therapy, the investigational status of the study drug, and other factors that are part of obtaining a proper informed consent. It must be clear to each patient that he/she can withdraw his/her consent at any time without giving reasons and without jeopardizing his/her further course of treatment. Patients will be given the opportunity to ask questions concerning the study, and adequate time to consider their decision whether to participate or not.

Patients meeting the criteria set forth in the protocol will be offered the opportunity to participate in the study. To avoid introduction of bias, the investigator must exercise no selectivity with regard to offering all eligible patients the opportunity to participate in the study. Documentation that informed consent using of a written consent form that includes all the elements required by regulations and ICH guidelines occurred prior to the patient's entry into the study and of the informed consent process should be recorded in the patient's source documents. The original consent form signed and dated by the patient and by the person consenting the patient prior to the patient's entry into the study must be maintained in the investigator's study files and a copy given to the patient. The date and time of the informed consent must additionally be recorded in the source documents. If a protocol is amended and it impacts on the content of the informed consent, the informed consent must be revised. All patients participating in the study when the amended protocol is implemented must be re-consented with the revised version of the informed consent form. However, patients in the follow-up phase, who have already stopped study treatment will not have to be re-consented if the changes of protocol and informed consent are not relevant for them anymore, e.g. change of risk profile of the study drugs with only acute and not late toxicities. The original amended consent form signed and dated by the patient and by the person consenting the subject must be maintained in the investigator's study files and a copy given to the patient.

Part of the monitoring activities are to check that the most recent informed consent form was used before the trial subject was enrolled and that it was dated and signed by the patient himself.

13.4. Insurance of patients in the trial

All patients enrolled are insured in accordance with § 40 AMG, details for the insurance are to be found in the general conditions of insurance. The headquarters, policy number and telephone and fax number of the respective insurance company will be included in the patients informed consent form.

13.5. Data protection

The investigator affirms and upholds the principle of the patients' right to privacy. The investigators shall comply with applicable privacy laws. The investigator must assure that the patient's anonymity will be maintained and that the identities are protected from unauthorized parties. Should direct access to medical records require a waiver or authorization separate from the patient's statement of informed consent, it is the responsibility of the investigator(s) to obtain such permission in writing from the appropriate individual.

The provisions of data protection legislation will be observed. It is assured by the sponsor that all investigational materials and data will be pseudonymized in accordance with data protection legislation before scientific processing. Patients will be informed that their pseudonymized data will be passed on in accordance with provisions for documentation and notification pursuant to § 12 and § 13 of the GCP Regulations to the recipients described there. Patients who do not agree that the information may be passed on in this way will not be enrolled into the trial. The investigator should maintain documents not for submission to the GCLLSG study office in strict confidence. On CRF's and other documents patients should not be identified by their names. All clinical and scientific data are collected under a unique identification code for each patient, and stored in the main clinical trial database. By keeping the infor-

mation separately from the main clinical trial database, it is ensured that one cannot draw any conclusions about the identity of the treated individual.

All data exchange with the study center is made solely via the unique identification code. All participating study centers are obliged to keep a secret subject identification list.

14. Statistical methods and sample size calculation

14.1. General consideration

Statistical analysis is the responsibility of the statisticians of the GCLLSG. The analyses outlined in this section will be reviewed and a final statistical analysis plan (SAP) will be written before any analysis is undertaken. The analysis plan will be finalized and agreed by the study management team. Any changes to the finalized plan, and reasons for changes, will be documented.

14.2. Sample size

The primary endpoint overall response rate (ORR) at end of induction therapy was used to determine the sample size of the study. The following study assumptions are considered for patients with at least one dose of study medication in the third induction cycle:

The investigated regimen is assessed to be not effective if the ORR is less than 40 % (=P0) with corresponding null hypothesis H0: ORR ≤ 0.4 and alternative hypothesis H1: ORR > 0.4. This boundary of efficacy of 40 % ORR corresponds to response rates observed in RS patients treated with conventional chemoimmunotherapy.

The investigated regimen is considered potentially useful and worthy of further research if we can reject the null hypothesis in favor of the alternative hypothesis. It is assumed to improve the ORR to at least 60 % (=P1) with the investigated regimen.

A one-sided one-sample binomial-test with an overall significance level of 2.5 % provides the sample size N=48 for patients with at least one dose of study medication in the third induction cycle, such that statistical significance is achieved with a power of 1-β=80 % at the assumed ORR 60 %. Thus, patients discontinuing study therapy before receiving study medication in the third induction cycle do not count for the target sample size of N=48 and should be replaced.

The following table describes the minimum number of responders (i.e. having a CR or PR) that are required to warrant further investigation of the new regimen based on different numbers of analyzable patients (i.e. patients having received at least one dose of study medication in the third induction cycle):

Number of analyzable patients	Minimum number of responders
51, 50	27
49, 48	26
47, 46	25
45, 44, 43	24
42, 41	23
40, 39	22
38, 37	21
36	20

Sample size calculations were performed with EAST 5 software and validated with Binomial tables.

14.3. Trial populations

For the statistical analyses, the following patient populations will be defined:

The target population for the primary and secondary efficacy endpoints is the **full analysis set (FAS)**. This dataset includes all patients enrolled into the trial who received at least two complete cycles of induction therapy, i.e. who received at least one dose of study medication in the third cycle of induction therapy.

All safety parameters will be analyzed on the **safety population (SP)**. This population includes all patients who have received at least one dose of any substance of trial treatment, whether withdrawn prematurely or not.

The allocation to the different populations (FAS, SP) will be determined for every patient according to the previously defined criteria. The number of patients constituting the FAS and the SP will be reported as well as the reasons for exclusions of patients from a certain population.

14.4. General study overview

14.4.1. Conduct of study

Enrollment, major protocol violations, early discontinuations from study treatment (including reasons), and early study withdrawals (including reasons) will be summarized and described. Protocol violations being assessed as major will be listed in the SAP.

14.4.2. Observation time

To assess the observation time of the study the reverse Kaplan-Meier survival methodology will be applied to the continuous overall survival variable referring to the difference between registration and death or last information the patient was known to be alive in months (according to the definition of overall survival). Median observation time (including interquartile range, minimum, and maximum) will be estimated with respect to the FAS.

14.4.3. Demographic and baseline characteristics

Demographic and baseline characteristics at screening will be presented for the FAS. A complete list of parameters will be described in the SAP. Descriptive statistics for continuous variables including median, interquartile range, mean, minimum, maximum and standard deviation will be used. Additionally, selected continuous variables will be dichotomized according pre-defined cut-off values. Categorical variables will be reported with relative frequencies. Moreover, demographic and baseline characteristics will be reported regarding to the subgroup of patients for whom a respective parameter is available. Thereto the number of patients available and the proportion of patients for whom data are missing will be described with respect to the FAS.

Further analyses will refer to the evaluation of previous therapies including type and number of previous therapies.

14.4.4. Primary endpoint

The primary efficacy variable (primary endpoint) is the overall response rate (ORR) at interim staging after end of induction therapy (end of induction treatment response = EOIT). ORR is defined as the proportion of patients having achieved a CR or PR (according to the refined Lugano Classification) based on the FAS. Patients without any documented response assessment will be kept and labeled as 'non-responder' in the analysis.

14.4.5. Analysis of primary endpoint

The primary efficacy analysis will be performed with a single stage phase II design with respect to the

FAS. The ORR of the study treatment will be compared with the benchmark of P0 = 40 % using a one-sided one-sample binomial test. Statistical significance will be defined as $p < 0.025$. The efficacy of the study treatment will be concluded if the null hypothesis is rejected.

The relative frequency of patients having responded to therapy at interim staging after induction therapy including the corresponding 95 % Clopper Pearson confidence interval will be reported with respect to the FAS.

14.5. Secondary efficacy endpoints

Analyses of secondary efficacy endpoints will be not tested formally and there is no control of type I error of any study endpoint. Every endpoint will be descriptively analyzed and reported. Analyses will be based on the FAS.

Rate based endpoints shall be assessed showing frequencies and corresponding percentages including 95 % Clopper Pearson confidence intervals.

Analyses of time-to-event endpoints will be performed using Kaplan-Meier methods with the Kaplan-Meier survival curve presented to provide a visual description. Kaplan-Meier estimates of median time and rates for 6, 12 and 24 months after registration will be reported. A scheme for determination of date of censoring will be provided in the SAP.

14.5.1. Response rates

14.5.1.1. Overall response rate (ORR) after induction therapy according to IWCLL criteria

The overall response rate (ORR) at interim staging after end of induction therapy (EOIT response, i.e. after 6 cycles of therapy) according to IWCLL criteria will be defined as the proportion of patients having achieved a CR CRi or PR based on the FAS. Patients without any documented response assessment will be kept and labeled as ‘non-responder' in the analysis.

14.5.1.2. Overall response rate (ORR) after consolidation therapy

The overall response rate (ORR) after consolidation therapy (i.e. after 12 cycles of therapy) will be calculated with respect to the population of patients having received any dose of consolidation treatment. It will be evaluated both according to the refined Lugano Classification as well as according to the IWCLL criteria. In the first case it will be defined as the proportion of patients having achieved a CR or PR as best response until and including the last response assessment after end of consolidation therapy. In the second case, it will be defined as the proportion of patients having achieved a CR, CRi, or PR as best response until and including the last response assessment after end of consolidation therapy.

14.5.2. Time-to-event endpoints

14.5.2.1. Progression-free survival (PFS)

Progression-free survival (PFS) will be measured from the date of registration to the date of first occurrence of disease progression or relapse (determined according to the IWCLL guidelines and Lugano Classification) or death from any cause, whichever occurs first. These will be counted as events for PFS. Start of a subsequent CLL/RT treatment after the study treatment will not be counted as an event. Patients for whom no documented event for PFS is available at the time of analysis will be censored at the time point of last observation they were assessed to be event-free.

14.5.2.2. Overall survival (OS)

Overall survival (OS) will be measured from the date of registration to the date of death due to any cause. Patients who have not yet died at the time of analysis will be censored at the time of last observation they were assessed to be alive.

14.5.2.3. Duration of response

Duration of response will be evaluated both according to the refined Lugano Classification as well as according to the IWCLL criteria. In the first case it will be calculated for patients with CR or PR as EOIT response according to the definition of the primary endpoint, in the second case for patients with CR, CRi or PR as EOIT response. Duration of response will be measured from the date of first documented response to the first occurrence of progression, relapse or death by any cause, whichever occurs first. These will be counted as event for duration of response. Patients for whom no documented event for duration of response is available at the time of analysis will be censored at the time of last observation they were assessed to be event-free.

14.5.2.4. Time to next treatment (TTNT)

Time to next treatment (TTNT) will be measured from date of registration to the date of first subsequent CLL/RT treatment. These will be counted as event for TTNT. Patients for whom no subsequent CLL/RT treatment is documented will be censored at the time of last observation they were assessed to be free from subsequent treatment.

14.6. Safety analyses

Safety parameters include treatment exposure, adverse events (including adverse events of special interest and adverse events of particular interest) and death cases. Analysis of safety parameters will be performed on the SP unless stated otherwise.

14.6.1. Treatment exposure

Summaries regarding treatment exposure by patient (based on the FAS) will include the number of cycles received, cumulative doses, dose modifications and dose intensity. In particular, the proportion of patients having received SCT for consolidation will be evaluated.

14.6.2. Adverse events

Adverse events (AE) will be classified using the Medical Dictionary for Regulatory Activities (MedDRA) classification system. AEs will be reported by MedDRA system organ class (SOC), high level term (HLT), high level group term (HLGT), and preferred term (PT). The severity of the AEs will be graded according to the recent updated NCI CTCAE version 5.

First, all cases of AEs will be reported in a case analysis including the number of events classified as being serious and related to study drugs (according to the investigator). Specifications of AEs leading to death or early treatment discontinuation will also be summarized.

Second, AEs will be reported in a by-patient analysis. This means, that an event will be counted once only (with worst NCI CTCAE) if a subject has the same event more than once. Regarding the severity frequency tables will be based on all AEs classified as NCI CTCAE Grade 1-5 as well as AEs classified as NCI CTCAE Grade 3-5.

14.6.3. Mortality

With regard to overall survival, the causes of death will be reported. The treatment related mortality will be calculated as the number of treatment-related deaths divided by the SP.

14.6.4. Exploratory analyses

Exploratory analyses will be part of the secondary endpoint analyses and may be performed after or at the final analysis of the primary endpoint. Results of exploratory analyses might be reported separately from the analyses regarding primary and secondary endpoints and safety analyses. Analyses might be described in a separate SAP. Analyses will be performed on the FAS.

As for exploratory endpoints the correlations between baseline markers and clinical outcome will be analyzed. Response rates and time-to-event endpoints will be considered as outcome parameters. Baseline markers to be considered include clinical [age, gender, comorbidity status, number of previous therapies, etc], laboratory [hematological and serum parameters] and biological [PD-1/PD-L1 expression, cytogenetics, TP53 mutational status, IGHV mutational status, etc.] parameters. A complete list will be specified in the SAP.

14.7. Analysis time-points

The primary endpoint can be analysed as soon as all enrolled patients who did not discontinue prematurely have completed the interim staging after 6 cycles of induction therapy. This will trigger the time point of the analysis of the primary and secondary endpoints.

Safety analyses are independent from primary and secondary efficacy analyses.

15. Final report and publication

15.1. Final report

The responsible competent authority and the ethics committees will be informed within 90 days that the trial has officially ended.

Within one year of the completion of the trial, the competent authorities and the ethics committees will be supplied with a summary of the final report on the clinical trial containing the principle results.

15.2. Publication

It is planned to publish the trial results in a scientific journal and present results at German and international conferences. The trial was already registered in a public register. Any published data will observe data protection legislation covering the trial subject and investigators. Response rates or individual findings at individual trial sites are known only to the sponsor. Publications or lectures on the findings of the present clinical trial either as a whole or at individual investigation sites must be reviewed by the sponsor in advance.

By signing the contract to participate in this trial, the investigator declares that he/she agrees to submission of the results of this trial to national and international authorities for approval and surveillance purposes, and to the Federal Physicians Association, the Association of Statutory Health Fund Physicians and to statutory health fund organizations, if required. At the same time, the investigator agrees that his or her name, address, qualifications and details of his or her involvement in the clinical trial may be made known to these bodies.

16. Amendments to the trial protocol

Any modifications to the protocol which may have an impact on the conduct of the study or the potential benefit of the study, or which may affect patient´s safety – including changes of study objectives, study design, subject population, sample sizes, study procedures, or significant administrative aspects (cf. § 10, Abs. 1 GCP-V for the decision criteria) – will require a formal written amendment to the protocol. Such an amendment will be agreed upon by the sponsor. It requires a new application to the responsible authority and to the responsible ethics committee before implementation, according to §10, Abs. 2 to 4 GCP-V.

Administrative or technical changes of the protocol such as minor corrections and/or clarifications that neither have effect on the way the study is to be conducted, nor on the risk–benefit ratio, will be agreed upon by the sponsor and the investigator(s) and will be documented in a memorandum to the protocol. The ethics committee responsible may be notified of such changes at the discretion of the sponsor.

The sponsor has to assure, that all amendments have been added to the study documents at any site involved in the trial.

17. Appendices

A. Central laboratory assessment
B. G8 geriatric assessment

18. References

1. Jain, P. and S. O'Brien, *Richter's transformation in chronic lymphocytic leukemia.* Oncology (Williston Park), 2012. **26**(12): p. 1146-52.
2. Tsimberidou, A.M., M.J. Keating, and W.G. Wierda, *Richter's transformation in chronic lymphocytic leukemia.* Curr Hematol Malig Rep, 2007. **2**(4): p. 265-71.
3. Jain, N. and M.J. Keating, *Richter transformation of CLL.* Expert Rev Hematol, 2016. **9**(8): p. 793-801.
4. Constantine S. Tam, S.O., Gavin Cull, Judith Trotman, David Gottlieb, David Simpson, Paula Marlton, Mary Ann Anderson, Matthew Ku, David Ritchie, Sumita Ratnasingam, Bradley Augustson, Mark Kirschbaum, Lai Wang, Ling Xue, Jianxin Yang, Eric Hedrick, John F Seymour and Andrew W. Roberts, *Twice Daily Dosing with the Highly Specific BTK Inhibitor, Bgb-3111, Achieves Complete and Continuous BTK Occupancy in Lymph Nodes, and Is Associated with Durable Responses in Patients (pts) with Chronic Lymphocytic Leukemia (CLL)/Small Lymphocytic Lymphoma (SLL)*, in *ASH Meeting*. 2016: San Diego.
5. Gavin Cull, S.O., Judith Trotman, James Hilger, Xiaoping Zhang, Shibao Feng, Sunhee Ro, Jane Huang, and Constantine S. Tam, *Safety and Activity of the Highly Specific BTK Inhibitor BGB-3111 in Combination with the PD-1 Inhibitor BGB-A317 in Patients with B-Cell Lymphoid Malignancies*, in *ASH Meeting*. 2017: Atlanta.
6. Jain, N., et al., *Nivolumab Combined with Ibrutinib for CLL and Richter Transformation: A Phase II Trial.* Blood, 2016. **128**(22): p. 59-59.
7. Anas Younes, J.B., Cecilia Carpio, Armando Lopez-Guillermo, Dina Ben-Yehuda, A. Burhan Ferhanoglu, Arnon Nagler, Muhit Ozcan, Irit Avivi, Francesc Bosch Albareda, María Dolores Caballero Barrigón, Aisha Masood, Michael Streit, John Alvarez, Rob Ceulemans, Behzad Kharabi Masouleh, Sriram Balasubramanian, Michael Schaffer, Shean-Sheng Wang, Nele Fourneau,* and Wojciech Jurczak, *Safety and Efficacy of the Combination of Ibrutinib and Nivolumab in Patients with Relapsed Non-Hodgkin Lymphoma or Chronic Lymphocytic Leukemia*, in *ASH Meeting*. 2017: Atlanta.
8. Nitin Jain, S.B., Philip A Thompson, Maro Ohanian, Alessandra Ferrajoli, Naveen Pemmaraju, Jorge E. Cortes, Zeev Estrov, Jan A Burger, Sattva S. Neelapu, Wanda Lopez, Beenu Thakral, Carlos E. Bueso-Ramos, Jorge Blando, Susan M. O'Brien, Hagop M. Kantarjian, James Allison, Michael Keating, Padmanee Sharma and William G. Wierda, *Nivolumab Combined with Ibrutinib for CLL and Richter Transformation: A Phase II Trial*, in *ASH Meeting*. 2016: San Diego.
9. Albi, E., et al., *Ibrutinib treatment of a patient with relapsing chronic lymphocytic leukemia and sustained remission of Richter syndrome.* Tumori, 2017. **103**(Suppl. 1): p. e37-e40.
10. Hillmen, P., et al., *Acalabrutinib Monotherapy in Patients with Richter Transformation from the Phase 1/2 ACE-CL-001 Clinical Study.* Blood, 2016. **128**(22): p. 60-60.
11. Master, S., et al., *Successful Treatment of Richter Transformation with Ibrutinib in a Patient with Chronic Lymphocytic Leukemia following Allogeneic Hematopoietic Stem Cell Transplant.* Case Rep Oncol, 2017. **10**(2): p. 534-541.
12. Cheson, B.D., et al., *Refinement of the Lugano Classification lymphoma response criteria in the era of immunomodulatory therapy.* Blood, 2016. **128**(21): p. 2489-2496.
13. Hallek, M., et al., *Guidelines for diagnosis, indications for treatment, response assessment and supportive management of chronic lymphocytic leukemia.* Blood, 2018.

14. Howlader, N., et al., *SEER Cancer Statistics Review, 1975-2010.* National Cancer Institute. Bethesda, MD, http://seer.cancer.gov/csr/1975_2010/ on 31.5.2013 15:08, based on November 2012 SEER data submission, posted to the SEER web site, April 2013., 2013.

15. Jemal, A., et al., *Cancer statistics, 2009.* CA Cancer J Clin, 2009. **59**(4): p. 225-49.

16. Müller-Hermelink, H.K., et al., *Chronic lymphocytic leukaemia/small lymphocytic lymphoma*. 4th ed. WHO Classification of Tumours of Haematopoietic and Lymphoid Tissues, ed. S.H. Swerdlow, et al. 2008, Lyon.

17. Rozman, C. and E. Montserrat, *Chronic lymphocytic leukemia.* N Engl J Med, 1995. **333**(16): p. 1052-7.

18. Hallek, M., et al., *Guidelines for the diagnosis and treatment of chronic lymphocytic leukemia: a report from the International Workshop on Chronic Lymphocytic Leukemia updating the National Cancer Institute-Working Group 1996 guidelines.* Blood, 2008. **111**(12): p. 5446-56.

19. Ginaldi, L., et al., *Levels of expression of CD19 and CD20 in chronic B cell leukaemias.* J Clin Pathol, 1998. **51**(5): p. 364-9.

20. Moreau, E.J., et al., *Improvement of the chronic lymphocytic leukemia scoring system with the monoclonal antibody SN8 (CD79b).* Am J Clin Pathol, 1997. **108**(4): p. 378-82.

21. Burger, J.A., et al., *The microenvironment in mature B-cell malignancies: a target for new treatment strategies.* Blood, 2009. **114**(16): p. 3367-75.

22. Wiestner, A., *Emerging role of kinase-targeted strategies in chronic lymphocytic leukemia.* Blood, 2012. **120**(24): p. 4684-91.

23. Cleary, M.L., S.D. Smith, and J. Sklar, *Cloning and structural analysis of cDNAs for bcl-2 and a hybrid bcl-2/immunoglobulin transcript resulting from the t(14;18) translocation.* Cell, 1986. **47**(1): p. 19-28.

24. Tsujimoto, Y., et al., *Cloning of the chromosome breakpoint of neoplastic B cells with the t(14;18) chromosome translocation.* Science, 1984. **226**(4678): p. 1097-9.

25. Gribben, J.G., *How I treat CLL up front.* Blood, 2010. **115**(2): p. 187-97.

26. Binet, J.L., et al., *Perspectives on the use of new diagnostic tools in the treatment of chronic lymphocytic leukemia.* Blood, 2006. **107**(3): p. 859-61.

27. Binet, J.L., et al., *A new prognostic classification of chronic lymphocytic leukemia derived from a multivariate survival analysis.* Cancer, 1981. **48**(1): p. 198-206.

28. Rai, K.R., et al., *Clinical staging of chronic lymphocytic leukemia.* Blood, 1975. **46**(2): p. 219-34.

29. Dohner, H., et al., *Genomic aberrations and survival in chronic lymphocytic leukemia.* N Engl J Med, 2000. **343**(26): p. 1910-6.

30. Damle, R.N., et al., *Ig V gene mutation status and CD38 expression as novel prognostic indicators in chronic lymphocytic leukemia.* Blood, 1999. **94**(6): p. 1840-7.

31. Hamblin, T.J., Z.A. Davis, and D.G. Oscier, *Determination of how many immunoglobulin variable region heavy chain mutations are allowable in unmutated chronic lymphocytic leukaemia - long-term follow up of patients with different percentages of mutations.* Br J Haematol, 2008. **140**(3): p. 320-3.

32. Zenz, T., et al., *Monoallelic TP53 inactivation is associated with poor prognosis in chronic lymphocytic leukemia: results from a detailed genetic characterization with long-term follow-up.* Blood, 2008. **112**(8): p. 3322-9.

33. group, I.C.-P.w., *An international prognostic index for patients with chronic lymphocytic leukaemia (CLL-IPI): a meta-analysis of individual patient data.* Lancet Oncol, 2016. **17**(6): p. 779-790.

34. Dal Porto, J.M., et al., *B cell antigen receptor signaling 101.* Mol Immunol, 2004. **41**(6-7): p. 599-613.

35. Niiro, H. and E.A. Clark, *Regulation of B-cell fate by antigen-receptor signals.* Nat Rev Immunol, 2002. **2**(12): p. 945-56.

36. Rickert, R.C., *New insights into pre-BCR and BCR signalling with relevance to B cell malignancies.* Nat Rev Immunol, 2013. **13**(8): p. 578-91.

37. Conley, M.E., et al., *Primary B cell immunodeficiencies: comparisons and contrasts.* Annu Rev Immunol, 2009. **27**: p. 199-227.

38. Burger, J.A. and A. Wiestner, *Targeting B cell receptor signalling in cancer: preclinical and clinical advances.* Nat Rev Cancer, 2018. **18**(3): p. 148-167.

39. Pal Singh, S., F. Dammeijer, and R.W. Hendriks, *Role of Bruton's tyrosine kinase in B cells and malignancies.* Mol Cancer, 2018. **17**(1): p. 57.

40. Tam, C.S., et al., *Safety and Activity of the Highly Specific BTK Inhibitor BGB-3111 in Patients with Indolent and Aggressive Non Hodgkin's Lymphoma.* Blood, 2017. **130**(Suppl 1): p. 152-152.

41. McDermott, D.F. and M.B. Atkins, *PD-1 as a potential target in cancer therapy.* Cancer Med, 2013. **2**(5): p. 662-73.

42. Ribas, A. and J.D. Wolchok, *Cancer immunotherapy using checkpoint blockade.* Science, 2018. **359**(6382): p. 1350-1355.

43. Merryman, R.W., et al., *Checkpoint blockade in Hodgkin and non-Hodgkin lymphoma.* Blood Adv, 2017. **1**(26): p. 2643-2654.

44. Ding, W., et al., *PD-1 Blockade with Pembrolizumab in Relapsed CLL Including Richter's Transformation: An Updated Report from a Phase 2 Trial (MC1485).* Blood, 2016. **128**(22): p. 4392-4392.

45. Sagiv-Barfi, I., et al., *Therapeutic antitumor immunity by checkpoint blockade is enhanced by ibrutinib, an inhibitor of both BTK and ITK.* Proceedings of the National Academy of Sciences, 2015. **112**(9): p. E966-E972.

46. Smith, T., et al., *2006 update of recommendations for the use of white blood cell growth factors: an evidence-based clinical practice guideline.* J Clin Oncol, 2006. **24(19):3187-205**.

47. Noseworthy, P.A., et al., *Direct Comparison of Dabigatran, Rivaroxaban, and Apixaban for Effectiveness and Safety in Nonvalvular Atrial Fibrillation.* CHEST, 2016. **150**(6): p. 1302-1312.

48. Postow, M.A., *Managing immune checkpoint-blocking antibody side effects.* Am Soc Clin Oncol Educ Book, 2015: p. 76-83.

49. Othman, J., et al., *Severe hemolysis and transfusion reactions after treatment with BGB-3111 and PD-1 antibody for Waldenstrom macroglobulinemia.* Haematologica, 2018. **103**(5): p. e223-e225.

50. Thorp, B.C. and X. Badoux, *Atrial fibrillation as a complication of ibrutinib therapy: clinical features and challenges of management.* Leuk Lymphoma, 2018. **59**(2): p. 311-320.

51. Cheson, B.D., et al., *Recommendations for initial evaluation, staging, and response assessment of Hodgkin and non-Hodgkin lymphoma: the Lugano classification.* J Clin

Oncol, 2014. **32**(27): p. 3059-68.

52. Maurer, C., et al., *Effect of first-line treatment on second primary malignancies and Richter's transformation in patients with CLL.* Leukemia, 2016. **30**(10): p. 2019-2025.

CLL-RT1 STATISTICAL ANALYSIS PLAN (SAP)

Primary Endpoint Analysis

CLL-RT1 Protocol of the German CLL-Study Group

A prospective, open-label, multicenter phase-II trial to evaluate the efficacy and safety of zanubrutinib (BGB-3111), a BTK inhibitor, plus tislelizumab (BGB-A317), a PD1 inhibitor, for treatment of patients with Richter Transformation (CLL-RT1-trial of the GCLLSG)

Trial protocol code: CLL-RT1
EudraCT number: 2018-002492-17
Protocol version: 11.01.2023, version v4.0

**Version**	v2.0	**Effective from**	13.11.2023
**Authors**	Dr. Sandra Robrecht (ST-FL)	**Date**<br>**Signature**	
	Dr. Rudy Ligtvoet (ST)	**Date**<br>**Signature**	
**Approved by**	Dr. Othman Al-Sawaf (CP)	**Date**<br>**Signature**	
	Prof. Dr. Barbara Eichhorst (GPI)	**Date**<br>**Signature**	

Table of Content

1 Glossary of abbreviations and definitions ... 3
2 Scope of this document ... 4
3 Study design ... 4
3.1 Study design ... 4
3.2 Study objectives ... 4
3.3 Study endpoints ... 5
3.3.1 Primary endpoint ... 5
3.3.2 Secondary endpoints ... 5
3.3.3 Exploratory endpoints ... 5
3.4 Study assumptions and sample size calculation ... 5
4 Study populations ... 6
4.1 Inclusion criteria ... 6
4.2 Exclusion criteria ... 6
4.3 Full analysis set ... 7
4.4 Safety population ... 7
4.5 Intention to treat population ... 7
5 Analysis time-point ... 7
6 Study population and study endpoints evaluated in primary endpoint analysis ... 8
7 Definition of endpoints ... 8
7.1 Definition of rate-based endpoints ... 9
7.1.1 Overall response rate (ORR) after induction therapy according to Lugano Classification ... 9
7.1.2 Overall response rate (ORR) after induction therapy according to IWCLL criteria ... 9
7.2 Definition of time-to-event endpoints ... 9
7.2.1 Progression-free survival (PFS) ... 9
7.2.2 Duration of response (DOR) ... 10
7.2.3 Overall survival (OS) ... 10
7.2.4 Time to next CLL treatment (TTNT) ... 11
7.2.5 Modified time to next CLL treatment (modified TTNT) ... 12
8 Statistical analyses ... 12
8.1 Description of basic information ... 12
8.1.1 Description of study populations ... 12
8.1.2 Observation time ... 12
8.1.3 Demographic and baseline characteristics ... 13
8.2 Primary efficacy analysis ... 14
8.3 Analyses of secondary endpoints ... 14
8.4 Safety analysis ... 14
8.5 Treatment exposure ... 15
8.6 Exploratory analyses ... 16
9 Handling of missing data ... 17
10 Preparation of datasets ... 17
11 Attachments ... 18
12 Revision History ... 18

1 Glossary of abbreviations and definitions

Abbreviation	Meaning
AE	Adverse event
AESI	Adverse event of special interest
ATT	Attachment
BTK	Bruton's tyrosine kinase
CI	Confidence interval
CIRS	Cumulative Illness Rating Scale
CLL	Chronic lymphocytic leukaemia
CP	Coordinating physician
CR	Complete response
CRi	CR with incomplete recovery of the bone marrow
CTC	Common Toxicity Criteria
DLBCL	Diffuse large B cell lymphoma
DNA	Deoxyribonucleic acid
DOR	Duration of response
ECOG	Eastern Cooperative Oncology Group
FL	Functional Lead
FAS	Full analysis set
GCLLSG	German CLL Study Group
GPI	Global principal investigator
HBV	Hepatitis B virus
HBsAg	Hepatitis B surface antigen
HIV	Human Immunodeficiency virus
HL	Hodgkin's lymphoma
HLT	High level term according to MedDRA
HLGT	High level group term according to MedDRA
ICH	International Conference on Harmonization
IPI	International prognostic index
ITT	Intention to treat
MedDRA	Medical dictionary for regulatory activities
NCI	National Cancer Institute
ORR	Overall response rate
OS	Overall survival
PCR	Polymerase chain reaction
PD	Progression disease
PD1	Programmed cell death-1
PFS	Progression-free survival
PML	Progressive multifocal leukoencephalopathy
PR	Partial response
PT	Preferred term according to MedDRA
RNA	Ribonucleic acid
RT	Richter transformation
SAE	Serious adverse event
SAP	Statistical analysis plan
SCT	Stem cell transplantation
SOC	System Organ Class according to MedDRA
SP	Safety population
ST	Statistician, Statistics
TTNT	Time to next CLL treatment
ULN	Upper limit of normal

2 Scope of this document

This document is based on the statistical section of the CLL-RT1 protocol and serves as a technical complement.

The current version of the protocol (CLL-RT1_Protocol_version_4.0_ 20230111) foresees a final analysis of the primary endpoint as soon as all patients have reached the final restaging. This will trigger the time point of primary endpoint analysis. The scope of this document is to describe the analyses to be performed for the primary endpoint analysis.

The first six patients were part of an interim safety analysis (see section 7.2 of the study protocol), for which a close site monitoring was maintained in order to consider serious adverse events (SAE) and adverse events of special interest (AESI). The interim safety analysis was performed in October 2020 after the first six patients have been treated for three cycles. The results from the interim safety analysis and all available data (also from other clinical trials) regarding the drugs used in this trial were reviewed by the GPI, the coordinating PI, one statistician and other members of the protocol committee. It was decided to continue the recruitment of the study without any additional safety precautions.

The scope of this document is restricted to the primary endpoint analysis and does not include the interim safety analysis.

3 Study design

3.1 Study design

The study is a phase-II open label trial, which is designed as a prospective, multicentre, single-arm trial. Patients with previously untreated Richter Transformation (RT) or patients who responded to up to one prior line of RT therapy were included. The study aims to investigate the efficacy and safety of a regimen consisting of 6 cycles of tislelizumab plus zanubrutinib as induction therapy, followed by 6 cycles of tislelizumab plus zanubrutinib as consolidation therapy (each cycle with 21 days, 200 mg tislelizumab at day 1 of each cycle, 160 mg zanubrutinib twice daily within each cycle). Patients with complete response (CR), partial response (PR) or stable disease to therapy continue treatment until disease progression or non-tolerance. Patients with prior response and progression during treatment interruption may continue with therapy after consulting with the sponsor`s representative, GCLLSG study office. Patients with response to treatment can undergo stem cell transplantation (SCT) any time after induction therapy.

3.2 Study objectives

The primary objective of the study is to evaluate the efficacy of a combinational therapy with tislelizumab and zanubrutinib in CLL patients with RT to diffuse large B cell lymphoma (DLBCL) or Hodgkin’s lymphoma (HL).

The secondary objective is to evaluate the safety of a combinational therapy with tislelizumab and zanubrutinib in CLL patients with RT to DLBCL or HL.

3.3 Study endpoints

3.3.1 Primary endpoint

Overall response rate (ORR) after induction therapy (i.e. 6 cycles) according to the refined Lugano Classification (Cheson et al, 2016):

- CR
- PR

3.3.2 Secondary endpoints

- ORR after induction therapy (i.e. 6 cycles) according to IWCLL criteria (Hallek et al, 2018)
- ORR after consolidation therapy (i.e. 12 cycles)
- Duration of response (DOR)
- Progression-free survival (PFS)
- Overall survival (OS)
- Time to next treatment (TTNT)
- Proportion of patients receiving SCT for consolidation
- Safety parameters: type, frequency, severity of adverse events (AEs), and their relationship to study treatment

3.3.3 Exploratory endpoints

- Evaluation of relationship between various baseline markers, including PD-1/PD-L1 expression and mutational load, and clinical outcome parameters. In particular:
    - ORR after 6 cycles, PFS, and OS for the RT naïve vs. previously RT treated population
    - ORR after 6 cycles and PFS for the BTKi naïve vs. the previously BTKi treated population
    - Univariate analyses of potential prognostic factors for ORR/PFS/OS/DOR
- Modified time to next treatment (modified TTNT, by defining death as an event)
- Clinical outcome parameters for the intention to treat (ITT) population

3.4 Study assumptions and sample size calculation

The primary endpoint ORR at end of induction therapy was used to determine the sample size of the study. The following study assumptions are considered:

- The ORR for a conventional regimen is assumed to be 40% with corresponding null hypothesis ORR ≤ 0.40 and alternative hypothesis ORR > 0.40.
- The investigated regimen is considered potentially useful and worthy of further research if we can reject the null hypothesis in favour of the alternative hypothesis.
- The type I error is set to 2.5% and defines the chance that the investigated regimen will be investigated further although the true ORR is lower or equal to 40%.
- The type II error is the chance that an effective treatment will not be studied further. It is assumed to improve the ORR to at least 60% with the investigated regimen. The type II error should not exceed 20%, so that it is aimed to achieve a power of at least 80% at the assumed ORR.

According to the above determined study parameters a one-sided single-sample binomial test with an overall significance level of 2.5% provides the sample size N = 48, such that statistical significance is achieved with a power of 80%.

4 Study populations

4.1 Inclusion criteria

- Confirmed diagnosis of CLL according to iwCLL criteria (Hallek et al, 2018).
- Confirmed histopathological diagnosis of RT (diffuse large B-cell lymphoma or Hodgkin’s lymphoma)
- Previously untreated RT or patients with objective response or non-tolerance to first-line RT treatment
- Creatinine clearance ≥30ml/min calculated according to the modified formula of Cockcroft and Gault or directly measured with 24hr urine collection or an equivalent method.
- Adequate liver function as indicated by a total bilirubin ≤ 2, AST/ALT ≤ 2.5 the institutional ULN value, unless directly attributable to the patient’s CLL or to Gilbert’s Syndrome, in which case a max. total bilirubin ≤ 4 and AST/ALT ≤ 5 the institutional ULN value are required.
- Negative serological testing for hepatitis B (HBsAg negative and anti-HBc negative; patients positive for anti-HBc may be included if PCR for HBV DNA is negative and HBV-DNA PCR is performed every two months until 2 months after last dose of zanubrutinib), negative testing for hepatitis-C RNA and negative HIV test within 6 weeks prior to registration
- Age at least 18 years
- ECOG performance status 0-2, ECOG 3 is only permitted if related to CLL (e.g. due to anaemia or severe constitutional symptoms)
- Life expectancy ≥ 3 months
- Ability and willingness to provide written informed consent and to adhere to the study visit schedule and other protocol requirements

4.2 Exclusion criteria

- Patients who did not respond to previous line of RT therapy (i.e. primary progressive patients)
- Patients with more than one prior line of RT therapy
- Allogenic stem cell transplantation within the last 100 days or signs of active GVHD after prior allogeneic stem cell transplantation within any time
- Patients with confirmed PML
- Uncontrolled autoimmune condition
- Malignancies other than CLL currently requiring systemic therapies (unless the malignant disease is in a stable remission at the discretion of the treating physician)
- Active infection currently requiring systemic treatment
- Any comorbidity or organ system impairment rated with a CIRS (cumulative illness rating scale) score of 4, excluding the eyes/ears/nose/throat/larynx organ system 1 or any other

life-threatening illness, medical condition or organ system dysfunction that – in the investigator´s opinion could comprise the patient's safety or interfere with the absorption or metabolism of the study drugs

- Requirement of therapy with strong CYP3A4 inhibitors/inducers
- Requirement of therapy with phenprocoumon or other vitamin K antagonists.
- Use of investigational agents, e.g. monoclonal antibodies or other experimental drugs within clinical trials, which might interfere with the study drug within 28 days (or 5 times half-life of the compound, whichever is longer) prior to registration
- Known hypersensitivity to tislelizumab, zanubrutinib or any of the excipients
- Pregnant women and nursing mothers (a negative pregnancy test is required for all women of childbearing potential within 7 days before start of treatment)
- Fertile men or women of childbearing potential unless:
    - surgically sterile or ≥ 2 years after the onset of menopause, or
    - willing to use two methods of reliable contraception including one highly effective contraceptive method (Pearl Index < 1) and one additional effective (barrier) method during study treatment and for 12 months after the end of study treatment.
- Vaccination with a live vaccine < 28 days prior to randomization
- Legal incapacity
- Prisoners or subjects who are institutionalized by regulatory or court order
- Persons who are in dependence to the sponsor or an investigator

4.3 Full analysis set

The full analysis set (FAS) comprises of all enrolled patients who received at least two complete cycles of induction therapy (efficacy population). This means that at least one dose of any compound of the trial medication has to be documented for the third cycle of induction treatment. The FAS is the target population for the primary and secondary efficacy endpoints and shall be used for analysis of all study endpoints except safety. Patients with early discontinuation from study treatment (i.e. discontinuation prior to administration of third induction cycle) will be reported separately from the FAS.

4.4 Safety population

The safety population (SP) is defined as all subjects enrolled in the study receiving at least one dose of trial treatment, whether withdrawn prematurely or not. The safety population shall be used for evaluating the safety endpoints.

4.5 Intention to treat population

The intention to treat (ITT) population comprises of all enrolled patients. The ITT population for efficacy endpoints shall be used for exploratory analyses.

5 Analysis time-point

The primary endpoint can be analysed as soon as all enrolled patients who did not discontinue prematurely have completed the interim staging after 6 cycles of induction therapy. This will trigger the time point of the analysis of the primary and secondary endpoints. Exploratory analyses may be performed after or at the time point of primary and secondary efficacy analyses.

6 Study population and study endpoints evaluated in primary endpoint analysis

In total, 59 patients have been enrolled at the end of recruitment constituting the CLL-RT1 ITT population. One patient died before receiving the first dose of trial medication and one patient withdrew the consent, thus leaving 57 patients in the safety population. A further 9 patients discontinued study treatment early and will not be allocated to the FAS. Thus, the FAS will comprise of 48 patients.

The following selected secondary endpoints will be evaluated in the primary endpoint analysis:

- ORR after induction therapy according to IWCLL criteria[1]
- ORR after consolidation therapy
- PFS
- DOR
- OS
- TTNT

Furthermore, safety evaluations (including type, frequency, and severity of AEs) will be performed; treatment exposure as well as basic patient characteristics and observation time will be described. Other secondary endpoints will be evaluated at a later time point with more mature data based on longer observation time.

The following exploratory analyses will be evaluated:

- Modified TTNT (including the event: death due to any cause)
- Clinical outcome parameters (ORR after 6 cycles, PFS, OS, TTNT, modified TTNT) for the ITT population
- ORR after 6 cycles, PFS, and OS for the RT naïve vs. previously RT treated population
- ORR after 6 cycles and PFS for the BTKi naïve vs. the previously BTKi treated population
- Univariate analyses of potential prognostic factors for ORR/PFS/OS/DOR

7 Definition of endpoints

The following section describes the definitions of rate-based and time-to-event endpoints considered for the primary endpoint analysis.

[1] Differences between the definition of ORR according to iwCLL and Lugano will be assessed prior to analyses. If no discrepancies occur between the two definitions, ORR after induction therapy according to iwCLL coincides with the primary endpoint.

7.1 Definition of rate-based endpoints

7.1.1 Overall response rate (ORR) after induction therapy according to Lugano Classification

The primary efficacy variable (primary endpoint) is the overall response rate (ORR) at interim staging after end of induction therapy (end of induction treatment response) according to Lugano Classification. ORR is defined as the proportion of patients having achieved a CR or PR (according to the refined Lugano Classification). Patients without any documented response assessment will be kept and labelled as 'non-responder' in the analysis.

7.1.2 Overall response rate (ORR) after induction therapy according to IWCLL criteria

The ORR at interim staging after end of induction therapy (end of induction treatment response) according to IWCLL criteria is defined as the proportion of patients having achieved a CR, CRi or PR (according to IWCLL criteria). Patients without any documented response assessment will be kept and labelled as 'non-responder' in the analysis.

7.2 Definition of time-to-event endpoints

7.2.1 Progression-free survival (PFS)

Progression-free survival (PFS) will be measured from the date of registration to the date of first occurrence of disease progression or relapse or death from any cause, whichever occurs first. These will be counted as events for PFS. Start of a subsequent CLL/RT treatment after the study treatment will not be counted as an event nor as a reason for censoring. Patients for whom no documented event for PFS is available at the time of analysis will be censored at the time point of last observation they were assessed to be event-free. The date of event or censoring will be determined according to the scheme described in Table 1.

Table 1 | Scheme for PFS calculation

Scenario	PFS status	Date of event or censoring	Source file for the determination of date **VARIABLES** [data set]
PD is recorded	Event	Date of first documented PD	<u>Response assessment</u><br>*"Date of response assessment"* with<br>• **PDDT** [pd]
Death	Event	Date of death	<u>Death page</u> *"Date of death"*<br>• **DTHDT** [dth]
Alive without PD record	Censored	Date of last response/tumour assessment	Check availability of *"Date of first documented PD"* and *"Date of death"*<br><br>Latest date of<br><u>Response assessment</u> *"Date of response assessment"*<br>• **RESPDT** [resp]<br><br><u>Visit information</u> (Follow-up) *"Visit date"*<br>• **VISDT** [visit]

Alive without PD record or staging/ follow-up visit after registration	Censored	Date of registration + 1 day	Check availability of *"Date of first documented PD"*, *"Date of death", and "Date of last response/tumour assessment".*<br><br>Medical review enrollment confirmation<br>*"Date of enrollment"* + 1 day<br>• **MRENRDT** [mrscr]

7.2.2 Duration of response (DOR)

DOR will be calculated for patients with CR or PR after induction therapy according to Lugano Classification (according to the definition of ORR after induction therapy according to Lugano Classification). DOR will be measured from the date of first documented response to the first occurrence of progression or relapse (according to Lugano Classification) or death by any cause, whichever occurs first. These will be counted as events for DOR. Patients for whom no documented event for DOR is available at the time of analysis will be censored on the date of the last tumor assessment. If no tumor assessments were performed after the screening visit, DOR will be censored at the time of first documented response + 1 day. The date of event or censoring will be determined according to the definition of PFS as described in Table 1.

7.2.3 Overall survival (OS)

Overall survival (OS) will be measured from the date of registration to the date of death due to any cause. Patients who have not yet died at the time of analysis will be censored at the time of last observation they were assessed to be alive after registration as described in Table 2.

Table 2 | Calculation of the date of last observation

Date of last observation	Source file for the determination of date<br>**VARIABLES** [data set]
Date of last recorded information that patient is alive	Medical review enrollment confirmation *"Date of enrollment"* + 1 day<br>• **MRENRDT** [mrscr]<br><br>Adverse event *"Date of onset", "Resolution date (AE, SAE)", "Date of discontinuation"*, *"Date of last dose"*, and *"Start of treatment"*<br>• **AESTD**, **AEENDT**, and **AESENDT** [ae]<br>• **AESDIDT**, **AESLDDT**, and **AESFCDT** [aesdr]<br><br>Bone marrow *"Date of assessment"*<br>• **BMDT** [bm]<br><br>Concomitant medications *"Start date"* and *"End date"*<br>• **CMSTDT** and **CMENDT** [cm]<br><br>End of study report *"Date of end of study"*<br>• **DSSTDT** [ds]<br><br>End of treatment *"Date of discontinuation"*<br>• **EOTDT** [eot]<br><br>Tislelizumab infusion *"Date of infusion"*

	• **EXTSTDT** [ext]<br><br><u>Zanubrutinib infusion</u> *“Zanubrutinib administration start date” and “End date”*<br>• **EXZSTDT** and **EXZENDT** [exz]<br><br><u>Lab results</u> (chemistry and hematology) *“Date/Time of Specimen Collection”*<br>• **LBDT** [lb]<br><br><u>Physical examination</u> *“Date of assessment”*<br>• **PEDT** [pe]<br><br><u>Pregnancy test</u> *“Date of pregnancy test”*<br>• **PGDT** [pg]<br><br><u>Radiology assessment</u> *“Date of Ultrasound/PET-CT/MRI scan”*<br>• **VISITDT** [ract, rapet, raus]<br><br><u>Response assessment</u> *“Date of response assessment”*<br>• **RESPDT** [resp]<br><br><u>(Re-)assessment of Richter's syndrome</u><br>*“Date of lymph/bone marrow/other assessment”*<br>• **PT2LYDT**, **PT2BODT**, and **PT2OTDT** [rsd]<br><br><u>Therapies</u> *“Start/end dates of new CLL-treatment/drugs of combination/regime”*, *“Date of response assessment”*, and *“Date of progression”*<br>• **TPDRSTDT**, **TPDRENDT**, **TPTRSTDT**, **TPTRENDT**, **TPRESPDT**, and **TPREPDDT** [tp]<br><br><u>Transplantation</u> *“Date of transplantation”, “Date of first DLI”, “Date of last DLI”, and Date of progression*<br>• **TXPDT**, **TXPFDLDT**, **TXPLDLDT, and TXPRPDDT** [txp]<br><br><u>Visit information</u> (Follow-up) *“Visit date”*<br>• **VISDT** [visit]

7.2.4 Time to next CLL treatment (TTNT)

Time to next treatment (TTNT) will be measured from date of registration to the date of first subsequent CLL/RT treatment (including transplantation). These will be counted as event for TTNT. Alive patients for whom no subsequent CLL/RT treatment is documented will be censored at the time of last observation they were assessed to be alive. Deceased patients for whom no subsequent CLL/RT treatment is documented will be censored at the date of death. The date of event or censoring will be determined according to the scheme described in Table 3.

Table 3 | Scheme of TTNT calculation

Scenario	TTNT status	Date of event or censoring	Source file for the determination of date **VARIABLES** [data set]
	Event	Date of start of first subsequent	Earliest date of<br><u>Therapies</u> *“Start date of new CLL/RT treatment”*

Initiation of subsequent treatment for CLL		treatment/ transplantation	• **TPTRSTDT**, [tp] after **MRENRDT** [mrscr] *"Date of enrollment"*<br><br>Transplantation *"Start date of transplantation"*<br>• **TXPDT** [txp] after **MRENRDT** [mrscr] *"Date of enrollment"*
Death	Censored	Date of death	Death page *"Date of death"*<br>• **DTHDT** [dth]
Alive without initiation of subsequent treatment documented	Censored	Date of last recorded information that patient is alive	Check availability of *"Date of start of first subsequent treatment/transplantation"* and *"Date of death"* Then, *"Date of last recorded information that patient is alive"* (cf. Table 1)
Alive without initiation of subsequent treatment documented and no assessment records after registration	Censored	Date of enrollment + 1 day	Check availability of *"Date of start of first subsequent treatment/transplantation"* and *"Date of death"* and *"Date of last recorded information that patient is alive"*<br>Then,<br>Medical review enrollment confirmation<br>*"Date of enrollment"* + 1 day<br>• **MRENRDT** [mrscr]

7.2.5 Modified time to next CLL treatment (modified TTNT)

Modified time to next treatment (modified TTNT) will be measured from date of registration to the date of first subsequent CLL/RT treatment (including transplantation) or death from any cause, whichever occurs first. These will be counted as event for modified TTNT. Alive patients for whom no subsequent CLL/RT treatment is documented will be censored at the time of last observation they were assessed to be alive.

8 Statistical analyses

8.1 Description of basic information

8.1.1 Description of study populations

The allocation to the different populations (ITT, FAS, SP) will be described and the number of patients constituting the ITT, FAS, and the SP will be reported as well as the reasons for exclusions of patients from a certain population. In terms of the trial profile, a CONSORT diagram according to ATT 01 ("Template CONSORT diagram") will be prepared.

8.1.2 Observation time

To assess the observation time of the study the reverse Kaplan-Meier survival methodology will be applied to the continuous overall survival variable referring to the difference between registration and death or last information the patient was known to be alive in months (according

to the definition of overall survival). Median observation time (including interquartile range, minimum, and maximum) will be estimated with respect to the FAS.

8.1.3 Demographic and baseline characteristics

Demographic and baseline characteristics at screening will be presented for both the ITT population and the FAS. The following parameters will be reported for these populations:

- RT stratum [previously untreated RT | previously treated with RT-directed therapy][2] [DLBCL (including cell of origin: non-GCB versus GCB) | HL][3] [clonally unrelated | clonally related]
  - List of previous RT-directed therapies
- CLL stratum [previously untreated CLL | previously treated with CLL-directed therapy][4]
  - Number of previous CLL-directed treatments [restricted patients to previously treated with CLL-directed therapy; descriptive statistics] including
    - List of previous CLL-directed therapies
    - Number of patients previously treated with chemo(immuno)therapy[5]
    - Number of patients previously treated with BTK/BCL2 inhibitors[6]
- Age [years] [descriptive statistics: including cut-off value: ≤ 65 | > 65]
- Gender [male | female]
- Time between first diagnosis and registration [months] [descriptive statistics]
- Binet stage [A | B | C]
- Severe constitutional symptoms [no | yes]
- ECOG performance status [0 | 1 | 2 | 3 | 4] [cut-off value: > 0]
- Cumulative illness rating scale (CIRS) [total CIRS score] [descriptive statistics; including cut-off values: > 1; > 6]
- Creatinine clearance [mL/min] (according to the formula of Cockcroft-Gault with the parameters serum creatinine [mg/dl], gender, age [years] and weight [kg]) [descriptive statistics; including cut-off values: ≥ 30 ml/min; ≥ 70 ml/min]
- LDH [U/L] [descriptive statistics]
- Cytogenetic subgroups (by FISH) by hierarchical order (according to Döhner et al. NEJM 2000)
  - Deletion 17p
  - Deletion 11q
  - Trisomy 12
  - No abnormalities
  - Deletion 13q
- *TP53* mutational status [mutated | unmutated]
- *TP53* status [deleted and/or mutated | none]
- IGHV mutational status [mutated | unmutated | not evaluable]
- Serum thymidine kinase [U/L] [descriptive statistics: including cut-off value: > 10.0 U/L]

---

[2] Variable TPINDC.
[3] Determined by CP and provided as excel sheet „RT1_histo_coo_ki67“, variable „Histology“.
[4] Variable TPINDC.
[5] Variable TPCAT.
[6] Variable TPCAT.

- Serum β2-microglobulin [mg/L] [descriptive statistics: including cut-off value : > 3.5]
- Complex karyotype [< 3 aberrations \ ≥ 3aberrations]
- CLL-IPI Risk Group [low │ intermediate │ high │ very high]
- Ki-67 % [descriptive statistics]

Descriptive statistics for continuous variables including median, interquartile range, mean, minimum, maximum and standard deviation will be used. Additionally, selected continuous variables will be dichotomized according pre-defined cut-off values. Categorical variables will be reported with relative frequencies. Moreover, demographic and baseline characteristics will be reported regarding to the subgroup of patients for whom a respective parameter is available. Thereto the number of patients available and the proportion of patients for whom data are missing will be described with respect to the FAS population and the ITT.

8.2 Primary efficacy analysis

The primary efficacy analysis will be performed with a single stage phase II design with respect to the FAS. The ORR of the study treatment will be compared with the benchmark of 40% using a one-sided one-sample binomial test. Statistical significance will be defined as $p < 0.025$. The efficacy of the study treatment will be concluded if the null hypothesis is rejected.
The relative frequency of patients having responded to therapy at interim staging after induction therapy including the corresponding 95% Clopper Pearson confidence interval will be reported with respect to the FAS.

8.3 Analyses of secondary endpoints

Analyses of secondary efficacy endpoints will be not tested formally and there is no control of type I error of any study endpoint. Every endpoint will be descriptively analysed and reported. Analyses will be based on the FAS.

Rate based endpoints shall be assessed showing frequencies and corresponding percentages including 95% Clopper Pearson confidence intervals.

Analyses of time-to-event endpoints will be performed using Kaplan-Meier methods with the Kaplan-Meier survival curve presented to provide a visual description. Kaplan-Meier estimates of median time and rates for 6, 12 and 24 months after registration will be reported. With regard to OS and TTNT, the exact cause of death as well as all documented subsequent treatments (particularly the proportion of patients having received SCT) will be reported additionally.

8.4 Safety analysis

Analysis of safety parameters will be performed on the SP unless stated otherwise.

AEs will be classified using the Medical Dictionary for Regulatory Activities (MedDRA) classification system. AEs will be reported by MedDRA system organ class (SOC), high level term (HLT), high level group term (HLGT), and preferred term (PT). The severity of the AEs will be graded according to the recent updated NCI CTCAE version 5.

First, all cases of AEs will be reported in a case analysis referring to the following time frames: *Whole study conduct* and *induction phase*. The case analyses will include reported SOC terms,

severity (CTC grade 1-5), number of serious events, time point of occurrence, outcome, adjustment of study drugs including type of adjustment and number of events related to study drugs (according to the investigator). These parameters will be summarized by counts and percentages. Additionally, tables including all AEs with unknown CTC Grade, CTC Grade 5 as well as all AEs leading to treatment discontinuation will be prepared. PTs will be listed for every reported SOC category in induction phase.

Second, AEs will be reported in a by-patient analysis referring to the time frames of *whole study conduct* and *induction phase*. By-patient analysis means, that an event will be counted once only (with worst NCI CTCAE) if a subject has the same event more than once. Frequency tables including the number and proportion of patients with at least one AE classified as maximum CTC grade 1-5, maximum CTC grade 3-5, and maximum CTC grades 1-2, 3, 4, and 5 will be prepared per SOC and common PT (with frequency of occurrence of at least 5). Additionally, a frequency table including the number and proportion of patients with at least one AE leading to treatment discontinuation will be prepared.

8.5 Treatment exposure

- Number of cycles administered with at least one dose of study treatment [per patient; based on FAS and additionally based on patients excluded from the FAS]

- Number of patients still on treatment and number of patients with treatment discontinuations [based on FAS and additionally based on patients excluded from the FAS]
    - Number of cycles administered with at least one dose of study treatment [per patient; based on patients of FAS still on treatment]
    - Number of cycles administered with at least one dose of study treatment [per patient; based on patients of FAS with treatment discontinuation]
    - Description of reasons for discontinuation according to “End of Treatment”- page including the following hierarchical order:
        - Progressive disease
        - Death
        - Adverse event
        - Patient withdrew consent
        - Patient refused treatment / did not cooperate
        - Other / pregnancy / new RT/CLL treatment (including specification)
    - Number of patients with treatment discontinuation due to SCT as new RT/CLL treatment

- **Tislelizumab exposure during induction phase [per patient; based on the FAS]**
    - Total cumulative dose and dose intensity (fraction of total planned dose [=1,200 mg] and total cumulative dose) [descriptive statistics]
    - Number of cycles with dose > 0 mg
    - Number of cycles with planned dose = 200 mg
    - Number of patients with at least one dose modification

- **Zanubrutinib exposure during induction phase [per patient; based on the FAS]**
    - Days with dose = 0mg [descriptive statistics]

- Days with any dose > 0mg [descriptive statistics]
- Days with planned dose = 160mg [descriptive statistics]
- Total cumulative dose and dose intensity (fraction of total planned dose [= 40,320 mg] and total cumulative dose) [descriptive statistics]
- Number of cycles with dose > 0 mg
- Number of patients with at least one dose modification
- Number of patients with at least one dose modification due to adverse events

Continuous variables (i.e. days with dose = 0mg, days with any dose > 0mg, days with planned dose, total cumulative dose, dose intensity) will be summarized by descriptive statistics including median, IQR, mean, minimum, maximum and standard deviation will be used. Categorical variables will be summarized by counts and percentages.

8.6 Exploratory analyses

As exploratory analyses the modified TTNT as well as clinical outcome parameters (ORR after 6 cycles, PFS) for the ITT population will be evaluated. Moreover, exploratory analyses for the correlations between the primary efficacy parameter and selected secondary efficacy endpoints (PFS, OS, TTNT, and modified TTNT) and several clinical parameters (previously untreated RT/BTKi versus previously treated with RT/BTKi-directed therapy and DLBCL versus HL) will be performed without any formal statistical testing. The 95% CIs for the primary endpoint and secondary or exploratory response endpoints were calculated according to the Clopper-Pearson method, and the Kaplan-Meier method was used for the time-to-event analyses of the secondary or exploratory endpoints. Univariate analyses of potential prognostic factors were performed for ORR after 6 cycles using logistic regression modelling and for OS, PFS, and DOR using Cox proportional hazards regression modelling, each without adjustment for multiple testing.

For patients with treatment discontinuation due to SCT as new RT/CLL treatment PFS and OS will be additionally calculated from time point of SCT.

Data display for rate-based endpoints will contain cross tabulations including counts and percentages. Analyses of time-to-event endpoints will be performed using Kaplan-Meier methods with the Kaplan-Meier survival curves presented to provide a visual description. Kaplan-Meier estimates of median time and rates for 6, 12 and 24 months after registration will be reported for each subgroup.

9 Changes to the analyses specified in the protocol

According to the current version of the protocol, PFS should be calculated in two different ways:

1) Until the date of first occurrence of disease progression or relapse (according to Lugano Classification) or death.
2) Until the date of first occurrence of disease progression or relapse (according to iwCLL criteria) or death.

As the date of progression according to Lugano Classification contained many missing values due to the fact that the imaging examination has not been performed, it was decided to retrieve the progression dates and events only from the PD page (data set [pd]), that encompasses the progression dates and events according to iwCLL criteria and Lugano Classification (from the [resp] data set). Thus, PFS will be analysed without differentiation between progressions by Lugano Classification and iwCLL criteria.

The study protocol will be amended accordingly.

10 Handling of missing data

Missing data will not be replaced or imputed. For all analyses the number of patients available and the proportion of patients for whom data are missing will be described with respect to the FAS or SP, respectively.

11 Preparation of datasets

Clinical data will be cleaned according to study specific data management, medical review and safety management plans in advance to the data export for the primary endpoint analysis.

The data export from the clinical database will be prepared and structured according to the database interface document (Y:\Studien\CLL-RT1\1. DE\18. Datenmanagement\18.3 Datenbank\18.3.1. Entwicklung_Validierung\Export Anforderung Statistik). Based on the raw data files, different analysis datasets will be prepared according to the dataset descriptions specified in the attachment ATT 02 (“*Descriptions of prepared datasets for statistical analyses*”). The steps of the programming will be documented in separate SPSS syntax files.

A separate data export will be planned from the central labs for the cytogenetic baseline parameters (Ulm), the serum parameters β2-microglobulin and thymidine kinase (Cologne), and the karyotyping data (Cologne).
