# Supplementary material for: Tislelizumab plus zanubrutinib for Richter transformation: the phase 2 RT1 trial
Source: Nat Med. 2023 Dec 9;30(1):240–8. doi: 10.1038/s41591-023-02722-9 (PMC10803258; doi:10.1038/s41591-023-02722-9)
Supplement: Supplementary file 2 — Reporting Summary [file 41591_2023_2722_MOESM2_ESM.pdf]

nature portfolio

Corresponding author(s): Othman Al-Sawaf
Barbara Eichhorst

Last updated by author(s): Oct 19, 2023

Reporting Summary

Nature Portfolio wishes to improve the reproducibility of the work that we publish. This form provides structure for consistency and transparency in reporting. For further information on Nature Portfolio policies, see our Editorial Policies and the Editorial Policy Checklist.

Statistics

For all statistical analyses, confirm that the following items are present in the figure legend, table legend, main text, or Methods section.

n/a	Confirmed	
☐	☒	The exact sample size (*n*) for each experimental group/condition, given as a discrete number and unit of measurement
☐	☒	A statement on whether measurements were taken from distinct samples or whether the same sample was measured repeatedly
☐	☒	The statistical test(s) used AND whether they are one- or two-sided<br>*Only common tests should be described solely by name; describe more complex techniques in the Methods section.*
☐	☒	A description of all covariates tested
☐	☒	A description of any assumptions or corrections, such as tests of normality and adjustment for multiple comparisons
☐	☒	A full description of the statistical parameters including central tendency (e.g. means) or other basic estimates (e.g. regression coefficient) AND variation (e.g. standard deviation) or associated estimates of uncertainty (e.g. confidence intervals)
☐	☒	For null hypothesis testing, the test statistic (e.g. *F*, *t*, *r*) with confidence intervals, effect sizes, degrees of freedom and *P* value noted<br>*Give P values as exact values whenever suitable.*
☒	☐	For Bayesian analysis, information on the choice of priors and Markov chain Monte Carlo settings
☒	☐	For hierarchical and complex designs, identification of the appropriate level for tests and full reporting of outcomes
☒	☐	Estimates of effect sizes (e.g. Cohen's *d*, Pearson's *r*), indicating how they were calculated

Our web collection on statistics for biologists contains articles on many of the points above.

Software and code

Policy information about availability of computer code

Data collection	The TrialMaster version 5 system from OmniComm (OmniComm Europe GmbH, An Anju Software Company) is used as the study database. Technical support as well as software development and maintenance are provided by the provider OmniComm.
Data analysis	Sample size calculations were performed with EAST 6 software and validated with Binomial tables. Analyses were performed using IBM SPSS Statistics version 29.0 and R version 4.2.1.

For manuscripts utilizing custom algorithms or software that are central to the research but not yet described in published literature, software must be made available to editors and reviewers. We strongly encourage code deposition in a community repository (e.g. GitHub). See the Nature Portfolio guidelines for submitting code & software for further information.

Data

Policy information about availability of data

All manuscripts must include a data availability statement. This statement should provide the following information, where applicable:

- Accession codes, unique identifiers, or web links for publicly available datasets
- A description of any restrictions on data availability
- For clinical datasets or third party data, please ensure that the statement adheres to our policy

Access to individual patient-level data can be requested after publication via the corresponding authors (othman.al-sawaf@uk-koeln.de, barbara.eichhorst@uk-

koeln.de), who will facilitate a central review by the GCLLSG within 6 months. The data will be released to such requesters with necessary agreements to enforce terms such as security, patient privacy, and consent of specified data use, consistent with evolving, applicable data protection laws.

Research involving human participants, their data, or biological material

Policy information about studies with human participants or human data. See also policy information about sex, gender (identity/presentation), and sexual orientation and race, ethnicity and racism.

Reporting on sex and gender	Sex and gender were not considered in the study design. Self-reported sex was collected and used in the analyses. The ratio of male to female patients enrolled in the study was ~1.5:1, in line with the general incidence of CLL and RT, which is slightly more common in male persons than female persons. Given the sample size, a post hoc sex-based analysis would not yield reliable or representative findings. However, sex was included as a variable in univariate tests.
Reporting on race, ethnicity, or other socially relevant groupings	No information on race or ethnicity were collected on the CRFs.
Population characteristics	Based on the inclusion and exclusion criteria (see below), patients were recruited from 10 sites in Germany, 1 site in Austria plus 1 site in Denmark. Between 11 February 2020 and 2 January 2023.<br>Inclusion criteria:<br>• Confirmed diagnosis of CLL according to iwCLL criteria (Hallek et al, 2018).<br>• Confirmed histopathological diagnosis of RT (diffuse large B-cell lymphoma or Hodgkin's lymphoma)<br>• Previously untreated RT or patients with objective response or non-tolerance to first-line RT treatment<br>• Creatinine clearance ≥30ml/min calculated according to the modified formula of Cockcroft and Gault or directly measured with 24hr urine collection or an equivalent method.<br>• Adequate liver function as indicated by a total bilirubin ≤ 2, AST/ALT ≤ 2.5 the institutional ULN value, unless directly attributable to the patient's CLL or to Gilbert's Syndrome, in which case a max. total bilirubin ≤ 4 and AST/ALT ≤ 5 the institutional ULN value are required.<br>• Negative serological testing for hepatitis B (HBsAg negative and anti-HBc negative; patients positive for anti-HBc may be included if PCR for HBV DNA is negative and HBV-DNA PCR is performed every two months until 2 months after last dose of zanubrutinib), negative testing for hepatitis-C RNA and negative HIV test within 6 weeks prior to registration<br>• Age at least 18 years<br>• ECOG performance status 0-2, ECOG 3 is only permitted if related to CLL (e.g. due to anaemia or severe constitutional symptoms)<br>• Life expectancy ≥ 3 months<br>• Ability and willingness to provide written informed consent and to adhere to the study visit schedule and other protocol requirements<br>Exclusion criteria:<br>• Patients who did not respond to previous line of RT therapy (i.e. primary progressive patients)<br>• Patients with more than one prior line of RT therapy<br>• Allogenic stem cell transplantation within the last 100 days or signs of active GVHD after prior allogeneic stem cell transplantation within any time<br>• Patients with confirmed PML<br>• Uncontrolled autoimmune condition<br>• Malignancies other than CLL currently requiring systemic therapies (unless the malignant disease is in a stable remission at the discretion of the treating physician)<br>• Active infection currently requiring systemic treatment<br>• Any comorbidity or organ system impairment rated with a CIRS (cumulative illness rating scale) score of 4, excluding the eyes/ears/nose/throat/larynx organ system 1 or any other life-threatening illness, medical condition or organ system dysfunction that – in the investigator´s opinion could comprise the patient's safety or interfere with the absorption or metabolism of the study drugs<br>• Requirement of therapy with strong CYP3A4 inhibitors/inducers<br>• Requirement of therapy with phenprocoumon or other vitamin K antagonists.<br>• Use of investigational agents, e.g. monoclonal antibodies or other experimental drugs within clinical trials, which might interfere with the study drug within 28 days (or 5 times half-life of the compound, whichever is longer) prior to registration<br>• Known hypersensitivity to tislelizumab, zanubrutinib or any of the excipients<br>• Pregnant women and nursing mothers (a negative pregnancy test is required for all women of childbearing potential within 7 days before start of treatment)<br>• Fertile men or women of childbearing potential unless:<br>- surgically sterile or ≥ 2 years after the onset of menopause, or<br>- willing to use two methods of reliable contraception including one highly effective contraceptive method (Pearl Index < 1) and one additional effective (barrier) method during study treatment and for 12 months after the end of study treatment.<br>• Vaccination with a live vaccine < 28 days prior to randomization<br>• Legal incapacity<br>• Prisoners or subjects who are institutionalized by regulatory or court order<br>• Persons who are in dependence to the sponsor or an investigator<br><br>Covariate-relevant patient characteristics: Age (range 45-82 years), self-reported sex, Binet stage (A/B/C), severe constitutional symptoms (absent/present), ECOG performance status (range 0-3), Cumulative illness rating scale (CIRS; IQR 0-7), LDH (U/L; IQR 209-584), Cytogenetic subgroups (deletion 17p, 11q, trisomy 12, none, deletion 13q), TP53 and IGHV mutation status (unmutated/mutated), Serum thymidine kinase (U/L; IQR 18.5-108.3), Serum β2-microglobulin (mg/L; IQR 2.5-5.5), Complex karyotype (≥ 3 aberrations), and CLL-IPI Risk Group (Low/Intermediate/High/Very high).
Recruitment	Based on the inclusion and exclusion criteria (see below), patients were recruited from 10 sites in Germany, 1 site in Austria

Recruitment	plus 1 site in Denmark. Between 11 February 2020 and 2 January 2023. Patients were considered from routine clinical care based on a diagnosis of Richter-Transformation. As selection bias towards patients without primary progressive disease cannot be excluded, as these patients were excluded from the study based on the protocol-based exclusion criteria. The study therefore focuses on patients with previously untreated RT or up to one prior line of effective RT-directed therapy.
Ethics oversight	The Sponsor (University of Cologne) and all investigators agree to conduct this study in accordance with the protocol and with consensus ethical principles derived from international guidelines including the Declaration of Helsinki and Council for International Organizations of Medical Sciences (CIOMS) International Ethical Guidelines, applicable ICH Good Clinical Practice (GCP) Guidelines and applicable laws and regulations. The participating investigators/institutions permit trial-related monitoring, audits, IRB/IEC review and regulatory inspections and provide direct access to source documents/data. The investigator assumes the responsibility of obtaining written informed consent for each patient before any study-specific procedures are performed and before any study drug is administered. If an amendment to the protocol changes the risk participation schedule in scope or activity, or increases the potential risk to the subject, the informed consent document must be used to obtain re-consent from any patients currently enrolled in the study if the patient is affected by the amendment and must be used to document consent from any new patients enrolled after the approval date of the amendment. The Sponsor and the investigators affirm and uphold the principle of the patients' right to privacy. The investigators shall comply with applicable privacy laws. The investigator must assure that the patients' anonymity will be maintained and that the identities are protected from unauthorized parties. The investigator should maintain documents not for submission to the GCLLSG study office e.g. subjects written informed consent forms, in strict confidence. All clinical and scientific data are collected under a unique code or "pseudonym" composed of an abbreviation of the study and a random string of 6 digits and is stored in the main clinical trial database. All data exchange with the study center is made solely via the unique code. All participating study centers are obliged to keep a secret trial subject identification list.<br>The study was reviewed and approved by all responsible ethics committees (Ethics Committee of the Medical Faculty of the Christian Albrechts University in Kiel; Ethics Committee of the Medical Faculty of the University of Duisburg-Essen; Ethics Committee of the University of Cologne (Central Ethics Committee in Germany); OVGU Ethics Committee at the Medical Faculty; Ethics Committee of the Medical Faculty of the University of Rostock; Ethics Committee of the Medical Faculty of the TU Dresden; Ethics Committee of the University of Ulm; Ethics Committee of the Medical Faculty of the LMU Munich; Ethics committee of the Westfalen-Lippe Medical Association and the Medical Faculty of the Westphalian Wilhelms University of Münster; Ethics Committee of the Bavarian State Medical Association; Ethics Committee of the Medical University of Vienna; National Videnskabsetisk Komité, Copenhagen).

Note that full information on the approval of the study protocol must also be provided in the manuscript.

Field-specific reporting

Please select the one below that is the best fit for your research. If you are not sure, read the appropriate sections before making your selection.

☒ Life sciences ☐ Behavioural & social sciences ☐ Ecological, evolutionary & environmental sciences

For a reference copy of the document with all sections, see nature.com/documents/nr-reporting-summary-flat.pdf

Life sciences study design

All studies must disclose on these points even when the disclosure is negative.

Sample size	The primary endpoint ORR at end of induction therapy was used to determine the sample size of the study. The ORR for a conventional regimen is assumed to be 40%, with corresponding null hypothesis ORR <= 0.40 and alternative hypothesis ORR > 0.40. The investigated regimen was considered potentially useful and worthy of further research if we can reject the null hypothesis in favour of the alternative hypothesis. The type I error is set to 2.5% and defines the chance that the investigated regimen will be investigated further although the true ORR is lower or equal to 40 %. The type II error is the chance that an effective treatment will not be studied further. It is assumed to improve the ORR to at least 60 % with the investigated regimen. The type II error should not exceed 20%, so that it is aimed to achieve a statistical power of at least 80%. According to these study parameters a one-sided one-sample binomial-test with an overall significance level of 2.5% provides the sample size N = 48, such that statistical significance is achieved with a power of 80%.
Data exclusions	Missing data will not be replaced or imputed. For all analyses the number of patients available and the proportion of patients for whom data are missing will be described with respect to the reported analyses populations.
Replication	The study protocol is reported in the CLL-RT1 Protocol version 3.0 (GCLLSG), data handling and statistical analyses are fully documented in the RT1 Data Management Plan and the CLL-RT1 Statistical Analysis Plan. Measurements were taken from distinct samples.
Randomization	The study is a phase-II open label trial, which is designed as a prospective, multicentre, single-arm trial. Randomization is not relevant. Covariates were controlled for by the screening of patients by inclusion and exclusion criteria (above).
Blinding	The study is a phase-II open label trial, which is designed as a prospective, multicentre, single-arm trial. Blinding is not relevant.

Reporting for specific materials, systems and methods

We require information from authors about some types of materials, experimental systems and methods used in many studies. Here, indicate whether each material, system or method listed is relevant to your study. If you are not sure if a list item applies to your research, read the appropriate section before selecting a response.

Materials & experimental systems

n/a	Involved in the study
☒	☐ Antibodies
☒	☐ Eukaryotic cell lines
☒	☐ Palaeontology and archaeology
☒	☐ Animals and other organisms
☐	☒ Clinical data
☒	☐ Dual use research of concern
☒	☐ Plants

Methods

n/a	Involved in the study
☒	☐ ChIP-seq
☒	☐ Flow cytometry
☒	☐ MRI-based neuroimaging

Clinical data

Policy information about clinical studies
All manuscripts should comply with the ICMJE guidelines for publication of clinical research and a completed CONSORT checklist must be included with all submissions.

Clinical trial registration

EudraCT Number: 2018-002492-17, ClinicalTrials.gov Identifier: NCT04271956

Study protocol

Study protocol will be made available in the supplements.

Data collection

Patients were recruited from 10 sites in Germany (University Hospital of Cologne, University Hospital Kiel, University Hospital Essen, Otto-von-Guericke University Magdeburg, University Hospital Rostock, University Hospital Dresden, University Hospital Ulm, Munich Clinic Schwabing, Brüderkrankenhaus Paderborn, MVZ Dr. Vehling-Kaiser GmbH Landshut), 1 site in Austria (Medical University of Vienna) plus 1 site in Denmark (Rigshospitalet Copenhagen). Between 11 February 2020 and 2 January 2023. Data cut-off on May 2nd 2023.

Outcomes

Primary endpoint: Overall response rate (ORR) after induction therapy (i.e. 6 cycles) according to the refined Lugano Classification.
Secondary endpoints:
- ORR after induction therapy (i.e. 6 cycles) according to IWCLL criteria
- ORR after consolidation therapy (i.e. 12 cycles)
- Duration of response (DOR)
- Progression-free survival (PFS)
- Overall survival (OS)
- Time to next treatment (TTNT)
- Proportion of patients receiving SCT for consolidation
- Safety parameters: type, frequency, severity of adverse events, and their relationship to study treatment.

ORR is determined by the proportion of patients with a response after induction therapy.
PFS will be measured from the date of registration to the date of first occurrence of disease progression (PD) or relapse or death from any cause, whichever occurs first. These will be counted as events for PFS. Start of a subsequent CLL/RT treatment after the study treatment will not be counted as an event nor as a reason for censoring. Patients for whom no documented event for PFS is available at the time of analysis will be censored at the time point of last observation they were assessed to be event-free.
DOR will be calculated for patients with response after induction therapy according to Lugano Classification. DOR will be measured from the date of first documented response to the first occurrence of progression or relapse or death by any cause, whichever occurs first. Patients for whom no documented event for DOR is available at the time of analysis will be censored on the date of the last tumor assessment. If no tumor assessments were performed after the screening visit, DOR will be censored at the time of first documented response + 1 day.
OS will be measured from the date of registration to the date of death due to any cause. Patients who have not yet died at the time of analysis will be censored at the time of last observation they were assessed to be alive after registration.
TTNT will be measured from date of registration to the date of first subsequent CLL/RT treatment (including transplantation). These will be counted as event for TTNT. Alive patients for whom no subsequent CLL/RT treatment is documented will be censored at the time of last observation they were assessed to be alive. Deceased patients for whom no subsequent CLL/RT treatment is documented will be censored at the date of death.
Adverse events (AE) will be classified using the Medical Dictionary for Regulatory Activities (MedDRA) classification system. The severity of the AEs will be graded according to the recent updated NCI CTCAE version 5.

Plants

Seed stocks	*Report on the source of all seed stocks or other plant material used. If applicable, state the seed stock centre and catalogue number. If plant specimens were collected from the field, describe the collection location, date and sampling procedures.*
Novel plant genotypes	*Describe the methods by which all novel plant genotypes were produced. This includes those generated by transgenic approaches, gene editing, chemical/radiation-based mutagenesis and hybridization. For transgenic lines, describe the transformation method, the number of independent lines analyzed and the generation upon which experiments were performed. For gene-edited lines, describe the editor used, the endogenous sequence targeted for editing, the targeting guide RNA sequence (if applicable) and how the editor was applied.*
Authentication	*Describe any authentication procedures for each seed stock used or novel genotype generated. Describe any experiments used to assess the effect of a mutation and, where applicable, how potential secondary effects (e.g. second site T-DNA insertions, mosiacism, off-target gene editing) were examined.*
